# Modeling individual time courses of thrombopoiesis during multi-cyclic chemotherapy

**DOI:** 10.1371/journal.pcbi.1006775

**Published:** 2019-03-06

**Authors:** Yuri Kheifetz, Markus Scholz

**Affiliations:** Institute for Medical Informatics, Statistics and Epidemiology, University of Leipzig, Leipzig, Germany; National University of Singapore, SINGAPORE

## Abstract

**Background:**

Thrombocytopenia is a major side-effect of cytotoxic cancer therapies. The aim of precision medicine is to develop therapy modifications accounting for the individual’s risk.

**Methodology/Principle findings:**

To solve this task, we develop an individualized bio-mechanistic model of the dynamics of bone marrow thrombopoiesis, circulating platelets and therapy effects thereon. Comprehensive biological knowledge regarding cell differentiation, amplification, apoptosis rates, transition times and corresponding regulations are translated into ordinary differential equations. A model of osteoblast/osteoclast interactions was incorporated to mechanistically describe bone marrow support of quiescent cell stages. Thrombopoietin (TPO) as a major regulator is explicitly modelled including pharmacokinetics and–dynamics of TPO injections. Effects of cytotoxic drugs are modelled by transient depletions of proliferating cells.

To calibrate the model, we used population data from the literature and close-meshed individual data of N = 135 high-grade non-Hodgkin’s lymphoma patients treated with CHOP-like chemotherapies. To limit the number of free parameters, several parsimony assumptions were derived from biological data and tested via Likelihood methods. Heterogeneity of patients was explained by a few model parameters. The over-fitting issue of individual parameter estimation was successfully dealt with a virtual participation of each patient in population-based experiments.

The model qualitatively and quantitatively explains a number of biological observations such as the role of osteoblasts in explaining long-term toxic effects, megakaryocyte-mediated feedback on stem cells, bi-phasic stimulation of thrombopoiesis by TPO, dynamics of megakaryocyte ploidies and non-exponential platelet degradation. Almost all individual time series could be described with high precision. We demonstrated how the model can be used to provide predictions regarding individual therapy adaptations.

**Conclusions:**

We propose a mechanistic thrombopoiesis model of unprecedented comprehensiveness in both, biological mechanisms considered and experimental data sets explained. Our innovative method of parameter estimation allows robust determinations of individual parameter settings facilitating the development of individual treatment adaptations during chemotherapy.

## Author summary

Chemotherapy is ubiquitously used to treat cancer diseases. Due to general toxicity of the drugs, chemotherapy results in a number of side effects especially with respect to blood formation. Here we study the loss of platelets during chemotherapy which is dose limiting in many situations. However, this side-effect greatly varies between patients with respect to both, severity and necessity of clinical countermeasures.We therefore developed a mathematical model to predict the time course of platelets of patients under chemotherapy and to propose possible treatment adaptations in cases of intolerable toxicity. The model is based on available biological knowledge and data of platelet formation and therapeutic effects thereon. As a major result, we could describe individual time series data of 135 patients under chemotherapy. Conversely, the model can be used to make predictions regarding alternative therapy schedules such as postponement of therapy or chemotherapy dose reductions. Our model is intended to support clinical decision making on an individual patient level.

## Introduction

Reduced platelet counts, called thrombocytopenia, is a major dose-limiting side effect of many dose-intense cancer chemotherapies [1,2]. Understanding thrombopoiesis during cytotoxic chemotherapy is crucial for its amelioration by chemotherapy dose adjustments, therapy postponement, platelet transfusion or growth factor applications such as thrombopoietin (TPO). However, this is a non-trivial task since thrombocytopenia risk depends on several therapy-based and individual factors such as dosing and timing of the cytotoxic drugs, application of platelet concentrates, age, sex and individual chemosensitivity [1,3]. A major challenge of precision medicine is to take all of these factors into account for optimal risk management. Otherwise, possibly small groups of patients with particularly worse outcome or side-effects impose therapy constrains for large patient collectives. Due to the large number of factors influencing therapy outcome and side-effects, we hypothesize that comprehensive models are required to support the concept of precision medicine.

In the present paper, we construct a comprehensive biomathematical model of human thrombopoiesis under chemotherapy, which allows prediction of time courses at an individual level for the first time. Our model is based on an earlier proposed model of human thrombopoiesis under chemotherapy [4] constructed to explain median time courses of patients. We call this model the ‘former model’ and propose a refinement here; based on the assumption of heterogeneity of a few model parameters rather than mechanistic differences between patients. Required additional model assumptions and corresponding adaptations of equations will be presented and discussed in detail.

Some emphasis is placed on parametrizing the model on the basis of available time series data of patients under therapy. We propose a Bayesian approach to include both, individual data and population data into the model fitting. To avoid over-fitting, we successfully addressed the problem of parameter identifiability, i.e. model selection is based on a generic, data-driven tradeoff between parsimony and precision.

Individualized model predictions require a detailed clinical data base. We used data of patients treated in the framework of randomized clinical trials of the German non-Hodgkin’s lymphoma study group guaranteeing high quality of individual patient data. These comprise therapy adaptations, supportive treatments, and most importantly, closely meshed time series of blood counts. Hence, we do not only consider heterogeneity in individual model parameters but also heterogeneity of treatment for the first time.

## Materials and methods

### Ethics statement

Ethics approval and consent to participate: Data were obtained from studies of the German High-Grade Non-Hodgkin’s Lymphoma Study Group. All patients had given informed consent and studieswere approved by responsible ethics committees and were carried out in accordance with the principles of good clinical practice and the declaration of Helsinki. Details on ethics committees and reference numbers can be found in the respective publications of the studies used for our modelling [5,6].

### The former model—Compartments and import regulatory feedbacks

The model proposed in the present paper is a modified and improved version of our previous work [4], which we briefly summarize here. This ordinary differential equations (ODE) model describes the dynamics of concatenated cell compartments of stem cells, colony-forming units of megakaryocytes, megakaryocytes and platelets in spleen and circulation. It contains several feedback loops where TPO is a major mediator. The model already considered the effect of cytotoxic chemotherapy by assuming transient depletion of bone marrow cell compartments after application. The principle structure is displayed in the figure in S1 Appendix. We briefly present the major features of the model. Then, necessary adaptations are described in detail:

1. Bone marrow thrombopoiesis originates from proliferating pluripotent stem cells (model compartment S), which commit to thrombopoietic lineage by differentiating into colony-forming units of megakaryocytes (CFU-MK). These cells are still capable of cell divisions. Examples are CD34^+^ precursors and promegakaryocytes, whose in vitro dynamics as well as apoptotic reactions to chemotherapy were described in [7] and [8]. These cells are summarized in model compartment CM.
2. In the former model, self-renewal of stem cells was represented by a probability *p* that a stem cell stays in compartment S after division. With probability *1-p* the cell enters CM. Self-renewal is negatively regulated by the relative count of stem cells and positively regulated by the relative number of differentiated cells representing the demand of mature cells.
3. CM differentiate to polyploid megakaryocytes (MKC). The former model does not distinguish between stages of MKC maturation, i.e. ploidy.
4. Platelet production rate was assumed to be proportional to the volume of megakaryocytes. The mass is proportional to the number and ploidy of megakaryocytes.
5. Platelets are released from bone marrow compartment MKC to peripheral blood. Young platelets (proplatelets) are preferentially sequestered in the spleen [9]. Platelets are age-dependently released from the spleen to the circulation [10]. Platelets in circulation have seven consecutive compartments (PLC) representing their aging.
6. The growth factor thrombopoietin (TPO) stimulates CM proliferation, increases the number of MKC endomitoses and accelerates MKC maturation.
7. TPO is actively consumed by MKC in bone marrow and platelets in blood resulting in a negative feedback of the system. TPO is also cleared from the system via kidneys. TPO is produced in the liver and the kidneys at constant rate [11–13].
8. Application of chemotherapeutic drugs induces a first order depletion of each bone marrow cell stage for the duration of one day. The bone marrow damage is reversible. No long-lasting deterioration of hematopoiesis is assumed in the former model, specifically, the dynamic parameters of the model are not affected by chemotherapy.

In our model, we also adopted the formalism developed in [14] regarding modelling of delayed transitions between compartments. Briefly, this is achieved by introducing a number of concatenated sub-compartments with first-order transitions mimicking a Gamma-distributed overall transition rate.

### Revisions required for individualized modelling

**Fig 1:**
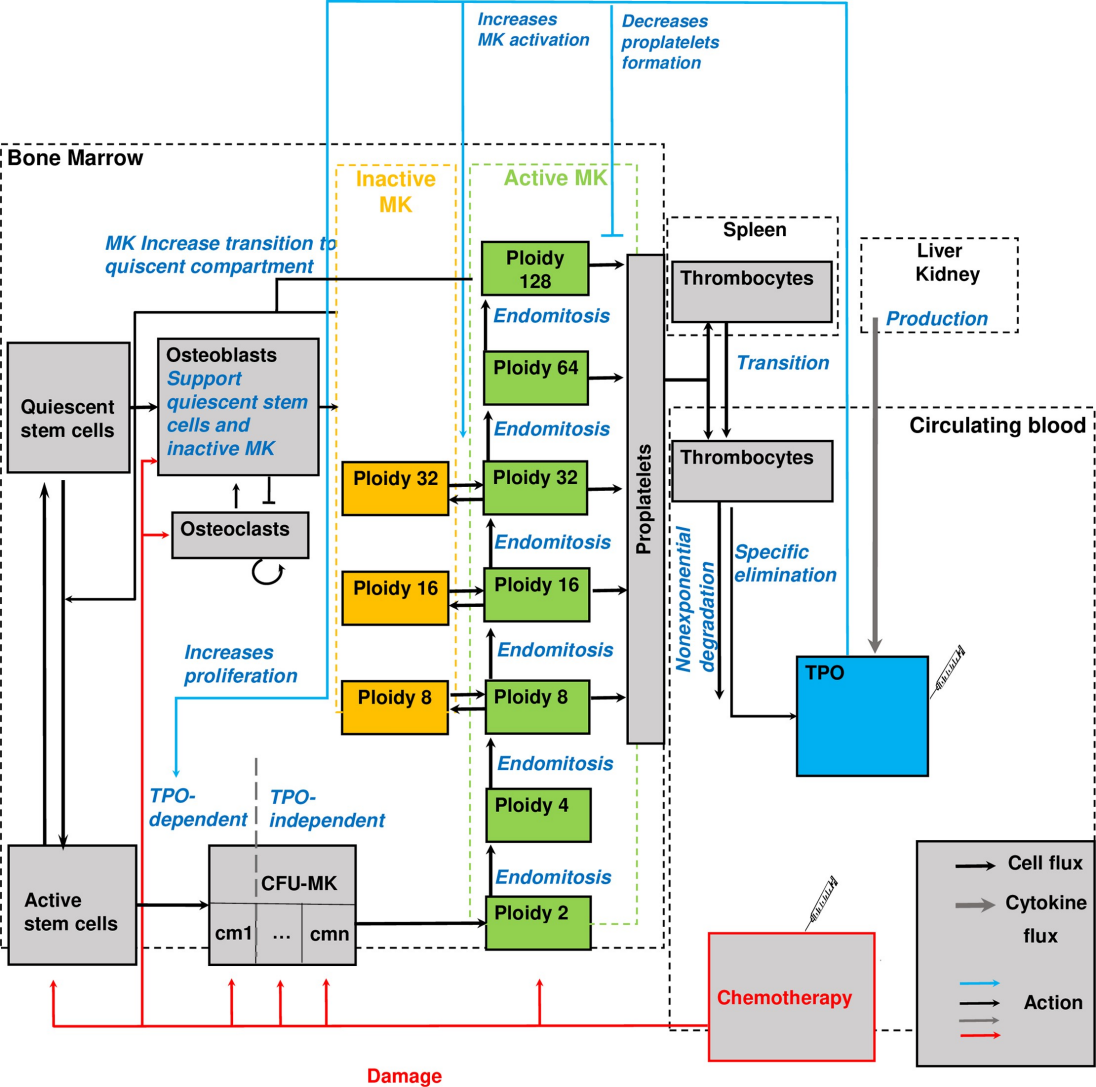
Modified structure of the human cell-kinetic thrombopoiesis model. We present all model compartments (boxes) and cell or cytokine fluxes or actions between them (arrows). Chemotherapy is modelled by a transient depletion of proliferating cell compartments. Syringes indicate possible injections. CM = colony forming units of megakaryocytes, MK = megakaryocytes, TPO = thrombopoietin. TPO action is shown in blue, chemotherapy-induced damage is shown in red.

Our former model correctly described median time courses of platelets of patients treated with chemotherapy. Individual deviations from the standard therapy were neglected and heterogeneity of patient responses was not considered so far. To remove these restrictions, we present an improved version of the model (Fig 1, description of model compartments, see Table 1). This however required a number of adaptations of model hypotheses explained and motivated in the following:

1. We revised the stem cell model according to recent experimental and theoretical results [15–17]. In detail, we introduced a quiescent stem cells compartment. Its balance with the corresponding proliferating compartment (active stem cells) is regulated by MKC. Thus, the former phenomenological feedback of differentiated cells on stem cells is now explicitly modeled. We assume that higher MKC numbers increase the transition of active to quiescent stem cells effectively limiting further production of megakaryocyte progenitors [18,19].
2. Osteoblasts maintain both, quiescent stem cells and quiescent megakaryocytes, and thus, are necessary for their survival. For the first time, we consider osteoblasts as a capacity parameter, i.e. higher osteoblast count implies a higher bone marrow capacity for both cell types. Osteoblast dynamics are based on a complex autocrine and paracrine interaction with osteoclasts. A simple model of this interaction was proposed by Komarova et al [20] and is incorporated into our revised model.
3. Multi-cyclic chemotherapy induces not only a loss in proliferating bone marrow cells but also in osteoblasts [21] and osteoclasts. This results in a long-term reduction of quiescent stem cells and megakaryocytes, and with it, in cumulative toxicity of chemotherapy and a delayed recovery.
4. We subdivided the compartment CM in order to model maturation.
5. We divided MKC into sub-compartments of different ploidies (from 2 to 128), which can be either active or inactive.
6. The cytoplasm of megakaryocytes buds to proplatelets from which platelets are formed [22–24]. We assume that MKC start emitting platelets if ploidy is greater than 4 and that the total production is still proportional to megakaryocyte mass [25].
7. The effects of TPO on the regulation of CM and MKC were revised: TPO stimulates amplification of early CM, increases ploidy of MKC, activates quiescent MKC and suppresses platelet formation.
8. We now consider external applications of peg-TPO by constructing and implementing a corresponding pharmacokinetic (PK) / pharmacodynamics (PD) model.
9. Platelet degradation is now described by a more complex non-exponential mechanism in accordance to Hanson and Slichter [26].

**Table 1:** (Description of model compartments). We describe the compartments of the model and their biological meaning.

Compartment name	Description
*C*_*OB*_	Osteoblast count
*C*_*OC*_	Osteoclast count
*C*_*S_act*_	Active (proliferating) stem cells
*C*_*s_dorm*_	Dormant stem cells
*C*_*CM*_	Compartment of megakaryocyte precursors, contains n_CM_ sub-compartments {C_CM,I_}_i_. The *n*_*CM*_^*e*^ early compartments depend on TPO, the *n*_*CM*_^*l*^ late compartments are TPO-independent
*C*_*MKC_act*,*PX*_	Active megakaryocytes with ploidy X, X takes values from {2,4,8,16,32,64,128}
*C*_*MKC_dorm*,*PX*_	Inactive (dormant) megakaryocytes with ploidy X, X takes values from {8,16,32}
*C*_*PP*_	Compartment of proplatelets
C_PLC_	Circulating platelets, contains n sub-compartments {C_PLC,i_} corresponding to age
C_PLS_	The spleen platelets compartment, contains n sub-compartments {C_PLS,i_} corresponding to age
$C_{PLC}^{l}$	A compartment of labeled transfused circulating platelets, contains n age-compartments {$C_{PLC,i}^{l}$}. This compartment was only introduced to explain the data of [26]. It is empty / not required for all other scenarios and data sets
*PL*	Sum of platelets from all circulating age-compartments
C_TPO,endo_	Endogenous TPO
*C*_*TPO*,*peg*_	Pegylated TPO. Consists of 3 sub-compartments: injection compartment *C*_*TPO*,*peg*,*0*_, lymphatic absorption compartment *C*_*TPO*,*peg*,*1*_ and compartment *C*_*TPO*,*peg*,*2*_ representing TPO in circulation.
*Del*_*TPO*,*rel*_	Delay compartment of TPO action on MKCs
*Del*_*TPO*,*rel*,2_^5^	A longer delay was necessary for activation of dormant MKC of ploidy 32
*OST*_*loss*_	Long-term cytotoxic effect on osteoblasts / osteoclasts
*C*_*cyclo*,1_	Concentration of cyclophosphamide in the first compartment (blood)
*C*_*doxo*,i_	Concentration of doxorubicine in the i-th compartment
*C*_*etop*,i_	Concentration of etoposide in the i-th compartment
*C*_*procar*,1_	Concentration of procarbazine in the first compartment (blood)

### Model equations

In this section, we derive all model equations. Corresponding parameters, their values and procedures for estimation are given in tables in S2 Appendix.

#### Chemotherapy model

Toxicity of chemotherapy plays a role in the majority of cell compartments. Therefore, we introduce corresponding model equations first. The former model [4] assumed that chemotherapy applications result in a first order loss within the compartments S, CM and MKC for the duration of 1 day. A later improvement of chemotherapy modelling assumed a non-constant toxic effect with a peak at some time point after injection. Toxic effects of multiple drug applications were added [4]. We adopt these principles in the following. In our modeled scenarios (see below), four different drugs with relevant haematotoxic potential are considered, namely cyclophosphamide, doxorubicine, procarbazine and etoposide. All cytotoxic drugs are assumed to affect the compartments S, CM, immature MKC (ploidies 2–4), mature MKC (ploidies 8–128) and osteoblasts / osteoclasts but with different strength. Consequently, the chemotherapy toxicity functions Ψ_Y_ of the drug X and compartment Y read as follows:

$$\Psi_{Y}=\sum_{X,Y}{pd}_{X,Y}∙C_{X,1},$$ (1)

where *C*_*X*,*1*_ represents the concentrations of corresponding drugs in the central (first) compartment considering pharmacokinetic models proposed for the drugs (see S3 Appendix for corresponding equations). Parameters *pd*_*X*,*Y*_ represent the contribution of the respective drug *X* to the toxicity function of Y-th compartment (pharmacodynamic effect). We assumed the same toxicity function for osteoblasts and osteoclasts indicating it with a subscript *OST*. We made a number of parsimony assumptions to reduce the number of chemotherapy-related parameters (see S4 Appendix).

#### Regulatory mechanisms

Amplification rates and transit times are regulated in a specified range in dependence on TPO. Total amplification is split to the respective amplifications of influx and efflux as proposed in the former model (see S5 Appendix). We used the following (modified) sigmoidal Z-functions for that purpose:

$$Z(X;y_{min},y_{nor},y_{max},b_{Y},{Lim}_{sig})=y_{max}-(y_{max}-y_{min})∙{\left(\frac{y_{max}-y_{nor}}{y_{max}-y_{min}}\right)}^{{Trans}_{tanh}(X^{b_{Y}},{Lim}_{sig})},$$ (2)

$${Trans}_{tanh}(X,{Lim}_{sig})=exp\left({Lim}_{sig}∙tanh(\frac{ln(X)}{{Lim}_{sig}})\right),$$ (3)

where *X* corresponds to TPO levels, *y*_*min*_, *y*_*nor*_, *y*_*max*_, to minimal, normal (steady-state) and maximal values of the respective quantity *Y* and *b*_*y*_ to the steepness of the regulatory function. This parameter requires restriction to avoid numerical issues. Therefore, we introduce the auxiliary function *Trans*_*tanh*_ which behaves like an identity function for *X* close to one. See S6 Appendix for a more detailed discussion of the properties of this class of sigmoid functions.

#### Cell-kinetic model

In this section, we establish the ODE system describing the dynamics of bone marrow thrombopoiesis and platelets. Initial values for all state variables are set to the corresponding steady-state values derived in S10 Appendix.

#### Osteoblasts, osteoclasts and capacity of bone marrow

Since osteoblasts are known to support inactive bone marrow cells [21,27], we assume that the relative osteoblast count $C_{OB}^{rel}$ determines the capacity of bone marrow to support different types of inactive cells. Likewise, this capacity limits influxes of cells from active compartments. Dormant cells die if the capacity is reduced.

To model the dynamics of osteoblasts, we adopt a model of Komarova et al [20] describing the interaction of osteoblasts and osteoclasts. The model does not consider proliferating precursors of osteoblasts and osteoclasts, which are mainly affected by cytotoxic drugs. To avoid further model complexity, we assume a delayed loss of osteoblasts and osteoclasts due to chemotherapy with delay parameter *D*_*Ψ*_. The delay is in the order of a few days. We further assume that the chemotherapy effect *Osteo*_*loss*_ is the same for both cell types:

$$\begin{array}{l} \frac{d}{dt}C_{OC}=\alpha_{1}∙C_{OC}^{0.5}∙C_{OB}^{-0.5}-\beta_{1}∙C_{OC}-{Osteo}_{loss}∙C_{OC}\\ \frac{d}{dt}C_{OB}=\alpha_{2}∙C_{OC}-\beta_{2}∙C_{OB}-{Osteo}_{loss}∙C_{OB}\\ \frac{d}{dt}{Osteo}_{loss}=\Psi_{Osteo}-D_{\Psi }∙{Osteo}_{loss} \end{array}$$ (4)

with *Osteo*_*loss*_(0) = 0. For further simplification and due to lack of data, we assumed that the toxicity function of precursors of osteoblasts and osteoclasts is proportional to that of the proliferating precursors CM, i.e. Ψ_*Osteo*_ = *c*_*PD*,*Osteo*_Ψ_*CM*_.

The relative osteoblast count is assumed to correspond to the bone-marrow capacity of dormant cells:

$${Cap}_{dor}=C_{OB}^{rel}\equiv \frac{C_{OB}}{C_{OB\_nor}}.$$ (5)

#### Stem cell compartment

The stem cell compartment is divided into two sub-compartments: quiescent (Q) and actively proliferating stem cells (S). This is based on a well-established model of stem cell regulation which was proposed a decade ago [16]. We assume that quiescent cells are not affected by chemotherapy [15,16].

Recent studies [18,19] showed that megakaryocytes stimulate quiescence of stem cells and are essential for their maintenance. Thus, we assume that megakaryocytes promote the transition from active to quiescent stem cell compartment. A bone marrow capacity *Cap*_*dor*_ is now included into the equations limiting this transition:

$$\begin{array}{l} \frac{d}{dt}C_{S\_act}=(2p-1)∙\frac{C_{S_{act}}}{T_{cycle}}+k_{act}∙C_{S_{dorm}}-{Cap}_{dor}∙k_{dorm}∙C_{S_{act}}-\Psi_{S}∙C_{S_{act}}\\ \frac{d}{dt}C_{S\_dorm}={Cap}_{dor}∙k_{dorm}∙C_{S\_act}-(k_{act}+d_{{Osteo}_{loss}}∙{Osteo}_{loss}∙C_{OB}^{rel})∙C_{S_{dorm}} \end{array}$$ (6)

$$k_{dorm}=k_{dorm,nor}∙C_{TTB}^{rel}(t)$$ (7)

$$C_{TTB}^{rel}=\frac{\sum_{i=1}^{7}C_{MKC\_{act,2}^{i}}+\sum_{i=3}^{5}C_{MKC\_{dorm,2}^{i}}}{\sum_{i=1}^{10}C_{MKC\_{act,2}^{i}}^{nor}+\sum_{i=3}^{5}C_{MKC\_{dorm,2}^{i}}^{nor}}$$ (8)

$C_{TTB}^{rel}$ serves as a mediator of the MKC feedback on stem cells. The megakaryocytes sub-compartments in (8) are explained in the related section below (see Eqs (14–19)). The parameter *k*_*dorm*_ represents the MKC-regulated transition of active cells to the dormant compartment. As long as the number of MKCs is larger than in steady-state, there is an increased flux of active towards dormant cells. The reverse transition rate *k*_*act*_ is assumed to be MKC- independent. Actually, megakaryocytes can control both directions, but the mechanism is less clear [18]. Moreover, from the modelling perspective, it can hardly be distinguished whether one or both directions are affected. An attempt to introduce the megakaryocytes-based control in both directions led to overfitting during parameters estimations. An attempt to model *k*_*dorm*_ as a sigmoid function of $C_{TTB}^{rel}$ also leads to overfitting.

We assume that dormant stem cells die due to chemotherapy-induced loss of osteoblasts with rate $d_{{Osteo}_{loss}}∙{Osteo}_{loss}∙C_{OB}^{rel}$. However, if $d_{{Osteo}_{loss}}$ is small, chemotherapy mobilizes stem cells and megakaryocytes due to reduced capacity *Cap*_*dor*_ in accordance with the observation that small doses of cytotoxic drugs can activate stem cells.

Compared to our former model, we simplified the regulation of self-renewal probability *p* which now depends only on the relative count of stem cells:

$$\begin{array}{l} p=p_{\delta }∙tanh\left(\frac{1}{{\left(C_{S\_act}^{rel}(t)\right)}^{b_{S\_act}}}-{\left(C_{S\_act}^{rel}(t)\right)}^{b_{S\_act}}\right)+0.5\\ C_{S\_act}^{rel}=\frac{C_{S\_act}}{C_{S\_act}^{nor}} \end{array}.$$ (9)

It is assumed that *p*_*δ*_ = *p*^*nor*^ − *p*^*min*^ = *p*^*max*^ − *p*^*nor*^ = 0.1, according to. The term in parenthesis of (9) is a decreasing function of $C_{S\_act}^{rel}$ taking values in the interval (−∞,∞) for $C_{S}^{rel}(t)\in (0,\infty )$. Thus, in analogy to the former model, *p* is regulated in between [0.15,0.85] and equals 0.5 in steady state. The parameter *b*_*S*_*act*_ is a new sensitivity parameter, which we use for fine-tuning of this regulatory feedback.

According to [28] the typical cell cycle time of eukaryotic cell is about 24 hours. This also applies for the human situation where the cell cycle is close to the circadian cycle [29]. Thus, we assumed that *T*_*cycle*_ = 24 hours for active stem cells. According to animal data [17], stem cell renewal takes 3–30 weeks on average (or once per 21–210 days). Since cell cycle lasts one day, it follows that there are 20 to 209 dormant stem cells per active cell. We derive the following steady state condition from (6):

$$\frac{C_{S_{act}}}{C_{S_{dorm}}}=\frac{k_{dorm}}{k_{act}}\equiv {rk}_{dorm_{act}}\in [20,209].$$ (10)

We estimate *k*_*dorm*_ and *rk*_*dorm_act*_ parameters and derive *k*_*act*_ from this relation, i.e. the above-mentioned considerations restrict the range of the ratio of *k*_*dorm*_ and *k*_*act*_.

#### CM compartment

According to observations of megakaryocytes and its progenitors, we assume that cells in CM cycle within a fixed time of 15 hours [22–24]. We assume that TPO regulates the number of cell divisions in CM compartment. For this purpose, we divided the compartment into *n*_*CM*_ sub-compartments. The transit time *T*_*CM*_ is the logarithm dualis of the proliferation (amplification) A_CM_ (see eq. (S.7.3) in the S7 Appendix). The latter increases with TPO levels:

$$\frac{d}{dt}C_{CM,i}=A_{CM,i}^{in}∙C_{X,i}^{out}-\frac{C_{CM,i}}{T_{CM,i}}-\Psi_{CM}∙C_{CM,i},i=1,\cdots ,n_{CM}$$ (11)

$$C_{X,1}^{out}=C_{S}^{out}=2∙(1-p)∙\frac{C_{S}}{T_{cycle}},\;C_{X,i}^{out}=C_{CM,i-1}^{out}=A_{CM,i-1}^{out}∙\frac{C_{CM,i-1}}{T_{CM,i-1}},\;i=2,\cdots ,n_{CM}.$$ (12)

It was observed by Harker et al. [30] that injection of pegylated TPO into healthy subjects results in delayed peaks in the count of megakaryocytes and platelets (both at day 11 after peg-TPO). Moreover, an artificially induced thrombocytopenia increases megakaryocytes ploidy in rats [13]. Megakaryocyte size and ploidy rise to its maximum at days 2–4. In contrast, megakaryocyte number is highest after 10 days. In other words, endomitoses of megakaryocytes are stimulated immediately by TPO increase while numbers increase with some delay. To explain these observations, we assume that TPO stimulates proliferation of early CM sub-compartments only. We distinguish between early and late CM sub-compartments for that purpose:

$$n_{CM}=n_{CM}^{e}+n_{CM}^{l}.$$ (13)

Only in the $n_{CM}^{e}$ early sub-compartments, the amplification depends on TPO.

Since we assume fixed cell cycle times, the transit time for the sub-compartments of CM as well as their amplifications are calculated from the number of cell divisions *n*_*CM*_. Detailed equations can be found in S7 Appendix.

#### MKC compartment

We also improved our model of megakaryocyte dynamics. We present the underlying biological observations in the following and explain how they are transformed into model equations. According to Santillan et al. [25] we assume that megakaryocytes of different ploidies mature in parallel to platelets. Most of the following hypotheses regarding this process are derived from the data of Harker et al. [30] in which dynamics of megakaryocyte counts as well as dynamics of ploidy frequency distributions after injection of pegylated TPO were studied. In this study, the overall TPO peak occurred at day 2 and drops to endogenous TPO levels at days 7–9 after injection. We summarize the main observations:

1. The authors detected ploidy frequencies ranging from 1 to 64 in steady state. A few megakaryocytes of ploidy 128 are detected at day 7 after TPO injection. All observed ploidies are powers of 2.
2. Megakaryocyte count increases slightly until day 7, doubles until day 11 and decreases slightly below initial values until day 17.
3. Ploidies performed differently after TPO injection:
  - Megakaryocytes of ploidies 2 and 4 have similar frequencies and dynamics. Their frequencies reach peak levels at day 11 (8–11%) and return to slightly elevated frequencies at day 17.
  - Ploidy 8 shows similar dynamics with a more prominent peak at day 11 and considerably increased frequency (from 19% at steady state to 32% at the peak).
  - Megakaryocytes of ploidies 16 and 32 are dominant with baseline levels of 47% and 21%, respectively. Their dynamics is opposite to the other ploidies: These ploidies almost halves after TPO stimulation with minima at days 7 and 11, respectively. Percentages returned to steady-state values at day 17.
  - Megakaryocytes of ploidy 64 are rare. Frequency increases from 0.6% to 3.5% at day 7 and drops below normal levels at day 11. Ploidy 128 is also rare, achieving a value of 0.22% at day 7. Since platelet production is roughly proportional to ploidy of megakaryocytes, even low frequencies of ploidies 64 and 128 contribute significantly to thrombopoiesis. Thus, these ploidies are also considered in our model.
4. Megakaryocyte count is strongly increased by TPO and doubles at day 11. Counts drop to values slightly below normal at day 17.
5. Platelet counts decrease slightly during the first three days after TPO injection. It starts to exceed baseline level after day 5 when the TPO level almost returned to its initial value. Then platelet count is increased for several weeks.

There is an additional morphologically identifiable proplatelet stage [31], which consists of platelets forming from the cytoplasm of megakaryocytes. This process was observed in real time and has shown to be reversible to some extent [22–24]. These observations translate into the following mechanistic model assumptions:

1. According to direct MKC observations *in vitro* [22–24], megakaryocytes endomitosis and blast mitosis times are estimated to be about 15 hours irrespective of MKC ploidies. Thus, the transit times of ploidy states are assumed to be equal. Certain MKC stop endomitosis and die during 1.5–2 days.
2. Since frequencies of megakaryocytes of ploidies 8, 16 and 32 are considerably higher than those of the other ploidies (point 3c from observation summary above), average endomitosis cycles are assumed to be slower for ploidies 8, 16 and 32. To model this phenomenon, we assume that megakaryocytes can be either in active state undergoing endomitoses or in passive (dormant) state, in which they cannot live without bone marrow support for more than 1.5–2 days (in vitro conditions). Exchange rates between active and dormant states determine their relative frequencies. Bone marrow support is modeled in analogy to the stem cell compartment, i.e. via osteoblast support.
3. Cells from active MKC sub-compartments of ploidies 8, 16 and 32 have 3 possible fates:
  - move to the proplatelet compartment with respective probabilities p_i,1_
  - move to the next-ploidy compartment with respective probabilities (1-p_i,1_)· p_i,2_
  - move to the corresponding inactive compartment with respective probabilities (1-p_i,1_)· (1-p_i,2_), i = 8, 16, 32. We fixed the transit time of this process *T*_*dorm*,*MKC*_ to 12h due to lack of detailed biological data.
  Cells from the (active) MKC sub-compartment of ploidy 64 have two possible fates:
  - move to the next-ploidy compartment with probability 1-p_64,1_ which exceeds zero only if the TPO level is elevated.
  - move to the proplatelet compartment with probability p_64,1_.
4. Cells of inactive compartments of ploidies 8–32 can only return to active compartments of the same ploidy or die due to lack of bone marrow support.
5. TPO-induced decrease in relative frequencies of megakaryocytes of ploidies 16 and 32 can be explained by mobilization of dormant megakaryocytes and their subsequent elimination during proplatelet formation. This TPO property is in accordance to the earlier assumption that the megakaryocyte transit time is negatively regulated by TPO [32].
6. In addition to the above mentioned 10 sub-compartments of active and dormant megakaryocytes, we model the proplatelet sub-compartment *C*_*PP*_ with short transit time *T*_*PP*_ [22–24]. Since dynamics of frequencies of megakaryocytes of ploidies 2 and 4 are similar and since their frequencies are much lower than those of ploidies 8–32, we assume that these megakaryocytes have no dormant state and cannot form proplatelets.
7. Since platelet counts drop immediately after TPO injection, we hypothesize that TPO stimulation increases probabilities of endomitoses at the expense of proplatelet formation. This regulation is indirectly supported by the above mentioned observation that proplatelet formation is reversible.
8. We introduce a time delay of TPO action on MKC development in analogy to delayed G-CSF action in our granulopoiesis models [33]. The delay is modelled by a lag compartment *Del*_*TPO*,*rel*_ with first order transition. The efflux of this compartment is the delayed TPO action, which serves as the argument of our regulatory Z-functions.
9. Chemotherapy is assumed to affect active MKCs but not proplatelets. Chemotherapy affects dormant MKC indirectly through elimination of supporting osteoblasts as in the case with quiescent stem cells.

According to these assumptions, the ODEs for the 10 MKC sub-compartments and 1 proplatelet compartment are as follows:

$$\frac{d}{dt}C_{MKC,act,P2}=A_{CM,n_{CM}}^{out}∙\frac{C_{CM,n_{CM}}}{T_{CM,n_{CM}}}-\frac{C_{MKC,act,P2}}{T_{endo}}-\Psi_{MKCimm}∙C_{MKC,act,P2}$$ (14)

$$\frac{d}{dt}C_{MKC,act,P4}=\frac{C_{MKC,act,P2}}{T_{endo}}-\frac{C_{MKC,act,P4}}{T_{endo}}-\Psi_{MKCimm}∙C_{MKC,act,P4}.$$ (15)

For k = 3, 4, 5 we obtain:

$$\begin{array}{c} \frac{d}{dt}C_{MKC,act,P2^{k}}=\frac{p_{2^{k-1},2}∙(1-p_{2^{k-1},1})}{T_{endo}}∙C_{MKC,act,P2^{k-1}}{+k}_{rev_{dorm},2^{k}}∙C_{MKC,dorm,P2^{k}}-\\ \left(\frac{p_{2^{k},2}∙(1-p_{2^{k},1})}{T_{endo}}+\frac{p_{2^{k},1}}{T_{PP}}+k_{dorm,2^{k}}+\Psi_{MKC}\right)∙C_{MKC,act,P2^{k}} \end{array}$$ (16)

$$\begin{array}{l} \frac{d}{dt}C_{MKC,dorm,P2^{k}}=k_{dorm,2^{k}}∙C_{MKC,act,P2^{k}}-\\ \;(k_{rev_{dorm},2^{k}}-d_{{Osteo}_{loss}}∙{Osteo}_{loss}∙C_{OB}^{rel})∙C_{MKC,dorm,P2^{k}} \end{array}.$$ (17)

For the case of k = 3, the term p_4,2_ · (1-p_4,1_) in (16) equals to 1, since we assume that MKC of ploidy 4 can move neither to dormant nor to proplatelet compartments. In complete analogy to dormant stem cells, we assume, that dormant MKC die, due to elimination of supporting adjacent osteoblasts, with the corresponding death rate $d_{{Osteo}_{loss}}∙{Osteo}_{loss}∙C_{OB}^{rel}$.

$$\frac{d}{dt}C_{MKC,act,P64}=\frac{p_{32,2}∙(1-p_{32,1})}{T_{endo}}∙C_{MKC,act,P32}-\left(\frac{\left({1-p}_{64,1}\right)}{T_{endo}}+\frac{p_{64,1}}{T_{PP}}+\Psi_{MKC}\right)∙C_{MKC,act,P64}$$ (18)

$$\frac{d}{dt}C_{MKC,act,P128}=\frac{\left({1-p}_{64,1}\right)}{T_{endo}}∙C_{MKC,act,P64}-\left(\frac{1}{T_{PP}}+\Psi_{MKC}\right)∙C_{MKC,act,P128}.$$ (19)

In S8 Appendix, the dependencies of probabilities *p*_*2*_^*k*^_,*1*_ for k = 3,4,5,6 on TPO are described in detail.

The transit rates from inactive to active MKC sub-compartments are given by:

$$k_{dorm,2^{k}}=\frac{\left(1-p_{2^{k},1})∙(1-p_{2^{k},2}\right)}{T_{dorm,MKC}}∙{Cap}_{dor},\;k=3,4,5$$ (20)

where *Cap*_*dor*_ is defined in (5). For the transit rates from the active to the inactive MKC sub-compartments (only valid for k = 3, 4, 5) we assume an indirect proportionality to the bone marrow support, i.e.

$$k_{rev_{dorm},2^{k}}=\frac{1}{{Cap}_{dor}}∙Z\left({Del}_{TPO,rel,2^{k}},k_{rev_{dorm},2^{k}}^{min},k_{rev_{dorm},2^{k}}^{nor},k_{rev_{dorm},2^{k}}^{max},b_{rev_{dorm},2^{k}},{Lim}_{sig}\right)$$ (21)

where transit rates are the reverse of transit times:

$$k_{rev\_dorm,2^{k}}^{nor}=\frac{1}{T_{rev\_dorm,2^{k}}^{nor}},\;k=3,4,5.$$ (22)

Several parsimony assumptions were made for the MKC compartment to reduce the number of free parameters (see S4 Appendix for details). A longer delay for TPO induced activation of MKC of ploidy 32 is assumed (${Del}_{TPO,rel,2^{k}}$, k = 5 as explained in S8 Appendix). For k = 3,4, ${Del}_{TPO,rel,2^{k}}$ is equal to *Del*_*TPO*,*rel*_.

#### Proplatelets compartment

All active MKC sub-compartments of ploidies 8–128 contribute to the proplatelet compartment in proportion to their ploidy. Proplatelets are then released to circulation:

$$\frac{d}{dt}C_{PP}=\frac{1}{T_{PP}}∙\sum_{k=3}^{7}\left(2^{k-1}∙C_{MKC,act,P2^{k}}\right)-\frac{C_{PP}}{T_{PP},}$$ (23)

where *T*_*PP*_ is the transit time of the proplatelet compartment.

The efflux of proplatelets to the circulating platelet compartment is:

$$C_{PP}^{out}={npt}_{pcu}∙\frac{C_{PP}}{T_{PP}}.$$ (24)

The parameter *npt*_*pcu*_ is the number of platelets produced per ploidy unit of megakaryocytes (i.e. per diploid chromosome set). It is roughly estimated that each megakaryocyte gives rise to about 400–8000 platelets on average [34]. Since modal megakaryocyte is of ploidy 16 in steady state [30], we estimate *npt*_*pcu*_ to be in between 125 and 1000.

#### Endogenous and pegylated TPO

**Fig 2:**
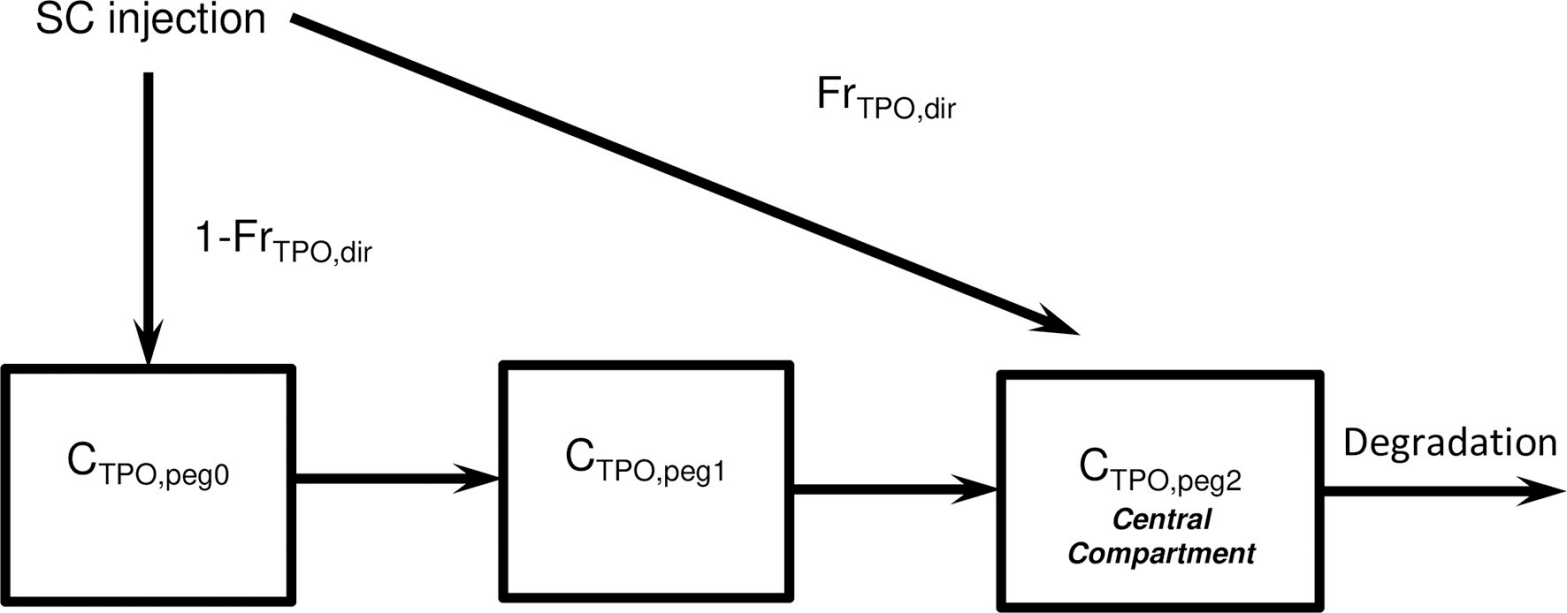
Absorption model of pegylated TPO injected as bolus subcutaneously. A fraction *Fr*_*TPO*,*dir*_ of the injection reaches the circulation directly. The rest of an influx of subcutaneously injected TPO is delayed by two compartments.

We now present our model assumptions and equations for the production and degradation of endogenous TPO, pharmacokinetics of pegylated TPO applications and corresponding effects on bone marrow thrombopoiesis:

1. In the previous model [4], only circulating platelets and MKC are supposed to consume TPO. Here we assume also that spleen platelets consume TPO.
2. Pegylated TPO is applied to prevent thrombocytopenia. Polyethylene glycol (PEG) polymer chains are attached covalently to TPO. PEG masks TPO for the host’s immune system, preventing serious immune reactions occasionally caused by pharmaceutical TPO [35,36].
  - Because of this modification, pegylated TPO could have a longer life span compared to endogenous TPO as well as a modified efficacy. However, our parsimony analysis revealed that the same transit time and PD effects can be assumed as for endogenous TPO.
  - We describe pegylated TPO dynamics after SC (subcutaneous) administration by two delay compartments in order to fit the shape of blood TPO dynamics after injection [30]. Fig 2 shows the assumed absorption model.

Based on these assumptions, we now construct the corresponding model equations. Subscripts *peg* and *endo* were introduced in the following to describe pegylated and endogenous TPO compartments.

#### Dynamics of endogenous and pegylated TPO

Pegylated TPO is assumed to be injected into the subcutaneous tissue. The absorption is modelled by two transit compartments *C*_*TPO*,*peg*0_ and *C*_*TPO*,*peg*1_ where *C*_*TPO*,*peg*0_ represents the compartment in which the drug is injected. We assume that a fraction *Fr*_*TPO*,*dir*_ of TPO directly enters the central compartment due to the force of the injection:

$$\begin{array}{l} \frac{d}{dt}C_{TPO,peg0}=-q_{TPO}∙{(1-{Fr}_{TPO,dir})∙C}_{TPO,peg0}\\ \frac{d}{dt}C_{TPO,peg1}=q_{TPO}∙(1-{Fr}_{TPO,dir})∙C_{TPO,peg0}-q_{TPO}∙C_{TPO,peg1}\\ Inj=q_{TPO}∙{{Fr}_{TPO,dir}∙C}_{TPO,peg0}+q_{TPO}∙C_{TPO,peg1} \end{array}.$$ (25)

The parameter *q*_*TPO*_ describes the flux rate of pegylated TPO from compartment to compartment. The last term of the second equation describes the influx into the central pegylated compartment *C*_*TPO*,*peg*2_ in which the drug is biologically active regarding thrombopoiesis. *Inj* is the influx resulting from TPO injections into the central compartment. The overall relative TPO concentration is given as:

$$C_{TPO}^{rel}=\frac{C_{TPO,endo}+C_{TPO,peg2}}{C_{TPO}^{nor}}=C_{TPO,end}^{rel}+C_{TPO,peg2}^{rel}.$$ (26)

TPO dynamics in the central compartment depends on endogenous TPO synthesis (*Synth*), TPO influx (*Inj*) from injections and TPO degradation (*Degr*).

We assume no difference between degradation rates of normal and pegylated TPO. Thus, TPO degradation is a function of total relative TPO concentration and degradation rates of relative endogenous and pegylated relative TPO concentrations are proportional to their respective fractions:

$$\begin{array}{l} \frac{d}{dt}C_{TPO,end}^{rel}=\frac{Synth}{C_{TPO}^{nor}}-\frac{C_{TPO,endo}}{C_{TPO,endo}+C_{TPO,peg2}}∙Degr(C_{TPO}^{rel})\\ \frac{d}{dt}C_{TPO,peg2}^{rel}=\frac{Inj}{C_{TPO}^{nor}}-\frac{C_{TPO,peg2}}{C_{TPO,endo}+C_{TPO,peg2}}∙Degr(C_{TPO}^{rel}) \end{array}.$$ (27)

According to Scholz et al., synthesis of TPO is constant with rate α and an unspecific degradation with rate *1/T*_*TPO*_ is assumed. The specific, receptor-mediated degradation is based on a Michaelis-Menten kinetic. Specific degradation is saturated regarding TPO and linear regarding relative platelets and megakaryocytes counts:

$$\frac{d}{dt}C_{TPO,end}^{rel}=q_{TPO}∙\frac{C_{TPO,peg1}}{C_{TPO}^{nor}}+\alpha -\frac{C_{TPO}^{rel}}{T_{TPO}}-\left(w_{PLC}∙\sum_{i=1}^{n}C_{{PLC}_{i}}+\sum_{k=1}^{7}\left(w_{MKC,k}∙C_{MKC,2^{k}}\right)+w_{PP}∙PP\right)∙\frac{C_{TPO}^{rel}}{1+k_{m,TPO}∙C_{TPO}^{rel}}.$$ (28)

A parameter *k*_*m*,*TPO*_ represents the saturation constant of the specific elimination regarding TPO concentration. *w*_*PLC*_ and *w*_*MKC*,*k*_, *w*_*PP*_ are the maximum specific elimination rates by platelets, megakaryocytes of ploidies 2^k^, k = 1,…128 and proplatelets, respectively. We assume equal TPO consumption by active and dormant megakaryocytes. Relations between corresponding eliminations are derived based on the receptor densities of platelets and megakaryocytes (see S9 Appendix).

Considering Eq (28) in steady-state, i.e. $C_{TPO}^{rel}=1$ yields:

$$\alpha =\frac{1}{T_{TPO}}+\frac{\left(w_{PLC}∙\sum_{i=1}^{n}C_{{PLC}_{i}}^{nor}+\sum_{k=1}^{7}\left(w_{MKC,k}∙C_{MKC,2^{k}}^{nor}\right)+w_{PP}∙{PP}^{nor}\right)}{\left(1+k_{m,TPO}\right)}.$$ (29)

Thus, the constant production is not a free parameter but given by the steady-state condition and other parameters.

#### Platelet compartments

The former model assumed that young platelets are preferentially sequestered in the spleen. They were age-dependently released to the circulation and were eventually degraded. These mechanisms are preserved in the present model. The compartments of spleen and circulating platelets are subdivided into *n* age compartments. The former model also assumed that platelet survival in circulation is approximately Gamma-distributed [37]. SR Hanson and SJ Slichter [26] studied platelet kinetics in patients with injured bone marrow function using platelet labeling. They observed that patients with thrombocytopenia have faster platelet turnover. Overestimation of the platelet levels occurs when a classical exponential decay is assumed. Therefore, a small constant consumption of platelets for vessel support was postulated [26]. A mathematical model of platelet survival based on these observations was later developed by another group [38]. This model assumed constant consumption by the blood system combined with linear loss and quadratic loss due to the capacity of the system. We adopted this model and combined it with our cell-kinetic model. It revealed that the quadratic loss term is badly identifiable and can be neglected. The constant elimination E is problematic for low platelet levels since it can result in negative ODE solutions. Consequently, we replaced it by a quasi-constant sigmoid function (i.e. a sigmoid function with a small range) $\frac{k_{s}∙{Circ}^{p}}{{Circ}^{p}+h_{s}^{p}}$, where *Circ* is the sum of all circulating platelet compartments, *k*_*s*_ is the maximum of the quasi-constant term and *h*_*s*_ is a platelet count corresponding to the half-maximal quasi-constant consumption. The power *p* is assumed to be larger than 1. Thus the quasi-constant consumption term is close to a constant for large *p* and platelet counts much greater than *h*_*s*._ For simplicity and due to a lack of data, we assume that the consumption is independent of the age of platelets, i.e. it is proportional to the relative content. Thus, for the *i-*th compartment we have the following quasi-constant elimination:

$$E_{i}(Circ)=\frac{C_{{PLC}_{i}}}{Circ}\frac{k_{s}∙{Circ}^{p}}{{Circ}^{p}+h_{s}^{p}}.$$ (30)

The linear term of the Hersh et al model roughly corresponds to transitions between the age-related sub-compartments of platelets with a corresponding transit time $T_{PL}^{sub}=\frac{T_{PL}}{n}$, where *T*_*PL*_ is the overall transit time. Thus, the balance equations of the platelet compartments read as follows (i = 1,…,n):

$$\frac{d}{dt}C_{{PLC}_{1}}=k_{circ}∙C_{PP}^{out}-\frac{C_{{PLC}_{1}}}{T_{PL}^{sub}}+k_{1}^{SC}∙C_{{PLS}_{1}}-E_{1}(Circ)$$ (31)

$$\frac{d}{dt}C_{{PLS}_{1}}=(1-k_{circ})∙C_{PP}^{out}-\frac{C_{{PLS}_{1}}}{T_{PL}^{sub}}-k_{1}^{SC}∙C_{{PLS}_{1}}$$ (32)

$$\frac{d}{dt}C_{{PLC}_{i}}=\frac{C_{{PLC}_{i-1}-C_{{PLC}_{i}}}}{T_{PL}^{sub}}+k_{i}^{SC}∙C_{{PLS}_{i}}-E_{i}(Circ)$$ (33)

$$\frac{d}{dt}C_{{PLS}_{i}}=\frac{C_{{PLS}_{i-1}-C_{{PLS}_{i}}}}{T_{PL}^{sub}}-k_{i}^{SC}∙C_{{PLS}_{i}},i=2,\cdots ,n$$ (34)

We remind that $C_{PP}^{out}$ is the efflux from the proplatelet compartment (24) while *k*_*circ*_ denotes the percentage of newly formed platelets entering circulation rather than sequestration in the spleen. The parameters $k_{i}^{SC}$ denote transition of aged platelets from spleen to circulation and are derived in S11 Appendix in complete analogy to our previous model [4]. All parameters of the model are listed in the tables of S2 Appendix.

To model the labelling data of Hanson et al. [26], we temporarily introduced a compartment of labeled platelets as shortly described in S12 Appendix. Patients with bone marrow hypoplasia were now considered as patients with low steady-state platelet counts.

### Clinical and biological data used for modelling

Simulation results of the model are compared with clinical data in order to verify the model assumptions and to estimate parameter settings. Data were either taken from the literature or are provided by the German non-Hodgkin’s lymphoma trial group (PI: Michael Pfreundschuh). These data comprise individual therapy settings and time series data of platelets.

### Biological data

Here we briefly present the literature data used for our model development. Typically, only averaged data are available.

**Data of Harker et al [30]**. Single doses of 3 μg/kg of pegylated TPO were injected subcutaneously into 16 healthy subjects. Total TPO concentrations and platelet counts were measured daily between day 0 and day 28. MKC counts and percentages of MKC ploidies were determined at days 0, 7, 11 and 17 after TPO injection.

**Data of Hanson and Slichter 1985 [26]**. Autologous ^51^Cr-labeled platelets were transfused to 16 normal subjects and 27 patients with stable, untreated thrombocytopenia secondary to bone marrow failure. Platelet counts range between 12,000 and 70,000/μL. Dynamics of labeled platelets were determined during 5 days after injection. Compared to normal subjects, platelet life span was slightly reduced in patients with platelet counts in between 50,000 to 100,000/μL but was markedly reduced for patients with platelet counts below 50,000/μL.

**Data of Li et al [21].** Ten cancer patients received four chemotherapy cycles in median (range 3–6 cycles). Their bone marrow niches have been examined before and after treatment. The number of osteoblasts per bone surface was markedly reduced after chemotherapy.

### Individual data of poly-chemotherapy-treated patients

**Data of Engel et al [39]**. Three patients with Hodgkin’s or aggressive non-Hodgkin lymphomas were considered in this study. Patients received intensified multi-cycle poly-chemotherapies. Close-meshed time series data of endogenous TPO and platelet counts were determined.

All three patients showed clear long-term effects of the therapy:

1. Average platelet levels gradually decrease from cycle to cycle.
2. Average TPO levels increases from cycle to cycle.
3. Platelet decline in first cycle is clearly less severe than in subsequent cycles.

**Data of NHL-B study [1,5,6].** The NHL-B study is a randomized clinical trial of elderly patients with aggressive non-Hodgkin’s lymphoma. Patients were randomized to one of the four arms 6xCHOP or 6xCHOEP with either 14 or 21 days of cycle duration. Schedules with 14 day cycle duration were supported by G-CSF injections. Thrombopenia was treated with platelet transfusions, postponement of therapy or reduction in chemotherapy dose.

Close meshed time series data of blood cell counts are available for these patients as well as individual information regarding the course of the therapy (i.e. individual risk factors, dosing of drugs and growth factors, therapy delays, supportive care, outcome). We selected 135 from 1600 patients, whose platelets counts were measured during 4 or more cycles with at least 5 measurements per cycle to obtain sufficiently detailed individual time series data.

### Model implementation and parameter estimation

Our ODE model (1–34) was implemented in Matlab and numerically solved with the 15s solver [40,41]. Simulation settings of specific therapy scenarios are explained in S3 Appendix. Parameter estimation is based on the optimization of the agreement of model and data as measured by fitness functions. Only a few parameters were assumed to differ between individuals explaining patient heterogeneity. These parameters are determined by optimizing individual fitness functions. In order to overcome the overfitting problem, each individual fitness function includes both, individual data as well as averaged data from the literature (called biological data in the following). This is equivalent to a *virtual participation* of a patient in the literature studies from which the data were retrieved. Details of the parameter estimation procedure and measures to avoid model overfitting are explained in detail in S13 Appendix. We performed a step-wise fitting process starting with Engel et al. data and including literature data. Finally, individual time course of patients from the NHL-B study are fitted.

### Steps of parameter estimation

We performed a stepwise fitting procedure to estimate the parameters of our model:

1. We first fitted available biological data and Engel et al. data simultaneously. Prior constrains were imposed only on parameters *r*_*PL*,*0*,*nor*_ and *r*_*TPO*,*nor*,*0*_ (see Table 1 in S13 Appendix) to ensure that the distribution of initial values matches that of the steady state values. The following steps were performed:
  - We assume different parameter settings (inter-individual variability–IIV) for the three patients considered in Engel et al. In particular, we initially assumed IIV for all parameters. We performed a backward selection process by gradually dropping IIV assumptions of parameters showing high uncertainty until minimum BIC was achieved. For model parsimony, we optimized the more conservative BIC rather than AIC.
  - We performed parsimony analysis of parameter sets to reduce the number of free parameters as much as possible. Parameters with very large relative error were fixed to biologically meaningful values. We used BIC (Bayesian Information criterion) for the choice of the parameter set for which patient heterogeneity is assumed. Parameters with related biology and low identifiability were set to the same values (for example, chemotherapy PD effects of S and CM compartments, PD effects of cyclophosphamide and doxorubicin, parameters of transition probabilities of MKC of ploidies 8, 16 and 32, transit times from inactive to active MKC of ploidies 16 and 32) in analogy to other models [42,43].
  - We re-estimated the remaining free parameters, in total, 22 parameters without patient heterogeneity (population parameters) and 9 parameters (see Table 1 in S13 Appendix) with assumed patient heterogeneity. Free parameters were selected by applying Bayesian Information criterion.
2. To fit the individual data of the 135 patients of the NHL-B study, we used the population and individual parameters estimated at step 1.c. The distributions of the individual parameters serve as priors for the NHL-B patients. Since no TPO data are available for these patients, the individual parameter of TPO steady-state *r*_*TPO*,*nor*,*0*_ is not required anymore to fit these data. This results in a total of 8 parameters assumed to be heterogeneous.

All parameters are described in tables from S2 Appendix. Further details of the fitting process can be found in S14 Appendix.

## Results

The results section is organized as follows: We first present the simultaneous fits of Engel et al and biological data and analyze their goodness of fits. We then compare parameter estimates between scenarios and analyze their identifiability. After these initial fittings of literature data, we proceed with the individual patient data of the NHL-B study. We modelled the individual platelets dynamics including long-term follow up for a few selected patients developing strong thrombocytopenia. Finally, we considered a number of possible individual therapy adaptations and predict their thrombopoenic outcome to demonstrate the clinical utility of our model. All parameter estimates are presented in S15 Appendix and S16 Appendix.

### Comparison of model simulations and data of Engel et al, Harker et al, Hanson et al and Li et al

**Fig 3:**
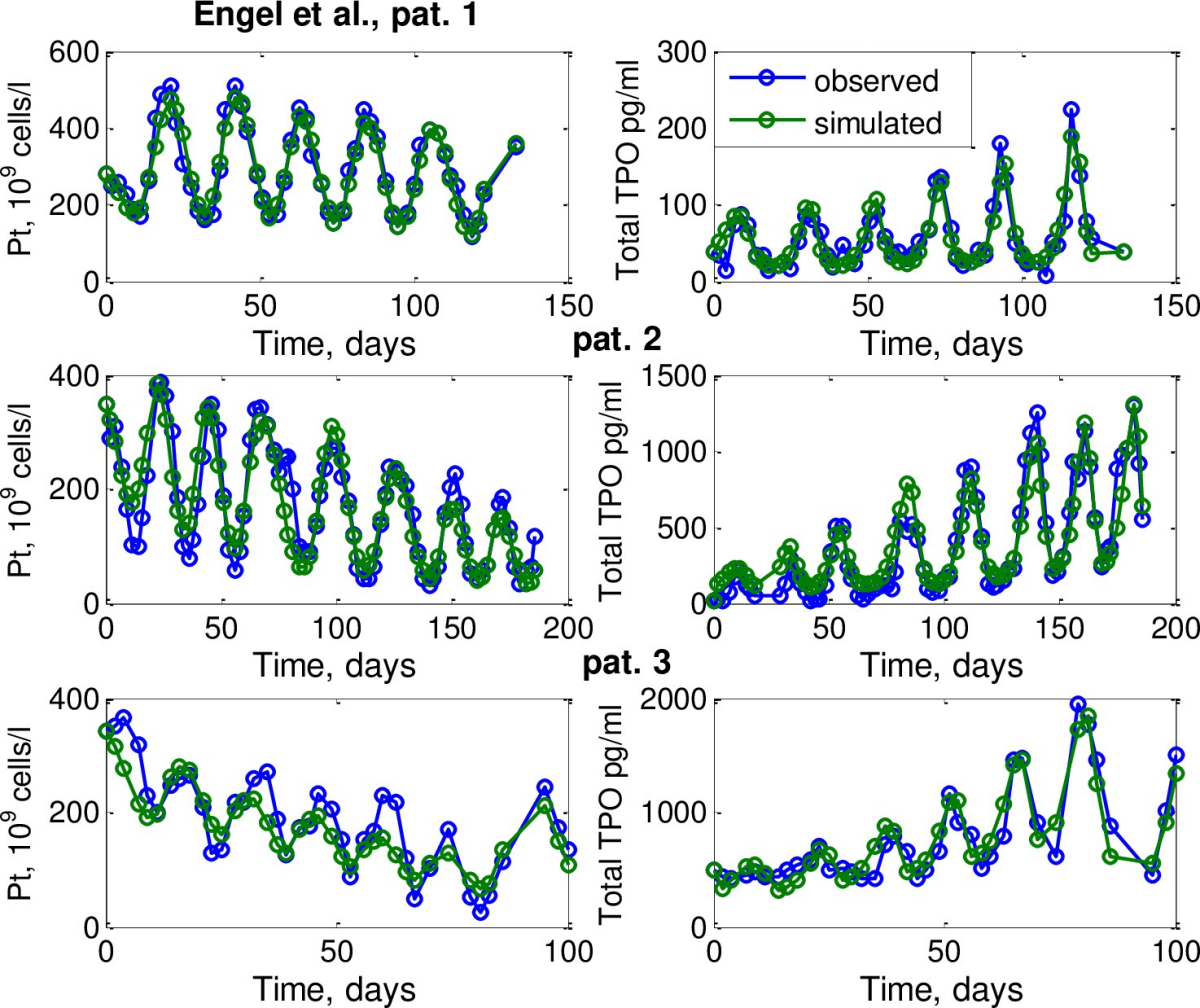
Fitting of platelets and TPO dynamics of three patients from Engel et al study Engel et al study with our newly developed biomathematical model of thrombopoiesis. Patient 1 treated with CHOEP-21, patient 2 treated with BEACOPP-21, patient treated with BEACOPP-14.

Here, we present the agreement of our model with biological data and the individual patient data of Engel et al. First, we consider the three patients studied in Engel et al. Comparisons of model and data can be found in Fig 3. We observed a good agreement of the model with all individual time series of TPO and platelets. In particular, the long-term platelet decrease accompanied with stronger long-term TPO increase could be explained by the model.

**Fig 4:**
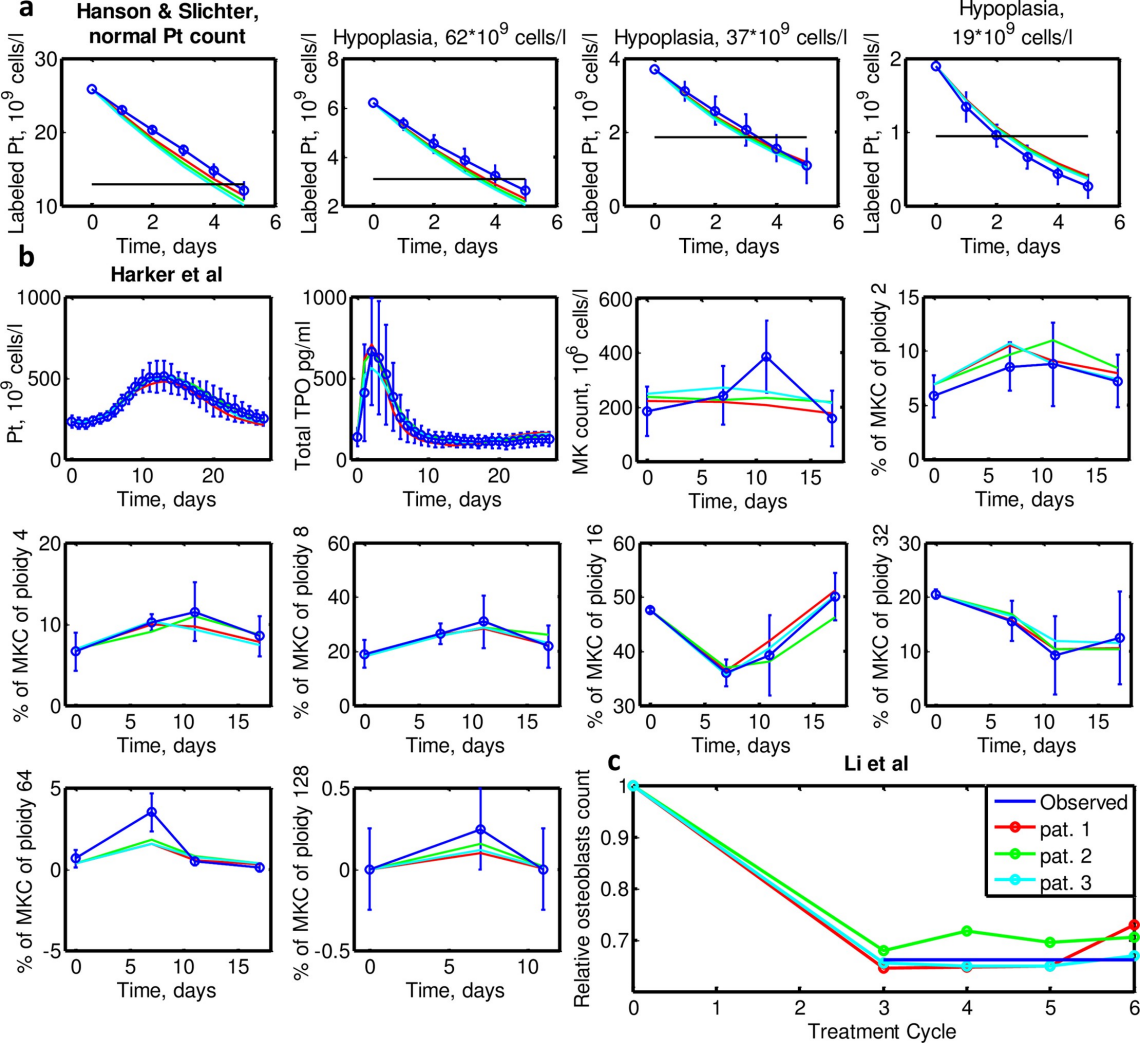
Simulations of the virtual participation of the three patients from Engel et al in other studies **a.** Dynamics of labeled platelets transfused to patients with different initial platelets counts from Hanson and Slichter study. The black horizontal line shows the half of the initial labeled platelet counts in Hanson et al. **b.** Dynamics of TPO, platelets, total MK count as well as of MKC fractions of different ploidies after peg-TPO injection at day 0 from Harker et al study. **c.** Relative osteoblasts counts after 3–6 chemotherapy cycles from Li et al study.

During parameter fitting, it is assumed that these three patients virtually participated in the study of Li et al. [21], which measured relative counts of osteoblast prior to and after chemotherapy. Results for the three patients are shown in Fig 4C. All patients are in agreement with these data.

As described in the methods section, we assumed that the three patients (virtually) also took part in the studies of Hanson et al and Harker et al. Results are shown in Fig 4A and 4B, respectively. Our model successfully explains the non-exponential dynamics of platelet elimination observed in Hanson et al. As the initial platelet level becomes smaller, platelet degradation curve becomes more concave and the half-life reduced from 3.5–4.5 days to 2 days. Patient 3 was estimated to have shorter transit time of platelets than normal patients, which explains the rapid chemotherapy-induced oscillations.

In summary, all three individual parameter sets resulted in a good fit of the dynamics of platelets count, total TPO and MKC ploidies for most of the data points from Harker et al study. Only counts of MKC ploidy 64 are slightly underestimated at day 7 and the total count of MKC is slightly underestimated at day 11. However, since standard errors are large, almost all simulations are within the 95% confidence interval of the average. Table 4 in S15 Appendix shows a good agreement of data and the simulated steady state distributions of MKC of different ploidies.

### Identifiability of parameters and sensitivity analysis

Most of the parameters showed good identifiability. The estimated population and individual parameters values and their relative standard errors are shown in Tables 1 and 2 in S15 Appendix. In S17 Appendix, we present parameters with poorer identifiability. Situations in which poor identifiability corresponds to correlated parameter estimates are summarized in Table 3 in S15 Appendix. We present only the strongest of these correlations having respective absolute values larger than 0.8. Procarbazine PD effect was unidentifiable, and thus, assumed to be zero.

We compared distributions of the individual parameter estimates of NHL-B with the corresponding priors derived from Engel et al. No significant differences were detected (see S18 Appendix).

### Comparison of individual parameter estimates

Here we discuss the parameters assumed to be heterogeneous between patients (with IIV) and compare corresponding estimates for the three patients of Engel et al. A total of 9 parameters were assumed to express IIV. Of those, two parameters are related to chemotherapy namely *pd*_*cyclo*_ (toxicity of cyclophosphamide), and $d_{{Osteo}_{loss}}$ (elimination rate of dormant stem cells and megakaryocytes due to loss of supporting osteoblasts). *pd*_*cyclo*_ is the only individual parameter of a direct effect of chemotherapy on active precursors. Consequently, its value basically influences the depth of the nadir during the first treatment cycle. Patient 2 has the largest *pd*_*cyclo*_ value corresponding to the deepest platelet nadir at the first chemotherapy cycle.

The parameter $d_{{Osteo}_{loss}}$ determines whether dormant cells are preferentially mobilized to the respective active compartments (small values) or die (large values) when supporting osteoblasts are eliminated by chemotherapy. Activation of dormant stem cells and MKC contribute to the delayed peak of platelets about 10 days after chemotherapy application. This peak is also influenced by the strength of feedback, in particular by sensitivity parameter *b*_*S*_*act*_ (self-renewal probability of active stem cells) for which we also assume IIV.

All patients have small *b*_*S*_*act*_ values indicating weak feedback on self-renewal of stem cells resulting for example in the absence of the predicted increase in total megakaryocytes counts during days 0–17 after a hypothetical stimulation with pegylated TPO (see agreement with Harker data). However these average data have large standard deviation limiting their informative value.

Patient 1 has the largest value of *b*_*S*_*act*_ as well as the smallest value of $d_{{Osteo}_{loss}}$ resulting in the largest platelet peaks during recovery. Patient 3 has low recovery peaks due to the estimated strong osteoblast reduction (large $d_{{Osteo}_{loss}}$), large PD effect *pd*_*cyclo*_ and smallest value for *b*_*S*_*act*_.

The above considerations show that a complex interplay between parameters *pd*_*cyclo*_, *b*_*S*_*act*_ and $d_{{Osteo}_{loss}}$ determines the depth of platelet nadirs, dynamics of platelet recoveries and severity of cumulative toxicity during multicycle therapy.

### Explanation of individual data from the NHL-B study

We simulated 135 patients of the NHL-B study separately, considering individual therapy adaptations such as dose reduction, therapy postponement or application of platelet concentrates. Likewise, we determined individual parameter estimates of the subset of parameters for which we assume patient heterogeneity. Ten parameters were assumed to show IIV for this patient population. All population-based parameters determined in the previous fitting steps were kept constant.

**Fig 5:**
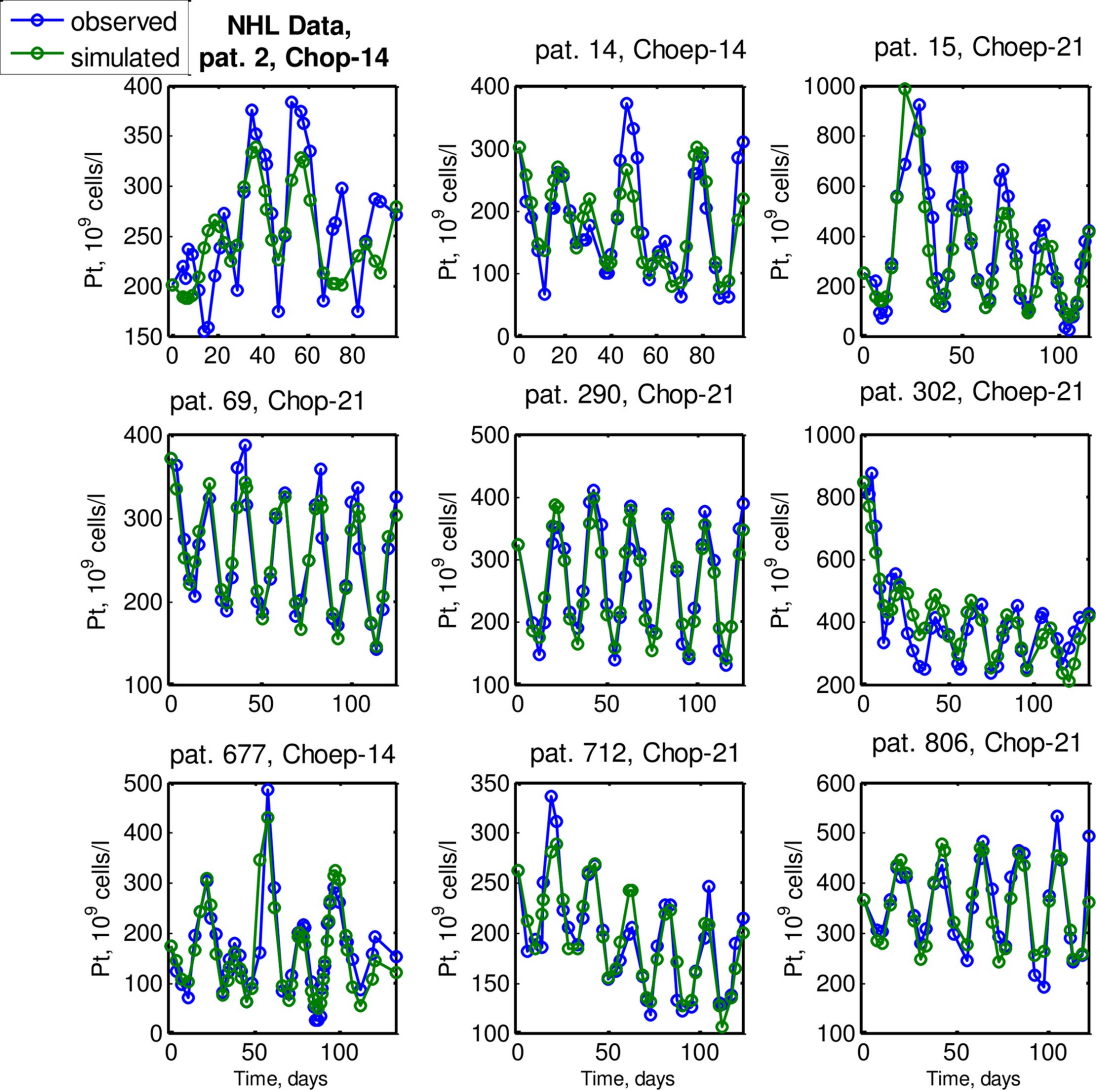
Individual model and data comparisons for nine patients from NHL-B study [1] receiving either CHO(E)P-14 or CHO(E)P-21. Strong inter-individual and inter-cycle variability of responses are observed. The model is in good agreement with the data for almost all time points.

In Fig 5 we show the agreement of model and data for 9 selected individuals of the 135 patients. All other patients are presented in figures of S16 Appendix. We obtained a good agreement for most of the patients. The individual parameter estimates are presented in the Table 1 in S16 Appendix, their relative standard errors as well as residual errors are shown in the Table 2 in S16 Appendix. No general overfitting was observed.

We observed inferior fitting results for a few patients with small initial toxicity (e.g. patient numbers 2, 52 and 313). In these cases, platelet dynamics were often noisy, i.e. similar to dynamics of healthy untreated patients from Schulthess et al study [44]. In two cases, unexpected jumps in platelet dynamics spoiled the fits (patients 1693, 1696). Errors within clinical records such as missing information regarding platelet transfusions cannot be excluded in these cases.

**Fig 6:**
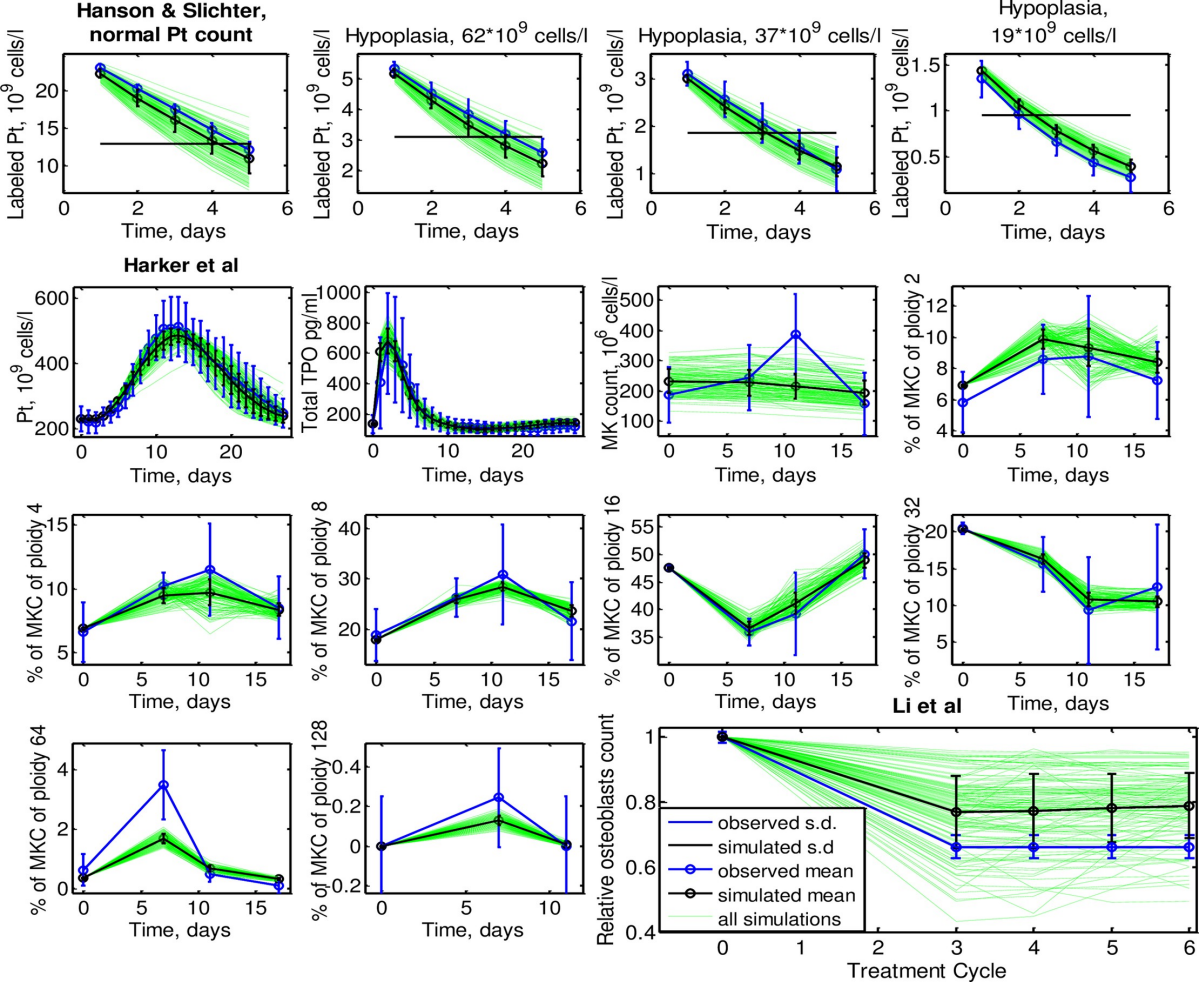
Distribution of virtual simulations (green) of 135 selected patients from NHL-B trial. **a.** Dynamics of labeled platelets transfused to patients with different initial platelets counts from Hanson and Slichter study. The black horizontal line shows the half of the initial labeled platelet counts in Hanson et al. **b.** Dynamics of TPO, platelets, total MK count as well as of MKC fractions of different ploidies after peg-TPO injection at day 0 from Harker et al study. **c.** Relative osteoblasts counts after 3–6 chemotherapy cycles from Li et al study.

In Fig 6A, 6B and 6C we show how the resulting 135 individual parameter sets reproduce the averaged data from Hanson et al, Harker et al and Li et al studies with a precision similar to that of the three patients of Engel et al. Standard deviations of the simulated biological data are often smaller than those of the observed data except for relative osteoblasts from Li et al study, whose respective virtual fits are more variable than the observed ones.

### Model application: Simulation of possible therapy adaptations of the sixth chemotherapy cycle of CHOP-like chemotherapies and prediction of long-term follow-up

**Fig 7:**
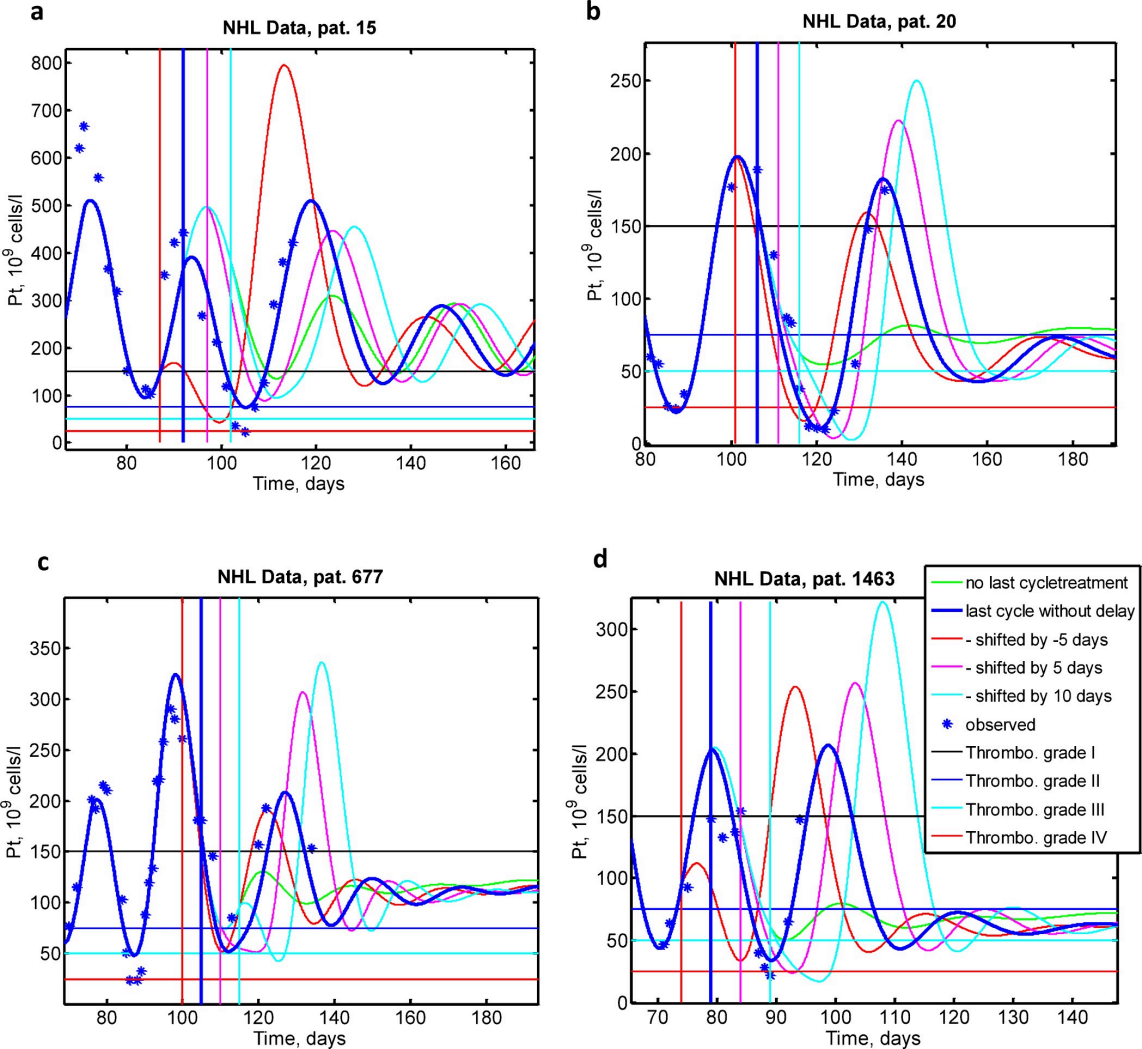
Simulation of four different scenarios for the timing of the last chemotherapy cycle for four different patients of NHL-B. We simulated omission of last cycle (green), application per protocol (dick blue), earlier application (red), 5 day postponement (magenta) and 10 day postponement (cyan). Omission of last cycle always results in higher nadirs of subsequent oscillations. **a.** Patient 15 treated with CHOEP-21. Earlier application of last cycle results in much stronger thrombopoenia; **b.** Patient 20 treated with CHOEP-21. Here, thrombopoenia increases with therapy postponement **c.** Patient 677 treated with CHOEP-14. The patient shows low sensitivity regarding postponement of therapy **d.** CHOP-14 treated patient 1463. Earlier start of the next therapy cycle does not influence nadir while treatment postponement increases thrombopenia from grade III to IV.

We used our model to simulate possible effects of a shift of the last chemotherapy cycle for patients, who developed significant thrombocytopenia at the late stages of the treatment. Fig 7A, 7B, 7C and 7D show platelet dynamics corresponding to patients 15, 20, 677 and 1463 respectively. For all patients we simulated treatment shifts of -5 (an earlier start of the last cycle), 0, 5, 10 days postponement, and finally, omission of the last cycle with subsequent tree-months follow up. All simulations show that it takes nearly two-four months after the last chemotherapy application to damp platelet oscillations sufficiently so that sever thrombopenia grades 3 or 4 is no longer observed. Interestingly, therapy postponement or earlier starts did not always result in ameliorated thrombopenia compared to the original schedule (patients 20, 677 and 1463). Sensitivity of patients regarding change of treatment schedule is predicted to be highly variable from almost no effect (patient 677) to strong nadir differences and respective thrombopenia grades (patients 15, 20 and 1463).

## Discussion

Dose-intense cytotoxic chemotherapies improved the outcome of several cancer entities [2,5,6] but is limited by the general toxicity of the drugs. However, this toxic response is highly heterogeneous between patients so that general therapy constrains are caused by a possibly small subset of patients with high risk. It is a major goal of precision medicine to identify these patients early and to introduce chemotherapeutic regimen adapted to individual risks. Here we study thrombocytopenia in the context of the treatment of aggressive non-Hodgkin’s lymphoma where it is frequently dose-limiting [1,3]. Current statistical risk models [1,3] have low precision since even in the lowest risk groups a significant amount of patients develop high toxicity, i.e. the statistical model cannot unambiguously identify the group of patients at high risk. Therefore, there is an urgent need to develop individualized mechanistic models of thrombocytopenia, which not only can explain long-term effects of multi-cyclic poly-chemotherapy but can also update their predictions based on available patient data. This is a major requirement for establishing model-based individual treatment adaptations such as individualized supportive treatments by platelet transfusions or growth factor applications or by postponement or dose-reduction of chemotherapy.

Several other complex mechanistic and semi-mechanistic models of animal and human thrombopoiesis were proposed in the past [4,24,25,45–50]. So far, only a few of them include chemotherapy applications [4,47,48] and only one of them assumes inter-individual variability of parameters [47]. This model includes biologically well-established TPO-mediated feedbacks, although without detailed description of dynamics of megakaryocytes of different ploidies. Moreover, this model does not consider dynamics of the early precursors (blast and stem cells) nor long-range effects of multi-cyclic chemotherapy.

A major goal of our model development is to allow model-based predictions regarding individual therapy adaptations. To serve similar purposes in the case of granulopoiesis, a model-based dose adaptation tool has been proposed earlier [51,52]. This tool is based on a simplistic pharmacodynamical hematopoiesis model of Friberg and Karlsson [42,43] assuming inter-individual variability of parameters. This model was applied to different hematopoietic cell lines subjected to chemotherapy including thrombopoiesis [49,50]. The model of Friberg and Karlsson assumes one proliferation compartment vulnerable to chemotherapy, three equal transit compartments and one circulating compartment, imposing a single negative feedback on the first compartment. This simplicity enables straightforward clinical data fitting for different hematopoiesis processes under chemotherapies. On the other hand, the oversimplification could obscure the connection of individual model parameters with underlying biological mechanisms such as TPO action on dynamics of maturating components CM and MKC. The model ignores the fact that chemotherapy affects all replicating compartments. Known biological feedback mechanisms of thrombopoiesis are not considered [32]. Moreover, the model is typically applied to single clinical data sets without validation of the parameter settings on the basis of other data. By our model proposal, we aim at improving this situation by building our model on biologically plausible assumptions and for several clinical and biological data sets in parallel.

To develop a reliable, comprehensive and individualized mechanistic model of thrombopoiesis under multi-cyclic poly-chemotherapy, we comprehensively revised our former model [4]. We introduced new model compartments and feedbacks in order to describe features not covered previously. The data on dynamics of megakaryocyte ploidy grades during TPO stimulation [30] allowed us to model complex parallel maturation of platelets in a much more mechanistic way as has been done so far [25]. We introduced a dormant stem cells compartment established earlier by agent-based models [15,16] but modelled here in a simpler ODE form. We modelled osteoblast support of dormant stem cells and dormant megakaryocytes. For this purpose, we integrated a mathematical model proposed by Komarova et al [20] describing the interaction of osteoblasts and osteoclasts. Chemotherapy effects were added to this model. This also allowed us to describe osteoblast dynamics during multi-cyclic chemotherapy measured by Li et al. [21]. Combined with an indirect elimination of dormant stem cells and MKCs due to lack of support, this gives a mechanistic explanation of frequently observed cumulative or late time toxicity which is not covered by statistical risk models [3]. We also integrated a non- exponential model of platelet degradation proposed by Hersh et al. [38] and revised it here.

We included a number of new biological evidence and considered additional experimental data to improve our model. For this purpose, we established and applied an innovative way of parameter fitting for our individualized model. At this, available population data and individual time courses are combined by a Bayesian approach assuming that an individual virtually participated in all experiments for which population data are available. This kind of simultaneous consideration of individual and biological (prior) data exploits all available information in a more reliable and complete way than separate estimations of groups of parameters on limited data sets. A caveat of this approach is, however, that one needs to assume comparability of patient collectives across different studies. Our approach is novel in the field since most of PK/PD modelers do not use prior information from other studies but fit exclusively clinical data of interest [42,49,50]. Heterogeneity is then addressed by mixed effects modeling [53] where parameters estimation is based on likelihood maximization for the entire population. In this case, assessment of algorithm’s convergence and overfitting are controlled exclusively for population parameters determining the distributions of individual parameters. Consequently, mixed effects modeling derives individual parameter estimates as a by-product implying high probability of insufficient fitting quality for a significant number of subjects. Moreover, pre-assumptions on the parameter distributions could spoil individual fits as well. This limits the usefulness of these models to develop individualized therapies. In contrast, our approach maximizes individual fitting precision without making any pre-assumptions on the underlying parameter distributions. We controlled convergence of fitting algorithm and reported standard errors on the individual level. We believe that such an individualized control of goodness of fit is much more appropriate for the purpose of individualized treatment management.

Our model has 102 parameters in total of which only 31 are estimated. Four parameters were directly be taken from the Komarova model [20], 28 are fixed and 28 reduced through parsimony assumptions, as described in the Table 4 of the S2 Appendix. We took 11 PK parameters as well as structural assumptions for PK models of etoposide, cyclophosphamide, doxorubicin and procarbazine from other studies [57,58] as described in S3 Appendix and Table 7 of S2 Appendix. Most of the fixed or reduced parameters correspond to chemotherapy effects or behavior of megakaryocytes of different ploidies. This can be explained by the lack of detailed data, e.g. regarding relative cytotoxic contribution of the chemotherapy drugs applied. To improve this situation, separate as well as joint individualized in-vitro studies of cytotoxic effects of cyclophosphamide, doxorubicine, etoposide and procarbazine would be helpful such as those proposed in Zeuner et al [8]. For the sake of parsimony, we did not consider potential chemotherapy drug interactions. Such interactions are not uncommon (e.g. carboplatin and paclitaxel [54]). However, for the drugs considered in the present study, no interactions could be detected based on the available data. Drug combinations of different doses of cyclophosphamide and doxorubicine would be required to unravel any interaction effects.

Moreover, for deeper understanding of the TPO-mediated regulation of megakaryocytes, one would require observations of megakaryocytes in analogy to Zeuner et al [8,22–24]. A caveat is, however, that this group studied cord blood (CB) megakaryocytes which differ significantly from bone marrow megakaryocytes by much less ploidy [24] and by their ability to frequently undergo mitoses in polyploidy state [22]. These facts leave open the question to which extend CB megakaryocytes observations could be applied to the modeling of BM megakaryocytes.

Another open question is to which extent thrombopoiesis in young healthy subjects as studied in Harker et al [30] can be compared to that of elderly patients with aggressive non-Hodgkin’s lymphoma studied in NHL-B study [1,5,6]. It is conceivable, for example, that the lack of MKC peak at day 11 after TPO injection observed in the simulations of patients from Engel et al and NHL-B (Figs 4B and 6B) can be attributed to reduced TPO responsiveness of elderly patients compared to young healthy subjects. This issue can only be resolved by studying TPO and megakaryocyte dynamics in chemotherapy-treated elderly patients, which, however, is problematic from the ethical point of view.

To derive precise estimates of individual parameters, closely meshed time series data of patients under therapy are required including full information regarding therapeutic interventions (dosing and timing of cytotoxic drugs, application of platelet concentrates or growth factors). Our major resource of individual therapy data, the NHL-B study, only partly fulfils this requirement since from the 1600 available patients only 8.4% met our inclusion criteria regarding data quality (participation in at least 4 chemotherapy cycles with at least five measurements per cycles). Moreover, only numbers but not exact time points of platelet transfusions within cycles were documented. For our modelling purpose, we assumed that transfusions occurred immediately after the smallest value and prior to an abrupt platelet increase.

To evaluate the quality of our data fitting procedure, we compared the residual errors of subjects with natural fluctuations measured in healthy subjects [44]. Residual errors appeared to be about twice the natural fluctuations implying existence of some effects unexplained by our model. Especially individual platelet dynamics with high irregularity despite of regular (cyclic) treatment are difficult to explain by our model. Irregular time series are more often observed in the 14 day regimen compared to the 21 day regimen suggesting a possible interaction with G-CSF treatment. Indeed, G-CSF can induce thrombocytopenia in healthy patients [55]. On the other hand, it is well known that G-CSF stimulates stem cells to start differentiation to blast cells [56], which are precursors for both thrombocytes and granulocytes. Thus, G-CSF has both, stimulatory and inhibitory effects on thrombopoiesis which might be relevant to explain irregular behavior. Other reasons of inferior agreement of model and data could be bleeding events, platelet consumption by infection episodes or platelet destruction by additional medications. However, in general our model predictions fitted well to the population based data and to the vast majority of individual patient data, also covering long range effects of chemotherapy.

As a possible field of clinical application, our simulations showed a strong impact of shifting the start of the next treatment cycle on the resulting thrombocytopenia. This demonstrates the importance of individual next-cycle management for chemotherapy treated patients. We currently develop a medical tool supporting dosing and timing adaptations of chemotherapies in dependence on the individual therapy response. A prototype can be found elsewhere (https://www.health-atlas.de/shiny-public/apps/thrombopenia/). It revealed that two cycles are sufficient to derive individual parameter estimates for 21-day schedules while three cycles are required for 14-day schedules.

In summary, we propose a mechanistic thrombopoiesis model of unprecedented comprehensiveness in both, biological mechanisms considered and experimental data sets explained. Our innovative method of parameter estimation allows robust determinations of individual parameter settings facilitating the development of individual treatment adaptations in the course of cytotoxic chemotherapy as an ultimate goal of systems-medicine.

## Supporting information

S1 Appendix A structure of the former model. (DOCX)

S2 Appendix Model parameters. (DOCX)

S3 Appendix Modelling chemotherapy (PK/PD modelling). (DOCX)

S4 Appendix Parsimony assumptions. (DOCX)

S5 Appendix Amplification splitting. (DOCX)

S6 Appendix Properties of the modified Z-function. (DOCX)

S7 Appendix Transit times and amplification of CM sub-compartments. (DOCX)

S8 Appendix TPO-dependent probabilities for MKC either to form proplatelets or to undergo additional endomitosis. (DOCX)

S9 Appendix Comparison of total TPO consumption by CM, megakaryocytes, proplatelets and platelets. (DOCX)

S10 Appendix Estimation of steady state and initial values from initially measured platelet counts. (DOCX)

S11 Appendix Transition of aged platelets from spleen to circulation. (DOCX)

S12 Appendix Labeled platelets. (DOCX)

S13 Appendix Log-likelihood construction. (DOCX)

S14 Appendix Numerical approach for parameter estimation. (DOCX)

S15 Appendix Parameters estimates obtained for Engel et al data. (DOCX)

S16 Appendix Parameter estimates obtained by fitting the data of the NHL-B study. (DOCX)

S17 Appendix Remarks regarding badly identifiable parameters during the fitting of Engel et al data and averaged biological data. (DOCX)

S18 Appendix Comparison of individual parameter estimates for the NHL-B study with those derived from Engel et al. data. (DOCX)
