# Notification of Republication: Modeling individual time courses of thrombopoiesis during multi-cyclic chemotherapy

**DOI:** 10.1371/journal.pcbi.1007110

**Published:** 2019-06-04

**Authors:** The PLOS Computational Biology Staff

The online version was republished to correct an equation that was typeset incorrectly. The PDF version was correct and remains unchanged. The publisher apologizes for the error.
