# Supplementary material for: Modeling individual time courses of thrombopoiesis during multi-cyclic chemotherapy
Source: PLoS Comput Biol. 2019 Mar 6;15(3):e1006775. doi: 10.1371/journal.pcbi.1006775 (PMC6422316; doi:10.1371/journal.pcbi.1006775)
Supplement: S1 Appendix — (DOCX) [file pcbi.1006775.s001.docx]

# **S1 Appendix. A structure of the former model**

Figure S1: Basic structure of the former human cell-kinetic thrombopoiesis model (according to [1]). Modelled compartments, cell fluxes and actions between them are described.


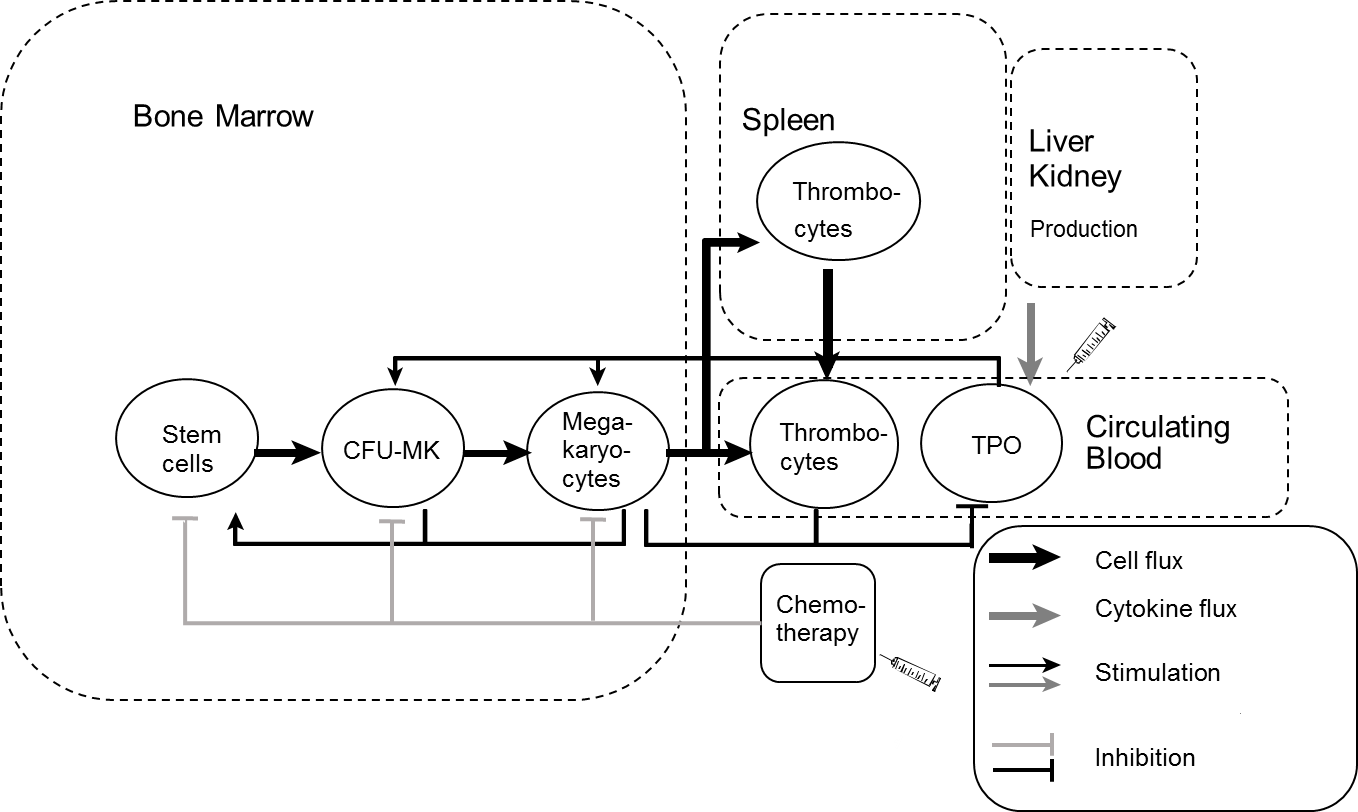


# References

1. Scholz, Markus; Gross, Arnd; Loeffler, Markus (2010): A biomathematical model of human thrombopoiesis under chemotherapy. In *Journal of theoretical biology* 264 (2), pp. 287–300. DOI: 10.1016/j.jtbi.2009.12.032
