# Supplementary material for: Modeling individual time courses of thrombopoiesis during multi-cyclic chemotherapy
Source: PLoS Comput Biol. 2019 Mar 6;15(3):e1006775. doi: 10.1371/journal.pcbi.1006775 (PMC6422316; doi:10.1371/journal.pcbi.1006775)
Supplement: S2 Appendix — (DOCX) [file pcbi.1006775.s002.docx]

# **S2 Appendix. Model parameters**

## In this section, we provide all model parameters, their description, units, values and sources. “Fitted” implies that the corresponding parameter value was determined by optimizing the agreement of model simulations and available data. “Set” means that the value is set to a biologically plausible value.

Table 1. Parameters of the sub-model of the interaction between osteoblasts and osteoclasts (adopted from Komarova et al.). In case of inter- individual variability, averages and standard deviation (in parenthesis) of parameter estimates for Engel et al data are presented.

| Parameter | Unit | Value | Source | Description |
| --- | --- | --- | --- | --- |
| *α_1_* | cells ·day^-1^ | 3 | [1] | Osteoclasts cell production |
| *α_2_* | day^-1^ | 4 |  | Osteoblasts cell production |
| *β_1_* | day^-1^ | 0.2 |  | Osteoclasts cell removal |
| *β_2_* | day^-1^ | 0.02 |  | Osteoblasts cell removal |

Table 2. Parameters of the stem cell compartment.

| Parameter | Unit | Value | Source | Description |
| --- | --- | --- | --- | --- |
| *T_cycle_* | h | 24 | [2,3] | Cycle duration of active stem cells |
| *k_dorm,nor_* | h^-1^ | 0.342 | Fitted | Normal (steady state) rate of transition of actively proliferating stem cells to dormancy |
| *k_act_* | h^-1^ | Calculated from ${kr}_{dorm,act}$, *k_dorm,nor_* and the relation $k_{act}=\frac{k_{dorm,nor}}{{kr}_{dorm,act}}$. The fitted value of *kr_dorm,act_* is 182. | | Activation rate of dormant stem cells |
| $b_{S\_act}$ | - | 0.379 (0.229) | Fitted | Steepness of the regulation function of self-renewal probability p |
| $p_{\delta}$ | - | 0.4 | Set | Difference between maximal and normal as well as between normal and minimal self-renewal probability of stem cells |

Table 3. Parameters of the compartment CM.

| Parameter | Unit | Value | Source | Description |
| --- | --- | --- | --- | --- |
| *n_CM_^e^* | - | 1 | Set | Number of TPO-dependent CM sub-compartments |
| *n_CM_^l^* | - | 5 | Heuristic guess according to [4] and [5] | Number of late TPO-independent CM sub-compartments |
| *n_CM_* | - | *n_CM_^e^+ n_CM_^l^* | Calculated | Number of all CM sub-compartments |
| *n_CM_^min^* | - | 0 | Set | Minimum number of cell cycles in the early TPO- dependent stage of CM |
| *n_CM_^nor^-n_CM_^min^* | - | 1 | Set | Difference between normal and minimal number of cell cycles in the early TPO- dependent stage of CM |
| *n_CM_^max^-n_CM_^nor^* | - | 5 | Set | Difference between maximal and normal number of cell cycles in the early TPO- dependent stage of CM |
| $n_{CM}^{unreg}$ | - | 7.87 (2.61) | Fitted | Total number of cell divisions in the late TPO-unregulated CM sub-compartments |
| $b_{A_{CM}}$ | - | 0.618 | Fitted | Steepness of the regulation function of total amplification of CM (*A_CM_*) |
| *Lim_sig_* | - | 7 | Set | Limiting parameter of the steepness of Z-function. |

Table 4. Parameters of the compartment MKC.

| Parameter | Unit | Value | Source | Description |
| --- | --- | --- | --- | --- |
| $p_{2^{k},1}^{min}\equiv p_{1}^{min}$*,*  k=3, 4, 5 | - | 1 | Set | Probability of transition from MKC sub-compartment of ploidies 8, 16 and 32 to the proplatelet compartment under minimum TPO stimulation |
| $p_{2^{k},1}^{nor}\equiv p_{1}^{nor}$*,*  k=3, 4, 5 | - | 0.102 | Fitted | Probability of transition from MKC sub-compartment ploidies 8, 16 and 32 to the proplatelet compartment under normal (steady-state) TPO stimulation |
| $p_{2^{k},1}^{max}\equiv p_{1}^{max}$,  k=3, 4, 5 | - | 0 | Set | Probability of transition from MKC sub-compartment of ploidies 8, 16 and 32 to the proplatelet compartment under maximum TPO stimulation |
| $p_{64,1}^{min}$ | - | 0 | Fixed | Probability of transition from MKC sub-compartment of ploidy 64 to ploidy 128 under minimum TPO stimulation |
| $b_{{MKC}_{p,1}}$ | - | 0.317 | Fitted | Steepness of the regulation function of the transition from MKC sub-compartments of ploidies 8, 16, and 32 to the proplatelet compartment |
| $b_{{MKC}_{p,64,1}}$ | - | 0.350 | Fitted | Steepness of the regulation function of the transition from MKC sub-compartment of ploidy 64 to the proplatelets compartment |
| $p_{2^{k},2}\equiv p_{2}$,  k=3, 4, 5 | - | 0.695 | Fitted | Probability of transition from MKC sub-compartment of ploidy 2^k^ to the next ploidy compartment |
| $b_{{MKC}_{p,2}}$ | - | 0 | Set | Steepness of regulation function of transition from MKC sub-compartments of ploidies 8, 16, 32 and 64 to the next ploidy compartment |
| $T_{dorm,MKC}$ | h | 12 | Set assuming that it is in the order of cell-cycle time | Transition time from active to inactive (dormant) MKC sub-compartments of the same ploidy (8, 16 and 32) |
| $k_{rev\_dorm,2^{k}}^{min}$,  k=3, 4, 5 | h^-1^ | 0 | Set | Transition rates of inactive (dormant) MKC sub-compartments of ploidy 2^k^ to the active sub-compartment of same ploidy under minimum TPO stimulation |
| $T_{rev\_dorm,2^{k}}^{nor}$ | h | 75.0, 477, 328 respectively | Fitted | Transition times of inactive (dormant) MKC sub-compartment of ploidy 2^k^ to the active sub-compartment of same ploidy under normal TPO stimulation |
| $k_{rev\_dorm,2^{k}}^{nor}$,  k=3, 4, 5, | h^-1^ | Calculated from the respective reciprocal values:  $k_{rev\_dorm,2^{k}}^{nor}=\frac{1}{T_{rev\_dorm,2^{k}}^{nor}}$, | | Transition rates of inactive (dormant) MKC sub-compartment of ploidy 2^k^ to the active sub-compartment of same ploidy under normal TPO stimulation |
| $k_{rev\_dorm,2^{k}}^{max}$,  k=3, 4, 5 | h^-1^ | 1 | Set | Transition rate from inactive (dormant) MKC sub-compartments of ploidy 2^k^ to the active sub-compartment of same ploidy under maximum TPO stimulation |
| $b_{rev\_dorm,8}$ | - | 0 | Set | Steepness of regulation function of transitions from inactive (dormant) MKC sub-compartment of ploidy 8 to the active sub-compartment of ploidy 8 under normal TPO stimulation |
| $b_{rev\_dorm,2^{k}}$*,*  k=4, 5 | - | 1.23, 2.34 respectively | Fitted | Steepness of regulation function of transitions from inactive (dormant) MKC sub-compartment of ploidy 2^k^ to the active sub-compartment of same ploidy under normal TPO stimulation |
| $T_{PP}$ | h | 3.70 | Fitted | Transit time of proplatelet compartment |

Table 5. Parameters of compartment PLC.

| Parameter | Unit | Value | Source | Description |
| --- | --- | --- | --- | --- |
| *npt_pcu_* | cells | 504 | Fitted | Produced number of platelets per ploidy of MKC |
| *p* | - | 2 | Set | Hill coefficient of platelet consumption see eq. (30) |
| *h_s_* | 10^9^ cells L^-1^ | 1.6 | Set | Value corresponding to half maximum of Michaelis-Menten kinetics of platelet uptake by vessels (Michaelis-Menten constant) |
| *k_s_* | 10^9^ cells L^-1^h^-1^ | 0.183 | Fitted | Maximum value of Michaelis- Menten kinetics of platelet uptake by vessels |
| *T_PL_* | h | 261 (14.8) | Fitted | Transit time of platelets |
| *n* | - | 7 | Set according to [6] | Number of age-compartments in PLC and PLS respectively |
| *k_circ_* | h^-1^ | 0.33 | Fixed [6] or calculated according to biological data [7] | Ratio of platelets entering directly into circulation |
| $k_{i}^{SC}$*, i=1,…,n* | h^-1^ | Calculated |  | Transition coefficient between spleen and circulating platelets of age-compartment *i* |
| *g_i_, i=1,…,n* | - |  |  | proportion of platelets of age *i* in circulation |
| *q* | - | 2/3 |  | fixed parameter used to determine *g_i_* according to (S.11.1) |
| *r_PL,0,nor_* | - | 1.04 (0.0608) | Fitted | Ratio of the initial PLC count to the steady state PLC count |

Table 6. Parameters of TPO compartment.

| Parameter | Unit | Value | Source | Description |
| --- | --- | --- | --- | --- |
| *q_TPO_* | h^-1^ | 0.0121 | Fitted | Flux rate of pegylated TPO between injection and delay compartment in case of subcutaneous injection |
| *Fr_TPO,dir_* | - | 0.347 | Fitted | Fraction of TPO entering enters the the central compartment directly due to force of injection |
| *r_TPO,nor,0_* | ng/ml | 0.994 (0.848) | Fitted | Relation of the steady state TPO value to the initial TPO value |
| *T_TPO_* | h | 23.1 | Fitted | Transition time of unspecific TPO elimination |
| *k_m,TPO_* | - | 0.264 (0.215) | Fitted | TPO saturation of specific elimination  (Michaelis-Menten constant) |
| *w_re_* | rec^-1^ h^-1^ | Calculated from $w_{re}=\frac{30\cdot{230\cdot BV\cdot10}^{9}}{\hat{w}_{re}}$ according to (S.9.2). The fitted value of $\hat{w}_{re}$ is 1.07 (0.590) | | Maximum TPO elimination rate by a single TPO receptor |
| *w_PLC_* | cells^-1^ h^-1^ | - | Calculated from *w_re_*, see (S.9.1) | Maximum TPO elimination rate by circulating platelets |
| $w_{MKC,k}$ | cells^-1^ h^-1^ | - |  | Maximum TPO elimination rate by megakaryocytes of ploidy 2^k^ |
| $w_{PP}$ | cells^-1^ h^-1^ | - |  | Maximum TPO elimination rate by proplatelets |
| $T_{Del}$ | h | 37.1 | Fitted | Transition time of delayed TPO effect |
| $T_{Del,2^{5}}$ | h | 107 | Fitted | Additional transition time of delayed TPO effect on activation of dormant MKC of ploidy 2^5^ |
| α | h^-1^ | Calculated by steady state condition  $\alpha=\frac{1}{T_{TPO}}+\frac{\left( w_{PLC}\cdot\sum_{i=1}^{n} C_{{PLC}_{i}}^{nor}+\sum_{k=1}^{7} \left( w_{MKC,k}\cdot C_{MKC,2^{k}}^{nor} \right)+w_{PP}\cdot{PP}^{nor} \right)}{\left( 1+k_{m,TPO} \right)}$. | | Endogenous production of TPO |

Table 7. Parameters of PK as well as of chemotherapy effects.

| Parameter | Unit | Value | Source | Description |
| --- | --- | --- | --- | --- |
| *CL_doxo_* | L h^-1^ | 47.6 | [8] | Clearance of doxorubicin |
| *V_1,doxo_* | L | 12.3 |  | Volume of the central compartment (doxorubicin) |
| Q_doxo_ | L h^-1^ | 60.3 |  | Intercompartmental clearance between the central and peripheral compartment (doxorubicin) |
| *V_2,doxo_* | L | 421 |  | Volume of the peripheral compartment (doxorubicin) |
| *CL_cyclo_* | L h^-1^ | 4.23 |  | Clearance of cyclophosphamide |
| *V_1,cyclo_* | L | 34.6 |  | Volume of the central compartment (cyclophosphamide) |
| *V_1,etop_* | L | 7.96 | [9] | Volume of the central compartment (etoposide) |
| *k_e,etop_* | h^-1^ | 7.03 |  | Elimination coefficient from the central compartment (etoposide) |
| *k_12,etop_* | h^-1^ | 10.46 |  | transition rate from the central to peripheral compartment (etoposide) |
| *k_21,etop_* | h^-1^ | 9.81 |  | transition rate from the peripheral to the central compartment (etoposide) |
| *V_1,proc_* | L | Equal to blood volume | Set | Volume of the central compartment (procarbazine) |
| *k_e,proc_* | h^-1^ | Log(2)/1 | [10] | Elimination coefficient from the central compartment (procarbazine) |
| $\frac{{pd}_{doxo}^{thr}}{{pd}_{cyclo}^{thr}}$ | - | 229.2 | Assumed to be equal to those of granulopoiesis. The later were calculated from ${pd}_{doxo}^{neu}$, ${pd}_{cyclo}^{neu}$ and ${pd}_{etop}^{neu}$ | Ratio of PD effects of doxorubicin and etoposide to that of cyclophosphamide for thrombopoiesis, see Table 1 in S4 Appendix |
| $\frac{{pd}_{etop}^{thr}}{{pd}_{cyclo}^{thr}}$ | - | 1.597 |  |  |
| *c_PD,Osteo_* | *-* | 3.23E-04 | Fitted | Ratio of chemotherapy toxicity on CM precursors and osteoblasts / osteoclasts |
| *pd_cyclo_* (*pd_cyclo,s_)* | L· (h·mg)^-1^ | 0.0134 (0.00357) | Fitted | Toxicity of cyclophosphamide on S compartment |
| *pd_doxo_ (pd_doxo,s_)* | L· (h·mg)^-1^ | 1 | Deduced from $\frac{{pd}_{doxo}^{thr}}{{pd}_{cyclo}^{thr}}$  and ${pd}_{cyclo}^{thr}$ (S.4) | Toxicity of doxorubicin on S compartment |
| *pd_etop_, (pd_etop,cm_)* | L· (h·mg)^-1^ | - | Deduced from $\frac{{pd}_{etop}^{thr}}{{pd}_{cyclo}^{thr}}$ and ${pd}_{cyclo}^{thr}$ (S.4) | Toxicity of etoposide on S compartment |
| *pd_procar_ (pd_procar,s_)* | L· (h·mg)^-1^ | 0 | Set | Toxicity of procarbazine on S compartment |
| *pd_CM_* | L· (h·mg)^-1^ | 1 | Set | Ratio of toxicities on CM and S compartments, valid for all drugs considered |
| *pd_MKC_* | L· (h·mg)^-1^ | 0.371 | Calculated from data [11] see (S.3.5) | Ratio of toxicities on mature MKC (ploidies 8-128) and CM, valid for all cytotoxic drugs |
| *pd_MKCimm_* | L· (h·mg)^-1^ | 0.689 |  | Ratio of toxicities on immature MKC (ploidies 2,4) and CM, valid for all cytotoxic drugs |
| *D_ψ_* | h^-1^ | 0.0197 | Fitted | Delay parameter of chemotherapy effect on osteoblasts / osteoclasts |
| $d_{{Osteo}_{loss}}$ | h^-1^ | 1.42 (0.743) | Fitted | Elimination rate of dormant cells due to lack of osteoblast support |

References

1. Komarova SV, Smith RJ, Dixon SJ, Sims SM, Wahl LM. Mathematical model predicts a critical role for osteoclast autocrine regulation in the control of bone remodeling. Bone. 2003; 33: 206–215. doi: 10.1016/S8756-3282(03)00157-1.

2. Bernard S, Herzel H. Why do cells cycle with a 24 hour period. Genome Inform. 2006; 17: 72–79.

3. Cooper GM. The cell. A molecular approach. 2nd ed. Washington, DC: ASM Press; 2000.

4. Guerriero R, Testa U, Gabbianelli M, Mattia G, Montesoro E, Macioce G, et al. Unilineage megakaryocytic proliferation and differentiation of purified hematopoietic progenitors in serum-free liquid culture. Blood. 1995; 86: 3725–3736.

5. Harker LA, Roskos LK, Marzec UM, Carter RA, Cherry JK, Sundell B, et al. Effects of megakaryocyte growth and development factor on platelet production, platelet life span, and platelet function in healthy human volunteers. Blood. 2000; 95: 2514–2522.

6. Scholz M, Gross A, Loeffler M. A biomathematical model of human thrombopoiesis under chemotherapy. J Theor Biol. 2010; 264: 287–300. doi: 10.1016/j.jtbi.2009.12.032.

7. Freedman M, Altszuler N, Karpatkin S. Presence of a nonsplenic platelet pool. Blood. 1977; 50: 419–425.

8. Crombag M-RBS, Joerger M, Thürlimann B, Schellens JHM, Beijnen JH, Huitema ADR. Pharmacokinetics of Selected Anticancer Drugs in Elderly Cancer Patients: Focus on Breast Cancer. Cancers (Basel). 2016; 8. doi: 10.3390/cancers8010006.

9. Faivre C, El Cheikh R, Barbolosi D, Barlesi F. Mathematical optimisation of the cisplatin plus etoposide combination for managing extensive-stage small-cell lung cancer patients. Br J Cancer. 2017; 116: 344–348. doi: 10.1038/bjc.2016.439.

10. Procarbazine. Available: https://cancercare.on.ca/CCO_DrugFormulary/pages/DfPdfContent.aspx?cat=DM&name=procarbazine.

11. Zeuner A, Signore M, Martinetti D, Bartucci M, Peschle C, Maria R de. Chemotherapy-induced thrombocytopenia derives from the selective death of megakaryocyte progenitors and can be rescued by stem cell factor. Cancer Res. 2007; 67: 4767–4773. doi: 10.1158/0008-5472.CAN-06-4303.
