# Supplementary material for: Modeling individual time courses of thrombopoiesis during multi-cyclic chemotherapy
Source: PLoS Comput Biol. 2019 Mar 6;15(3):e1006775. doi: 10.1371/journal.pcbi.1006775 (PMC6422316; doi:10.1371/journal.pcbi.1006775)
Supplement: S3 Appendix — (DOCX) [file pcbi.1006775.s003.docx]

# **S3 Appendix. Modelling chemotherapy (PK/PD modelling)**

In this section, we provide detailed equations of our modelling of chemotherapy effects. We attached existing PK models of the relevant drugs and inferred information on PD parameters from other studies if possible. We always assume that the central compartment is the injection compartment. The relations between PK parameters are as follows:

$\begin{matrix} k_{e}=\frac{CL}{V_{1}} \\ t_{1/2}=\frac{log\left( 2 \right)}{k_{e}} \\ \begin{matrix} k_{12}=\frac{Q}{V_{1}} \\ k_{21}=\frac{Q}{V_{2}} \end{matrix} \end{matrix}$ , (S.3.1)

where *k_e_* is the elimination coefficient from the central compartment (1/h), *V_i_* is the volume of distribution of the i-th compartment(L), *Cl* is the clearance from the central compartment (L/h), *t_1/2_* is the half-life of the drug in the central compartment, Q is an intercompartmental clearance between the central and the peripheral compartment (L/h), k_12_ is a transition rate from the central to peripheral compartment (1/h), k_21_ is a transition rate from the peripheral to the central compartment (1/h). PK parameters for the drugs are presented in Table 7 in S2 Appendix.

## **Chemotherapies: BEACOPP and CHOP-like therapies**

The half-life of cyclophosphamide is 3-12 hours [1]. The distribution half-life of doxorubicin is approximately 5 minutes, while the terminal half-life is 20 to 48 hours [2].

Population PK / PD models of cyclophosphamide and doxorubicin have been proposed in the literature [3]. According to this study, the model of cyclophosphamide has one compartment, while the model of doxorubicin has two compartments:

$\frac{d}{dt}C_{cyclo,1}=-k_{e,cyclo}\cdot C_{cyclo,1}$,

$\begin{matrix} \frac{d}{dt}C_{doxo,1}=-k_{e,doxo}\cdot C_{doxo,1}-k_{12,doxo}\cdot C_{doxo,1}+k_{21,doxo}\cdot C_{doxo,2} \\ \frac{d}{dt}C_{doxo,2}=k_{12,doxo}\cdot C_{doxo,1}-k_{21,doxo}\cdot C_{doxo,2} \end{matrix}$. (S.3.2)

On intravenous administration, the disposition of etoposide is best described as a biphasic process with a distribution half-life of about 1.5 hours and a terminal elimination half-life ranging from 4 to 11 hours [4]. A two-compartment population PK model of etoposide was proposed by [5]:

$\begin{matrix} \frac{d}{dt}C_{etop,1}=-k_{e,etop}\cdot C_{etop,1}-k_{12,etop}\cdot C_{etop,1}+k_{21,etop}\cdot C_{etop,2} \\ \frac{d}{dt}C_{etop,2}=k_{12,etop}\cdot C_{etop,1}-k_{21,etop}\cdot C_{etop,2} \end{matrix}$. (S.3.3)

Procarbazine has much faster kinetics, compared to cyclophosphamide, doxorubicin and etoposide. Its degradation proceeds with half-life of 60 minutes [6]. We assume a one-compartment PK model with distribution volume equal to blood volume:

$\frac{d}{dt}C_{carbaz,1}=-k_{e,carbaz}\cdot C_{carbaz,1}$. (S.3.4)

## **Application of thrombocyte concentrates**

Thrombocytes were applied as concentrates. This was modelled in the following way: Since platelet development is modeled by seven age-compartments, we divided respective transfused platelet amounts into seven parts according to the steady-state distribution of circulating age compartments (see S6 Appendix for calculation of steady state values). Thus, each platelet transfusion resulted in simultaneous discontinuous jumps in the seven age-compartments of platelets in circulation.

## **Data of the NHL-B study [7,8]**

A total of 13 out of 135 selected patients from the NHL-B study received platelet transfusions. However, the documentation of these interventions is limited since neither the exact days of platelet transfusions nor the amount of applied concentrates was documented.

Personal communication with medical doctors and data managers revealed that pooled platelet concentrates have been used. Generally, 240 - 360 x 10^9^ platelets are transfused per portion [9]. Thus, we assumed 300 x 10^9^ platelets per transfusion. Since cycle day of application is not available, we assumed that in the case of a single transfusion, the application was performed directly after the lowest measured platelet count of the corresponding cycle. If a patient received k>1 transfusions in a cycle, we assumed that they were applied immediately after the k-th smallest platelet counts.

## **Hanson et al study [10]**

The transfused platelets were harvested from 500 mL of whole autologous blood and labeled with ^51^Cr . Since 500 ml of blood is approximately 1/10 of the adult blood volume, we assumed that each patient received 1/10 of his platelet count. Thus we assumed that the 4 patient groups received 25.8 x10^9^, 6.2x10^9^, 3.7x10^9^ or 1.9x10^9^ auto-transfused platelets per liter respectively.

## **Comparison of chemotherapy induced toxic effects on CM and MKC**

According to in vitro studies [11], different platelet precursors have different sensitivity to chemotherapeutic drugs. From this study we derived an average ratio of toxic effects on CM and MKC as follows. Zeuner et al examined 3 drugs: cytosine arabinoside, vincristine and cisplatin. Fig 1 B from this study showed apoptosis fraction of control (untreated cells) as well as of CD34+, MKC blasts, Pro MKC, immature MKC and mature MKC. This classification is slightly different from our modelled cell compartments because the authors distinguish CD34+ from blast cells. After subtracting the apoptotic fraction of controls, we performed a linear regression between apoptotic fractions of mature MKC (ploidy 8-128), immature MKC (ploidies 2, 4) and blasts. This resulted in the approximation

$\begin{matrix} {pdr}_{MKC}=0.371\cdot{pdr}_{CM} \\ {pdr}_{MKCimm}=0.689\cdot{pdr}_{CM} \end{matrix}$. (S.3.5)

References

1. Boddy AV, Yule SM. Metabolism and pharmacokinetics of oxazaphosphorines. Clin Pharmacokinet. 2000; 38: 291–304. doi: 10.2165/00003088-200038040-00001.

2. Doxorubicin. Available: http://www.drugs.com/pro/doxorubicin.html.

3. Crombag M-RBS, Joerger M, Thürlimann B, Schellens JHM, Beijnen JH, Huitema ADR. Pharmacokinetics of Selected Anticancer Drugs in Elderly Cancer Patients: Focus on Breast Cancer. Cancers (Basel). 2016; 8. doi: 10.3390/cancers8010006.

4. Etoposide. Available: http://www.drugs.com/pro/etoposide.html.

5. Faivre C, El Cheikh R, Barbolosi D, Barlesi F. Mathematical optimisation of the cisplatin plus etoposide combination for managing extensive-stage small-cell lung cancer patients. Br J Cancer. 2017; 116: 344–348. doi: 10.1038/bjc.2016.439.

6. Procarbazine. Available: https://cancercare.on.ca/CCO_DrugFormulary/pages/DfPdfContent.aspx?cat=DM&name=procarbazine.

7. Pfreundschuh M, Trumper L, Kloess M, Schmits R, Feller AC, Rube C, et al. Two-weekly or 3-weekly CHOP chemotherapy with or without etoposide for the treatment of elderly patients with aggressive lymphomas: results of the NHL-B2 trial of the DSHNHL. Blood. 2004; 104: 634–641. doi: 10.1182/blood-2003-06-2095.

8. Pfreundschuh M, Trumper L, Kloess M, Schmits R, Feller AC, Rudolph C, et al. Two-weekly or 3-weekly CHOP chemotherapy with or without etoposide for the treatment of young patients with good-prognosis (normal LDH) aggressive lymphomas: results of the NHL-B1 trial of the DSHNHL. Blood. 2004; 104: 626–633. doi: 10.1182/blood-2003-06-2094.

9. Seifried E, Klueter H, Weidmann C, Staudenmaier T, Schrezenmeier H, Henschler R, et al. How much blood is needed. Vox Sang. 2011; 100: 10–21. doi: 10.1111/j.1423-0410.2010.01446.x.

10. Hanson SR, Slichter SJ. Platelet kinetics in patients with bone marrow hypoplasia: evidence for a fixed platelet requirement. Blood. 1985; 66: 1105–1109.

11. Zeuner A, Signore M, Martinetti D, Bartucci M, Peschle C, Maria R de. Chemotherapy-induced thrombocytopenia derives from the selective death of megakaryocyte progenitors and can be rescued by stem cell factor. Cancer Res. 2007; 67: 4767–4773. doi: 10.1158/0008-5472.CAN-06-4303.
