# Supplementary material for: Modeling individual time courses of thrombopoiesis during multi-cyclic chemotherapy
Source: PLoS Comput Biol. 2019 Mar 6;15(3):e1006775. doi: 10.1371/journal.pcbi.1006775 (PMC6422316; doi:10.1371/journal.pcbi.1006775)
Supplement: S4 Appendix — (DOCX) [file pcbi.1006775.s004.docx]

# **S4 Appendix. Parsimony assumptions**

A number of parsimony assumptions were tested during estimation of our model parameters to avoid over-fitting. We present and discuss these assumptions in the following in detail.

## **Parsimony of chemotherapy model parameters**

The system (1) contains 20 toxicity parameters whose identification is prone to overfitting. In order to decrease the number of free parameters, we introduce four basic pharmacodynamic (PD) parameters *pd_cyclo_*, *pd_doxo_*, *pd_etop_*, *pd_procar_*. We assume that these are the PD effects on active proliferating stem cells, i.e.

${pd}_{procar,S}={pd}_{procar}$, ${pd}_{doxo,S}={pd}_{doxo}$, ${pd}_{cyclo,S}={pd}_{cyclo}$, ${pd}_{etop,S}={pd}_{etop}$ , (S.4.1)

We assume that the relations of PD effects between different compartments are the same for all drugs representing the chemosensitivity of different cell stages in relation to that of active stem cells:

$\begin{matrix} \begin{matrix} \frac{{pd}_{cyclo,MKCimm}}{{pd}_{cyclo,S}}=\frac{{pd}_{doxo,MKCimm}}{{pd}_{adria,S}}=\frac{{pd}_{procar,MKCimm}}{{pd}_{procar,S}}=\frac{{pd}_{etop,MKCimm}}{{pd}_{etop,S}}\equiv{pdr}_{MKCimm} \\ \frac{{pd}_{cyclo,MKC}}{{pd}_{cyclo,S}}=\frac{{pd}_{doxo,MKC}}{{pd}_{doxo,S}}=\frac{{pd}_{procar,MKC}}{{pd}_{procar,S}}=\frac{{pd}_{etop,MKC}}{{pd}_{etop,S}}\equiv{pdr}_{MKC} \end{matrix} \\ \frac{{pd}_{cyclo,CM}}{{pd}_{cyclo,S}}=\frac{{pd}_{doxo,CM}}{{pd}_{doxo,S}}=\frac{{pd}_{procar,CM}}{{pd}_{procar,S}}=\frac{{pd}_{etop,CM}}{{pd}_{etop,S}}\equiv{pdr}_{CM} \end{matrix}$, (S.4.2)

Due to poor identifiability we assume equal PD effects for all drugs on active stem cells and CM cells:

${pdr}_{CM}=1$, (S.4.3)

In the schedules considered, cyclophosphamide was always applied in combination with doxorubicin, and in most cases, also with etoposide. Thus, *pd_cyclo_* and *pd_doxo_* can hardly be estimated independently. However, population PD effects of cyclophosphamide, doxorubicin and etoposide on neutrophils were estimated in a few studies using the simplistic pharmacodynamical haematopoiesis model of Friberg and Karlsson [1]. We adopted the linear slope variants of PD effects for etoposide [1], cyclophosphamide and doxorubicin [2]. These semi-mechanistic models assume that chemotherapy affects only the proliferating compartment as follows:

$\frac{dProl}{dt}= Feed\left( Circ \right)\cdot\left( 1-{pd}_{X}\cdot X \right)\cdot Prol$, (S.4.4)

where *Feed(Circ)* is a feedback coefficient dependent on the circulating cells *Circ*, *Prol* is a number of cells in proliferating compartment, *X* is the concentration of the drug and *pd_X_* is a respective PD (“slope”) coefficient.

It is necessary to remember that drug-induced thrombopoenia and neutropoenia both depending considerably on the elimination of common progenitor cells (stem cells and early blast cells). For simplicity, we assume the same ratios of drug toxicities on neutropoiesis as observed for thrombopoiesis, i.e.

$\begin{matrix} \frac{{pd}_{doxo}^{thr}}{{pd}_{cyclo}^{thr}}=\frac{{pd}_{doxo}^{neu}}{{pd}_{cyclo}^{neu}} \\ \frac{{pd}_{etop}^{thr}}{{pd}_{cyclo}^{thr}}=\frac{{pd}_{etop}^{neu}}{{pd}_{cyclo}^{neu}} \end{matrix}$ , (S.4. 5)

We translated all PD coefficients from μM^-1^ unit (used in the respective publications) to (mg/L)^-1^. Parameter estimates are presented in Table 1:

Table 1. PD effects of etoposide, cyclophosphamide and doxorubicin relative to neutropenia. ^*^ [1], ^**^ [2] .

| Drug | Molecular weight, g/mol | ${pd}_{drug}^{neu}$ , μM^-1^ | ${pd}_{drug}^{neu}$ , (mg/L)^-1^ | $\frac{{pd}_{drug}^{neu}}{{pd}_{cyclo}^{neu}}$ |
| --- | --- | --- | --- | --- |
| Etoposide | 589 | 0.126 ^*^ | 0.214 | 1.597 |
| Cyclophosphamide | 261 | 0.035^**^ | 0.134 | 1 |
| Doxorubicin | 544 | 16.7^**^ | 30.7 | 229.2 |

As discussed in S3 Appendix, we derived a relation between *pdr_CM_* and *pdr_MKC_* (S.3.5) using in vitro studies [3].

Due to lack of other information, we assumed that the toxicity function of CM is the same as that determining long-range accumulating toxicity effects of osteoblasts: $\Psi_{Osteo}=\Psi_{CM}$. This is motivated by the assumption that proliferating precursors have similar chemosensitivity. Equation (4) in the paper shows how the loss in osteoblasts reduce bone marrow capacity of stem cells.

## **Parsimony assumptions for parameters of megakaryocytes dynamics**

We assumed that the probabilities of transition from MKC sub-compartment of ploidy 2^k^ to the corresponding sub-compartments of higher ploidy are the same for k=3, 4, 5. The corresponding probabilities under minimum and maximum stimulations were fixed to 0 and 1, respectively. The respective probabilities under normal TPO stimulation were estimated. Similarly, we assumed that the probabilities of transition from MKC sub-compartment of ploidy 2^k^ to the proplatelet compartment are the same for k=3, 4, 5. The corresponding probabilities under normal and maximal stimulations were estimated, while the probabilities under the minimal stimulation were fixed at 1.

$\begin{matrix} p_{2^{k},1}^{min}\equiv p_{1}^{min}=1 \\ \begin{matrix} p_{2^{k},1}^{max}\equiv p_{1}^{max} \\ p_{64,1}^{min}=0 \end{matrix} \\ \begin{matrix} p_{2^{k},2}^{min}\equiv p_{2}^{min}=0 \\ p_{2^{k},2}^{max}\equiv p_{2}^{max}=1 \\ \begin{matrix} k_{rev\_dorm,2^{k}}^{min}=0 \\ \begin{matrix} k_{rev\_dorm,2^{k}}^{max}=1h^{-1} \\ p_{2^{k},1}^{nor}\equiv p_{1}^{nor} \\ \begin{matrix} p_{2^{k},2}^{nor}\equiv p_{2}^{nor} \\ \end{matrix} \end{matrix} \end{matrix} \end{matrix} \end{matrix}\begin{matrix} , & k=3, 4, 5 \end{matrix}$. (S.4.6)

References

1. Friberg LE, Henningsson A, Maas H, Nguyen L, Karlsson MO. Model of chemotherapy-induced myelosuppression with parameter consistency across drugs. J Clin Oncol. 2002; 20: 4713–4721.

2. Crombag M-RBS, Joerger M, Thürlimann B, Schellens JHM, Beijnen JH, Huitema ADR. Pharmacokinetics of Selected Anticancer Drugs in Elderly Cancer Patients: Focus on Breast Cancer. Cancers (Basel). 2016; 8. doi: 10.3390/cancers8010006.

3. Zeuner A, Signore M, Martinetti D, Bartucci M, Peschle C, Maria R de. Chemotherapy-induced thrombocytopenia derives from the selective death of megakaryocyte progenitors and can be rescued by stem cell factor. Cancer Res. 2007; 67: 4767–4773. doi: 10.1158/0008-5472.CAN-06-4303.
