# Supplementary material for: Modeling individual time courses of thrombopoiesis during multi-cyclic chemotherapy
Source: PLoS Comput Biol. 2019 Mar 6;15(3):e1006775. doi: 10.1371/journal.pcbi.1006775 (PMC6422316; doi:10.1371/journal.pcbi.1006775)
Supplement: S5 Appendix — (DOCX) [file pcbi.1006775.s005.docx]

# **S5 Appendix. Amplification splitting**

Total amplification was split into an amplification of the influx $A_{x}^{in}$ and an amplification of the efflux $A_{x}^{out}$ resulting in a more realistic (i.e. delayed) response of compartments regarding changes of the regulatory functions [1]:

$A_{X}^{in}=\left\{\begin{matrix} \frac{A_{X}-1}{ldA_{X}}\begin{matrix} & for\begin{matrix} & A_{X}\neq0,\begin{matrix} & A_{X}\neq1 \end{matrix} \end{matrix} \end{matrix} \\ \ln2\begin{matrix} & for\begin{matrix} & A_{X}=1\begin{matrix} \begin{matrix} \begin{matrix} \begin{matrix} & \end{matrix} & \end{matrix} & \end{matrix} & \end{matrix} \end{matrix} \end{matrix} \\ 0\begin{matrix} & for\begin{matrix} & A_{X}=0\begin{matrix} \begin{matrix} \begin{matrix} & \end{matrix} & \end{matrix} & \end{matrix} \end{matrix} \end{matrix} \end{matrix}\right.$ , (S.5.1)

$A_{X}^{out}=\left\{\begin{matrix} \frac{A_{X}}{A_{X}^{in}}\begin{matrix} & for\begin{matrix} & A_{X}\neq0,\begin{matrix} & \end{matrix} \end{matrix} \end{matrix} \\ 0,\begin{matrix} & for\begin{matrix} & A_{X}=0 \end{matrix} \end{matrix} \end{matrix}\right.$, (S.5.2)

References

1. Scholz M, Engel C, Loeffler M. Modelling human granulopoiesis under poly-chemotherapy with G-CSF support. J Math Biol. 2005; 50: 397–439. doi: 10.1007/s00285-004-0295-1.
