# Supplementary material for: Modeling individual time courses of thrombopoiesis during multi-cyclic chemotherapy
Source: PLoS Comput Biol. 2019 Mar 6;15(3):e1006775. doi: 10.1371/journal.pcbi.1006775 (PMC6422316; doi:10.1371/journal.pcbi.1006775)
Supplement: S6 Appendix — (DOCX) [file pcbi.1006775.s006.docx]

# **S6 Appendix. Properties of the modified Z-function**

Z- functions were designed to regulate a quantity between a minimum, a normal (steady state) and a maximum value in dependence on TPO. In the original form, it reads as follows:

$Z\left( X,y_{min},y_{nor},y_{max},b_{Y} \right)=y_{max}-\left( y_{max}-y_{min} \right)\cdot exp\left( -ln\left( \frac{y_{max}-y_{min}}{y_{max}-y_{nor}} \right)\cdot X^{b_{Y}} \right)=$

$y_{max}-\left( y_{max}-y_{min} \right)\cdot\left( \frac{y_{max}-y_{nor}}{y_{max}-y_{min}} \right)^{X^{b_{Y}}}$, (S.6.3)

where X is the relative TPO concentration. The parameter b_Y_ defines the steepness of the Z-function and is therefore called sensitivity parameter. This function is however numerically instable for high values of the sensitivity parameter, which can occur in cases of strong feedbacks. We stabilize our Z-functions in the present paper using a limiting parameter ${Lim}_{sig}$ of the steepness of the function. In detail:

$Z\left( X,y_{min},y_{nor},y_{max},b_{Y},{Lim}_{sig} \right)=$

$y_{max}-\left( y_{max}-y_{min} \right)\cdot exp\left( -ln\left( \frac{y_{max}-y_{min}}{y_{max}-y_{nor}} \right)\cdot\mathrm{Trans}_{\tanh}\left( X^{b_{Y}},{Lim}_{sig} \right) \right)=$

$y_{max}-\left( y_{max}-y_{min} \right)\cdot\left( \frac{y_{max}-y_{nor}}{y_{max}-y_{min}} \right)^{\mathrm{Trans}_{\tanh}\left( X^{b_{Y}},{Lim}_{sig} \right)}$ (S.6.4)

$\mathrm{Trans}_{\tanh}\left( X^{b_{Y}},{Lim}_{sig} \right)=exp\left( {Lim}_{sig}\cdot tanh\left( \frac{\ln\left( X^{b_{Y}} \right)}{{Lim}_{sig}} \right) \right)=exp\left( {Lim}_{sig}\cdot tanh\left( \frac{b_{Y}\cdot ln\left( X \right)}{{Lim}_{sig}} \right) \right)$. (S.6.5)

The hyperbolic tangent transformation $\mathrm{Trans}_{\tanh}$ of *X^by^* is an approximation of *X^by^* for small parameters *b_Y_* and values of X close to 1 (see S.6.6), but is limited by exp(*Lim_sig_*) for large values of X. Since the parameter ${Lim}_{sig}$ was poorly identifiable, we fixed it to 7.

$\begin{matrix} \begin{matrix} \frac{{d\mathrm{Trans}}_{\tanh}\left( Y,{Lim}_{sig} \right)}{dY}= \\ = {Lim}_{sig}\cdot exp\left( {Lim}_{sig}\cdot tanh\left( \frac{\ln\left( Y \right)}{{Lim}_{sig}} \right) \right)\cdot\frac{1}{{{Lim}_{sig}\cdot Y\cdot cosh\left( \frac{\ln\left( Y \right)}{{Lim}_{sig}} \right)}^{2}}= \end{matrix} \\ =\exp\left( {Lim}_{sig}\cdot tanh\left( \frac{\ln\left( Y \right)}{{Lim}_{sig}} \right) \right)\cdot\frac{1}{{Y\cdot cosh\left( \frac{\ln\left( Y \right)}{{Lim}_{sig}} \right)}^{2}} \\ \left. \frac{{d\mathrm{Trans}}_{\tanh}\left( Y,{Lim}_{sig} \right)}{dY} \right|_{Y=1}=1 \end{matrix}$. (S.6.6)

Fig 1 shows comparisons of original and modified Z-function for three settings of the sensitivity parameter *b_Y_*. Parameters *y_min_, y_nor_* and *y_max_* are set to 0, 1, 10, respectively. A difference between former and novel Z-function can only be detected for large values of the regulation function. Modified Z-functions with very high b_y_ can be considered as smoothed step functions in a neighborhood of the steady state of TPO.

As can be seen from the formula (S.6.4), the modified Z-function has a smaller range compared to the original Z-function. However, due to our setting of the parameter *Lim_sig_*, the difference is without practical relevance.

Fig 1: Comparison of original and modified Z-function. Relative TPO is between 0 and 5. Sensitivity parameter b takes values 1, 3 and 6, respectively. *y_min_, y_nor_* and *y_max_* are set to 0, 1, 10, respectively. The functions start to be different near the upper limit of their range.
