# Supplementary material for: Modeling individual time courses of thrombopoiesis during multi-cyclic chemotherapy
Source: PLoS Comput Biol. 2019 Mar 6;15(3):e1006775. doi: 10.1371/journal.pcbi.1006775 (PMC6422316; doi:10.1371/journal.pcbi.1006775)
Supplement: S7 Appendix — (DOCX) [file pcbi.1006775.s007.docx]

# **S7 Appendix. Transit times and amplification of CM sub-compartments.**

$n_{CM}^{reg}$ is the number of (TPO dependent) cell divisions in the early sub-compartments and is regulated via a Z-function of the relative TPO concentration (sum of pegylated and endogenous TPO divided by steady-state endogenous TPO values). $n_{CM}^{unreg}$ is the number of (TPO independent) cell divisions in the late sub-compartments:

$A_{CM,i}=exp\left( \frac{1}{n_{CM}^{e}}\cdot\ln\left( A_{CM} \right) \right), i=1,\cdots,n_{CM}^{e}$ (S.7.1)

$A_{CM,i}=\exp\left( \frac{n_{CM}^{unreg}}{n_{CM}^{l}}\cdot\ln\left( 2 \right) \right), i=n_{CM}^{e}+1,\cdots,n_{CM}^{l}$ (S.7.2)

$T_{CM,i}=\max\left( {T_{cycl}\cdot\log}_{2}\left( A_{CM,i} \right),0.1h \right), i=1,\cdots,n_{CM}$. (S.7.3)

The lower limit of *T_CM,i_* was set to 0.1h for numerical reasons. It can only be achieved if *A_CM_* is very small (very low TPO levels or severely injured bone marrow, both scenarios were not considered in the present publication).
