# Supplementary material for: Modeling individual time courses of thrombopoiesis during multi-cyclic chemotherapy
Source: PLoS Comput Biol. 2019 Mar 6;15(3):e1006775. doi: 10.1371/journal.pcbi.1006775 (PMC6422316; doi:10.1371/journal.pcbi.1006775)
Supplement: S8 Appendix — (DOCX) [file pcbi.1006775.s008.docx]

# **S8 Appendix. TPO-dependent probabilities for MKC either to form proplatelets or to undergo additional endomitosis**

We remind that *p_2_^k^_,1_* describe the probabilities to form pro-platelets and probabilities $p_{2^{k},2}$describe the transition of MKC to the next ploidy stage.

For k=3,4,5,6 the parameter p_2_^k^_,1_ depends on delayed TPO action:

$\begin{matrix} p_{2^{k},1}=Z\left( {Del}_{TPO,rel},p_{2^{k},1}^{min},p_{2^{k},1}^{nor},p_{2^{k},1}^{max},b_{{MKC}_{p,1}},{Lim}_{sig} \right), & k=3, 4, 5 \end{matrix}$ (S.8.1)

$p_{64,1}=\min\left( 1,p_{64,1}^{min}+\left( 1-p_{64,1}^{min} \right)\cdot Z_{\mathrm{simp}}\left( {Del}_{TPO,rel},b_{{MKC}_{p,64,1}},{Lim}_{sig} \right) \right)$, (S.8.2)

where Z_simp_ is a more parsimonious version of the Z-function (see S6 Appendix):

$Z_{\mathrm{simp}}\left( X,b,{Lim}_{sig} \right)=\exp\left( 1-\mathrm{Trans}_{\tanh}\left( X^{b_{Y}},{Lim}_{sig} \right) \right)$. (S.8.3)

We remind that the function $\mathrm{Trans}_{\tanh}\left( X^{b_{Y}},{Lim}_{sig} \right)$ is close to $X^{b_{Y}}$ near steady-state values. See also S6 Appendix. Consequently

$Z_{\mathrm{simp}}\left( X,b,{Lim}_{sig} \right)\sim\exp\left( 1-X \right)$ if *X* is close to 1.

$\begin{matrix} \frac{d}{dt}{Del}_{TPO,rel}=\frac{{{TPO}_{rel}-Del}_{TPO,rel}}{T_{Del}} \\ \frac{d}{dt}{Del}_{TPO,rel,2^{5}}=\frac{{Del}_{TPO,rel}-{Del}_{TPO,rel,2^{5}}}{T_{Del,2^{5}}} \end{matrix}$, (S.8.4)

where *Del_TPO,rel_* serves as the argument of regulatory functions of relative TPO concentration on MKC except mobilization of dormant MKC of ploidy 32, which is described by longer delay ${Del}_{TPO,rel,2^{5}}$.

Assuming that $p_{2^{k},2}$ for k = 3,4,5 depends on TPO did not improve fits and we assumed that these probabilities are constant and equal to $p_{2}$.
