# Supplementary material for: Modeling individual time courses of thrombopoiesis during multi-cyclic chemotherapy
Source: PLoS Comput Biol. 2019 Mar 6;15(3):e1006775. doi: 10.1371/journal.pcbi.1006775 (PMC6422316; doi:10.1371/journal.pcbi.1006775)
Supplement: S9 Appendix — (DOCX) [file pcbi.1006775.s009.docx]

# **S9 Appendix. Comparison of total TPO consumption by CM, megakaryocytes, proplatelets and platelets**

In our model, we compared the TPO consumption by megakaryocytes to that of platelets. We neglected TPO consumption by colony-forming units. Here, we justify these assumptions. We first summarize the relevant biological data:

According to [1], each platelet has

- 25-224 receptors with affinity 128 and 846 pmol/L to TPO (analysis of platelet-rich plasma (PRP) using different preparations).
- 25- 35 receptors with affinity 260 and 540 pmol/L to TPO (washed platelets (WP)).

According to [2], each megakaryocyte produces 1000-5000 platelets with a median value of 3000.

According to [3]

- Each megakaryocyte has on average 12,140 TPO receptors with affinity 749 PM.
- Each CM has on average 1,223 TPO receptors with affinity 223 PM.
- The exposure of MKC and CM to TPO reduces the number of receptors by 60% within three hours.

After binding of TPO, the receptors are internalized and degraded. According to the above mentioned data, we assume that each platelet has 30 TPO receptors and each megakaryocyte has 12140/8 TPO receptors per cell unit, since modal ploidy of megakaryocytes is 16. Each proplatelet is assumed to have 12140/8 TPO receptors as well (proplatelets are calculated per cell unit, see formula (23,24)). We assume that specific TPO consumption is proportional to the number of TPO receptors. Consequently:

$\begin{matrix} \begin{matrix} w_{PLC}=w_{re}\cdot30 \\ w_{MKC,k}=w_{re}\cdot2^{k}\cdot\frac{12140}{8}\cdot\left( C_{MKC,act,P2^{k}}+C_{MKC,dorm,P2^{k}} \right)\begin{matrix} , & k=3,4,5 \end{matrix} \end{matrix} \\ \begin{matrix} w_{MKC,k}=w_{re}\cdot2^{k}\cdot\frac{12140}{8}\cdot C_{MKC,act,P2^{k}}\begin{matrix} , & k=1,2,6,7 \end{matrix} \\ w_{PP}=w_{re}\cdot\frac{12140}{8}\cdot PP \end{matrix} \end{matrix}$. (S.9.1)

Parameter *w_re_* defines the maximum specific elimination rate per TPO receptor (units rec^-1^h^-1^) In order to avoid small coefficients, we normalized *w_re_* by the total number of receptors on all blood platelets for patient with normal platelets count:

$\hat{w}_{re}={30\cdot{230\cdot BV\cdot10}^{9}\cdot w}_{re}$, (S.9.2)

where BV is the blood volume. The parameter $\hat{w}_{re}$ is estimated.

A single CM with 1,223 receptors give rise to 3000 platelets with 30 receptors, i.e. total 90,000 receptors with similar affinities.. Thus, platelets carry about 74 times more receptors than CM. This justifies our simplification to ignore TPO elimination by CM.

References

1. Fielder PJ, Hass P, Nagel M, Stefanich E, Widmer R, Bennett GL, et al. Human platelets as a model for the binding and degradation of thrombopoietin. Blood. 1997; 89: 2782–2788.

2. Bernstein SH, Jusko WJ, Krzyzanski W, Nichol J, Wetzler M. Pharmacodynamic modeling of thrombopoietin, platelet, and megakaryocyte dynamics in patients with acute myeloid leukemia undergoing dose intensive chemotherapy. J Clin Pharmacol. 2002; 42: 501–511.

3. Sato T, Fuse A, Niimi H, Fielder PJ, Avraham H. Binding and regulation of thrombopoietin to human megakaryocytes. Br J Haematol. 1998; 100: 704–711.
