# Supplementary material for: Modeling individual time courses of thrombopoiesis during multi-cyclic chemotherapy
Source: PLoS Comput Biol. 2019 Mar 6;15(3):e1006775. doi: 10.1371/journal.pcbi.1006775 (PMC6422316; doi:10.1371/journal.pcbi.1006775)
Supplement: S10 Appendix — (DOCX) [file pcbi.1006775.s010.docx]

# **S10 Appendix. Estimation of steady state and initial values from initially measured platelet counts**

## **Steady state and initial values of state variables**

Initial values of almost all variables are assumed to be in steady state. However, we assumed that initial values of endogenous TPO (*C_TPO_*_,_*_endo_* ) and PLC could differ from their steady-state values due to random fluctuations. Indeed, it was observed that platelet counts in healthy untreated people are oscillating [1]. The authors hypothesized that this is caused by random fluctuations in platelet consumption. In our model, this results in oscillations due to the delayed feedback of circulating platelets to precursors. Thus, for modelling individual data, we assume that initial endogenous TPO concentrations and platelets are realizations of random variables lognormally distributed around an unknown individual steady state (see (S.13.15) in S13 Appendix for details).

No such deviations from steady state are assumed for simulations of average population data.

## **Estimation of steady state values**

In this section, we derive the steady state values of all state variables. Because on the individualized parameter settings, steady state values differ between subjects. We assume that the initial pre-treatment platelet count of each patient is the realization of a random lognormal variable with median value equal to the individual steady state value *PL_0_* as defined in S.13.15, S13 Appendix. More precisely, we assume that the initial values are lognormally distributed with median *PL_0_* and variance 0.1. The variance is derived from data of platelet fluctuations as observed for healthy untreated patients [1].

We define the total platelets elimination coefficient as

$k_{tot}=\frac{1}{T_{PL}^{sub}}+E(Circ)$, (S.10.1)

where elimination Effect E*(Circ)* is defined in (30). We derive steady state values of each variable of the system from *PL_0_* as follows.

Since platelet elimination from each circulating sub-compartment is nonlinear, its steady-state values are derived recursively from (31-34). Given the efflux $\hat{C}_{PP}^{out}$ from the last megakaryocytes compartment defined in (24), it follows:

$\hat{C}_{{PLS}_{1}}=\frac{{npt}_{pcu}\cdot T_{PL}^{sub}\cdot\left( 1-k_{circ} \right)}{1+{T_{PL}^{sub}\cdot ksc}_{1}^{SC}}\cdot\hat{C}_{PP}^{out}$ (S.10.2)

$\hat{C}_{{PLC}_{1}}=\frac{{npt}_{pcu}\cdot k_{circ}}{k_{tot}}\cdot\hat{C}_{PP}^{out}+\frac{{ksc}_{1}^{SC}}{k_{tot}}\cdot\hat{C}_{{PLS}_{1}}$ (S.10.3)

$\hat{C}_{{PLS}_{i}}=\frac{\hat{C}_{{PLS}_{i-1}}}{1+T_{PL}^{sub}\cdot{ksc}_{i}^{SC}}, i=2,\cdots,n$ (S.10.4)

$\hat{C}_{{PLC}_{i}}=\frac{\hat{C}_{{PLC}_{i-1}}}{k_{tot}\cdot T_{PL}^{sub}}+\frac{{ksc}_{i}^{SC}}{k_{tot}}\cdot\hat{C}_{{PLS}_{i}}, i=2,\cdots,n$. (S.10.5)

This implies that for a given value of $\hat{C}_{PP}^{out}$ the resulting estimates $\left\{ \hat{C}_{{PLS}_{i}} \right\}_{i=1}^{n}$ and $\left\{ \hat{C}_{{PLC}_{i}} \right\}_{i=1}^{n}$are proportional to the real steady state values of the respective sub-compartments with the coefficient of proportionality equal to $\frac{{PL}_{0}}{\sum_{i=1}^{n} \hat{C}_{{PLC}_{i}}}$:

$\begin{matrix} C_{PP}^{out,nor}=\frac{{PL}_{0}}{\sum_{i=1}^{n} \hat{C}_{{PLC}_{i}}}\cdot\hat{C}_{PP}^{out} \\ C_{{PLS}_{i}}^{nor}=\frac{{PL}_{0}}{\sum_{i=1}^{n} \hat{C}_{{PLC}_{i}}}\cdot\hat{C}_{{PLS}_{i}} \\ C_{{PLC}_{i}}^{nor}=\frac{{PL}_{0}}{\sum_{i=1}^{n} \hat{C}_{{PLC}_{i}}}\cdot\hat{C}_{{PLC}_{i}} \end{matrix},i=1,\cdots,n$. (S.10.6)

From (24) follows that

$C_{PP}^{nor}=C_{PP}^{out,nor}\cdot T_{PP}$. (S.10.7)

Now we estimate steady state values of other precursors. First of all, we use the estimated steady state value of the proplatelet compartment in order to deduce steady state values of all megakaryocytes sub-compartments. In complete analogy to calculation of $C_{PP}^{out,nor}$, we fix$C_{MKC,act,P64}^{nor}$ to an arbitrary value $\hat{C}_{MKC,act,P64}^{nor}$. Then, we calculate all other sub-compartments recursively, and finally, normalize them. It is important to keep in mind that in steady state $C_{C_{MKC,act,P128}}^{nor}=0$. From (15-18) it follows that:

$\hat{C}_{MKC,act,P32}^{nor}=\frac{T_{endo}}{p_{32,2}\cdot\left( 1-p_{32,1}^{nor} \right)\cdot T_{PP}}\cdot\hat{C}_{MKC,act,P64}^{nor}$ (S.10.8)

$\hat{C}_{MKC,dorm,P32}^{nor}=\frac{k_{dorm,32}^{nor}}{k_{rev\_dorm,32}^{nor}}{\cdot\hat{C}}_{MKC,act,P32}^{nor}$ (S.10.9)

$\hat{C}_{MKC,act,P16}^{nor}=\frac{T_{endo}}{p_{16,2}\cdot\left( 1-p_{16,1}^{nor} \right)}\cdot\left( \frac{p_{32,2}\cdot\left( 1-p_{32,1}^{nor} \right)}{T_{endo}}+\frac{p_{32,1}^{nor}}{T_{PP}} \right)\cdot\hat{C}_{MKC,act,P32}^{nor}$ (S.10.10)

$\hat{C}_{MKC,dorm,P16}^{nor}=\frac{k_{dorm,16}^{nor}}{k_{rev\_dorm,16}^{nor}}\cdot\hat{C}_{MKC,act,P16}^{nor}$ (S.10.11)

$\hat{C}_{MKC,act,P8}^{nor}=\frac{T_{endo}}{p_{8,2}\cdot\left( 1-p_{8,1}^{nor} \right)}\cdot\left( \frac{p_{16,2}\cdot\left( 1-p_{16,1}^{nor} \right)}{T_{endo}}+\frac{p_{16,1}^{nor}}{T_{PP}} \right)\cdot\hat{C}_{MKC,act,P16}^{nor}$ (S.10.12)

$\hat{C}_{MKC,dorm,P8}^{nor}=\frac{k_{dorm,8}^{nor}}{k_{rev\_dorm,8}^{nor}}\cdot\hat{C}_{MKC,act,P8}^{nor}$ (S.10.13)

$\hat{C}_{MKC,act,P2}^{nor}=\hat{C}_{MKC,act,P4}^{nor}=T_{endo}\cdot\left( \frac{p_{8,2}\cdot\left( 1-p_{8,1}^{nor} \right)}{T_{endo}}+\frac{p_{8,1}^{nor}}{T_{PP}} \right)\cdot\hat{C}_{MKC,act,P8}^{nor}$. (S.10.14)

From (20) we obtain

$\begin{matrix} k_{dorm,8}^{nor}=\frac{\left( 1-p_{8,1}^{nor} \right)\cdot\left( 1-p_{8,2} \right)}{T_{dorm,MKC}} \\ k_{dorm,16}^{nor}=\frac{\left( 1-p_{16,1}^{nor} \right)\cdot\left( 1-p_{16,2} \right)}{T_{dorm,MKC}} \\ k_{dorm,32}^{nor}=\frac{\left( 1-p_{32,1}^{nor} \right)\cdot\left( 1-p_{32,2} \right)}{T_{dorm,MKC}} \end{matrix}$. (S.10.15)

Assuming steady state for (23) and substituting (S.10.8-S.10.14) we obtain that

$\hat{C}_{PP}^{nor}=\sum_{k=3}^{6} \left( 2^{k-1}\cdot\hat{C}_{MKC,act,P2^{k}}^{nor} \right)$. (S.10.16)

Comparing (S.10.16) and (S.10.7) we deduce that all estimates (S.10.8-S.10.14) must be multiplied by the factor $\frac{C_{PP}^{nor}}{\hat{C}_{PP}^{nor}}$ to obtain steady-state values:

$\begin{matrix} {C_{MKC,act,Pi}^{nor}=\frac{C_{PP}^{nor}}{\hat{C}_{PP}^{nor}}\cdot\hat{C}}_{MKC,act,Pi}^{nor}\begin{matrix} , & i=2,\cdots,64 \end{matrix} \\ {C_{MKC,dorm,Pi}^{nor}=\frac{C_{PP}^{nor}}{\hat{C}_{PP}^{nor}}\cdot\hat{C}}_{MKC,dorm,Pi}^{nor}\begin{matrix} , & i=8, 16, 32 \end{matrix} \end{matrix}$. (S.10.17)

From (S.7.1-S.7.3, S7 Appendix) follows that

$\begin{matrix} A_{CM,i}^{nor}=A_{CM,i}^{nor}=\exp\left( \frac{1}{n_{CM}^{e}}\cdot\ln\left( 2 \right) \right), i=1,\cdots,n_{CM}^{e} \\ {A_{CM,i}^{nor}=A}_{CM,i}=\exp\left( \frac{n_{CM}^{unreg}}{n_{CM}^{l}}\cdot\ln\left( 2 \right) \right), i=n_{CM}^{e}+1,\cdots,n_{CM}^{l} \end{matrix}$ . (S.10.18)

Consequently, $A_{CM,i}^{in,nor}$ and $A_{CM,i}^{out,nor}$ can be derived from (S.5.1-S.5.2).

From (14) it follows that:

$C_{CM,n_{CM}}=\frac{T_{CM,n_{CM}}\cdot C_{MKC,act,P2}}{T_{endo}\cdot A_{CM,n_{CM}}^{out}}$. (S.10.19)

From (11,12) the following recursive relations are obtained:

$C_{CM,i-1}^{nor}=\frac{T_{CM,i-1}\cdot C_{CM,i}^{nor}}{T_{CM,i}\cdot A_{CM,i}^{in,nor}\cdot A_{CM,i-1}^{out,nor}}$, $i=2,\cdots,n_{CM}^{e}$. (S.10.20)

Remembering that in steady-state it holds p=0.5. Hence,

$C_{S}^{nor}=\frac{{T_{cycle}\cdot C}_{S}^{out}}{2\cdot\left( 1-p \right)}=\frac{T_{cycle}\cdot C_{CM,1}^{nor}}{T_{CM,1}{\cdot A}_{CM,1}^{in,nor}}$*.*  (S.10.21)

From (10) it follows that:

$C_{dorm}^{nor}=\frac{C_{S}^{nor}\cdot k_{act}}{k_{dorm}}$. (S.10.22)

References

1. Schulthess GK von, Gessner U. Oscillating platelet counts in healthy individuals: experimental investigation and quantitative evaluation of thrombocytopoietic feedback control. Scand J Haematol. 1986; 36: 473–479.
