# Supplementary material for: Modeling individual time courses of thrombopoiesis during multi-cyclic chemotherapy
Source: PLoS Comput Biol. 2019 Mar 6;15(3):e1006775. doi: 10.1371/journal.pcbi.1006775 (PMC6422316; doi:10.1371/journal.pcbi.1006775)
Supplement: S11 Appendix — (DOCX) [file pcbi.1006775.s011.docx]

# **S11 Appendix. Transition of aged platelets from spleen to circulation**

In steady-state the spleen contains approximately one third of all platelets [1]. Young platelets preferentially sequester in the spleen. Then, they are age-dependently released. In this section we derive corresponding transition coefficients $k_{i}^{sc}$ in analogy to our former model [2]. We assumed the same age-dependent linearly increasing proportion *g_i_* of platelets of age *i* in circulation:

$\begin{matrix} g_{i}=2\left( q-g_{1} \right)\frac{i-1}{n-1}+g_{1}\begin{matrix} , & i=2,\ldots,n \end{matrix} \\ G_{i}\equiv\frac{C_{PLC_{i}}^{nor}}{C_{PLS_{i}}^{nor}}=\frac{g_{i}}{1-g_{i}} \end{matrix}$, (S.11.1)

where q=2/3, g_1_ is a free parameter to be determined later.

According to (S.10.6)$\hat{C}_{{PLS}_{i}}$ and $\hat{C}_{{PLC}_{i}}$ are proportional to $C_{{PLS}_{i}}^{nor}$ and $C_{{PLC}_{i}}^{nor}$ respectively. This implies that relations (S.10.2, S.10.3) are valid for the steady state values $C_{{PLS}_{i}}^{nor}$ and $C_{{PLC}_{i}}^{nor}$. Dividing both sides of (S.10.2) by the respective both sides of (S.10.3) we can derive $\frac{C_{{PLC}_{1}}^{nor}}{C_{{PLS}_{1}}^{nor}}$. In combination with (S.11.1) it follows that:

$\frac{C_{{PLC}_{1}}^{nor}}{C_{{PLS}_{1}}^{nor}}=\frac{g_{1}}{1-g_{1}}=\frac{k_{circ}}{k_{tot}}\cdot\frac{1+{T_{PL}^{sub}\cdot ksc}_{1}^{SC}}{T_{PL}^{sub}\cdot\left( 1-k_{circ} \right)}+\frac{k_{1}^{SC}}{k_{tot}}$. (S.11.2)

From this follows immediately that

$k_{1}^{SC}=k_{tot}\cdot\left( 1-k_{circ} \right)\cdot\frac{g_{1}}{1-g_{1}}-\frac{k_{circ}}{T_{PL}^{sub}}$. (S.11.3)

In the same way (S.10.6) implies that relations (S.10.4, S.10.5) are valid for the steady state. In combination with (S.11.1) it follows that:

$\frac{C_{PLC_{i}}^{nor}}{C_{PLS_{i}}^{nor}}=G_{i}=\frac{\hat{C}_{{PLC}_{i-1}}}{k_{tot}\cdot T_{PL}^{sub}\cdot\hat{C}_{{PLS}_{i}}}+\frac{k_{i}^{SC}}{k_{tot}}=G_{i-1}\frac{1+T_{PL}^{sub}\cdot k_{i}^{SC}}{k_{tot}\cdot T_{PL}^{sub}}+\frac{k_{i}^{SC}}{k_{tot}}\begin{matrix} , & i=2,\ldots,n \end{matrix}$. (S.11.4)

From this equation it follows that:

$k_{i}^{SC}=\frac{k_{tot}\cdot\left( G_{i}-\frac{G_{i-1}}{k_{tot}\cdot T_{PL}^{sub}} \right)}{G_{i-1}+1}\begin{matrix} , & i=2,\ldots,n \end{matrix}$. (S.11.5)

Since *g_i_* is an increasing sequence in (0,1), it can be derived from (S.11.1) in analogy to [2] that

$q{>g}_{1}>\frac{1}{3}$. (S.11.6)

(S.11.3) is positive when

$\frac{g_{1}}{1-g_{1}}>\frac{k_{circ}}{{{\left( 1-k_{circ} \right)\cdot k}_{tot}\cdot T}_{PL}^{sub}}$.

Since ${k_{tot}\cdot T}_{PL}^{sub}>1$ (see (S.10.1)), then a sufficient condition for a positive value of $k_{1}^{SC}$ is

$\frac{g_{1}}{1-g_{1}}>\frac{k_{circ}}{\left( 1-k_{circ} \right)}$,

which is equivalent to $g_{1}>k_{circ}$ when *g_1_* is in between 0 and 1. Thus, a sufficient condition for admissible model parameters is the same as in our former model:

$q{>g}_{1}>max\left\{ k_{circ},\frac{1}{3} \right\}$. (S.11.7)

We choose *g_1_*=0.34. Equation (S.11.5) is always positive, because ${k_{tot}\cdot T}_{PL}^{sub}>1$ and $\left\{ g_{i} \right\}$ is an increasing sequence.

References

1. Freedman M, Altszuler N, Karpatkin S. Presence of a nonsplenic platelet pool. Blood. 1977; 50: 419–425.

2. Scholz M, Gross A, Loeffler M. A biomathematical model of human thrombopoiesis under chemotherapy. J Theor Biol. 2010; 264: 287–300. doi: 10.1016/j.jtbi.2009.12.032.
