# Supplementary material for: Modeling individual time courses of thrombopoiesis during multi-cyclic chemotherapy
Source: PLoS Comput Biol. 2019 Mar 6;15(3):e1006775. doi: 10.1371/journal.pcbi.1006775 (PMC6422316; doi:10.1371/journal.pcbi.1006775)
Supplement: S12 Appendix — (DOCX) [file pcbi.1006775.s012.docx]

# **S12 Appendix. Labeled platelets**

To model the labelling data of Hanson et al., we introduced a compartment of labeled platelets, assuming that these platelets have the same parameters as native once. The ODE describing the labeled transfused platelets are the same as (31, 33), except for the lack of influx from bone marrow and spleen. In this case it holds that $Circ=\sum_{i=1}^{n} \left( C_{{PLC}_{i}}+C_{PLC,i}^{l} \right)$ where $C_{PLC,i}^{l}$, *i=1,…,n* denote the labeled platelet age-compartments in circulation.
