# Supplementary material for: Modeling individual time courses of thrombopoiesis during multi-cyclic chemotherapy
Source: PLoS Comput Biol. 2019 Mar 6;15(3):e1006775. doi: 10.1371/journal.pcbi.1006775 (PMC6422316; doi:10.1371/journal.pcbi.1006775)
Supplement: S13 Appendix — (DOCX) [file pcbi.1006775.s013.docx]

# **S13 Appendix. Log-likelihood construction.**

In this chapter, we provide more detailed information regarding our procedure of parameter fitting. Since we combine several data sets in order to fit the parameters, it is necessary to construct an integrated likelihood function.

## **Construction of an integrated likelihood function**

During estimation we transformed parameters within a certain range (a,b) by the *logit-* function and parameters with positive values by lognormal transformation as follows:

$\varphi_{s}=h_{s}\left( \psi_{s} \right)$ (S.13.1)

$h_{s}\left( \psi_{s} \right)=\left\{ \begin{aligned} e^{\psi_{s}}\begin{matrix} , & for parameters>0 \end{matrix} \\ a+\left( b-a \right)\cdot\frac{e^{\psi_{s}}}{1+e^{\psi_{s}}}\begin{matrix} , & for parameters within [a,b] \end{matrix} \end{aligned} \right.$. (S.13.2)

Here, $\varphi_{i}$ is the *i*-th biological parameter and $\psi_{i}$ is the corresponding transformed parameter, whose values are fitted during maximization of the likelihood function. These transformations allow maximization of the likelihood over *R^n^* space (*n* = number of parameters).

The population standard deviation of each transformed parameter $\psi_{s}$ is named $\omega_{s}$. It is worth to remember that when $\omega_{s}$ is small and when transformations $h_{s}$ are lognormal, $\omega_{s}$ is close to the standard deviation of untransformed parameters $\varphi_{s}$.

We adopt the formalism from Faller et al [1] in order to describe the goal functions to be optimized. A solution of any ODE model results in a function ***x***, whose arguments are time t and parameter vector ψ and which takes value is a multidimensional space of the modelled variables:

$\left\{ \begin{matrix} \dot{\vec{x}}\left( t,\vec{\psi} \right)=\vec{f}\left( \vec{x},\vec{\psi},t \right) \\ \vec{x}\left( t_{0} \right)=\vec{X}_{0} \end{matrix} \right.$. (S.13.3)

Suppose that a model is designed to describe a set of experiments, which measure L properties (outcomes) *y^1^_D_(t),…,y^L^_D_(t)*, where subscript *D* stands for data. Suppose that these *L* properties can be fitted as *y^1^_M_(ψ,t),…,y^L^_M_(ψ,t)* using the model (S.13.3), where subscript *M* stands for modelled features.

Observation functions *g^1^*,…, *g^O^* describe available time series data of *O* state-variables:

$\left\{ \begin{matrix} y_{M}^{l}\left( \vec{\psi},t \right)=g^{k_{l}}\left( \vec{x}\left( t,\vec{\psi} \right) \right) \\ y_{D}^{l}\left( t_{l,j} \right)=y_{M}^{l}\left( \vec{\psi},t_{l,j} \right)+\varepsilon_{l,j} \end{matrix} \right.\begin{matrix} , & l=1,\cdots,L, \end{matrix}\begin{matrix} \begin{matrix} , & j=1,\cdots,n_{l} \end{matrix}, & k_{l}\in\left\{ 1,\cdots,O \right\}, \end{matrix}\begin{matrix} & \forall l \end{matrix}$. (S.13.4)

The observation functions for our data are specified later (see sub-section “The observation functions, weights and residual errors”). Here, we assume that the *l*-th property has been measured *n_l_* times. The observed and modelled properties differ according to (S.13.4) by errors *ɛ_l,j_*, which are assumed to be mutually independent. Similar to [1] we assume that for each *l* and *j* the corresponding error is a normally distributed random variable:

$\varepsilon_{l,j}\sim N\left( 0,\sigma_{l,j}\left( \vec{\theta} \right) \right)\begin{matrix} , & l\begin{matrix} =1,\cdots,L, & j=1,\cdots,n_{l} \end{matrix} \end{matrix}$. (S.13.5)

where the corresponding standard deviations {σ_l,j_} can depend on the parameter set θ.

In a more general case, when *N* different patients take part in *L* experiments, eq. (S.13.4) reads as follows

$\left\{ \begin{matrix} y_{M}^{l}\left( \vec{\psi}^{pop},\vec{\psi}_{i}^{ind},t \right)=g^{k_{l}}\left( \vec{x}\left( t,\vec{\psi}^{pop},\vec{\psi}_{i}^{ind} \right) \right) \\ y_{D}^{l}\left( t_{l,i,j} \right)=y_{M}^{l}\left( \vec{\psi}^{pop},\vec{\psi}_{i}^{ind}{,t}_{i,l,j} \right)+\varepsilon_{i,l,j} \end{matrix} \right.$

$i=1,\cdots,N\begin{matrix} , & l=1,\cdots,L \end{matrix}\begin{matrix} , & j=1,\cdots,n_{i,l} \end{matrix}\begin{matrix} , & k_{l}\in\left\{ 1,\cdots,O \right\} \end{matrix} ,\forall l$. (S.13.6)

Here we assume that the *l*-th experiment has *n_i,l_* measurements for the *i*-th subject. The parameters can be either individual $\left\{ \vec{\psi}_{l,i}^{ind} \right\}_{i=1}^{N}$ or population-based $\vec{\psi}^{pop}$. We assume in this work that parameters of residual error distributions are individual:

$\left\{ \begin{matrix} \vec{\psi}=\left\{ \left\{ \vec{\psi}_{l,i}^{ind} \right\}_{i=1}^{N} , \vec{\psi}^{pop} \right\} \\ \vec{\theta}=\left\{ \vec{\theta}_{i,l} \right\}_{i=1,l=1}^{N,L} \end{matrix} \right.$. (S.13.7)

We also assume that residual errors *ɛ_i,l,_*_j_ are mutually independent with the following distribution:

$\varepsilon_{i,l,j}\sim N\left( 0,\sigma_{i,l}\left( \vec{\theta}_{i,l} \right) \right),i=1,\cdots,N\begin{matrix} , & l=1,\cdots,L \end{matrix}\begin{matrix} , & j=1,\cdots,n_{i,l} \end{matrix}$. (S.13.8)

Thus, no autocorrelations of errors were assumed. The aim of likelihood-based parameter estimation is, in general, to find parameters of a model resulting in the highest likelihood of the experimental data [2]. In our case, we are looking for a parameter vector pair *(*θ*^*^, ψ^*^)* which maximizes the joint probability density of all observations:

$\left( \vec{\psi}^{*},\vec{\theta}^{*} \right)=\underset{\left( \vec{\psi},\vec{\theta} \right)}{\mathrm{argmax}} \left\{ p\left( \bigcap_{i=1}^{N} \bigcap_{l=1}^{L} \bigcap_{j=1}^{n_{i,l}} \left( y_{D}^{i,l}\left( t_{i,l,j} \right)-y_{M}^{l}\left( \vec{\psi}_{i}^{ind},\vec{\psi}^{pop},t \right)\left| \vec{\psi}_{i}^{ind},\vec{\psi}^{pop},\sigma_{i,l,j}\left| \vec{\theta}_{i,l} \right. \right. \right) \right) \right\}=$

$\underset{\left( \vec{\psi},\vec{\theta} \right)}{=argmax} \left\{ \prod_{i=1}^{N} \prod_{l=1}^{L} \prod_{j=1}^{n_{i,l}} p\left( y_{D}^{i,l}\left( t_{i,l,j} \right)-y_{M}^{l}\left( \vec{\psi}_{i}^{ind},\vec{\psi}^{pop},t \right)\left| \vec{\psi}_{i}^{ind},\vec{\psi}^{pop},\sigma_{i,l,j},\vec{\theta}_{i,l} \right. \right) \right\}$. (S.13.9)

We optimize the negative log-likelihood, i.e.

$\left\{ \begin{matrix} \left( \vec{\psi}^{*},\vec{\theta}^{*} \right)=\underset{\left( \vec{\psi},\vec{\theta} \right)}{\mathrm{argmin}}\left( nLL \right) \\ nLL=\sum_{i=1}^{N} {nLL}_{i} \end{matrix} \right.$.. (S.13.10)

where *nLL* is the total negative log-likelihood and *nLL_i_* is the negative log-likelihood of the *i*-th experiment.

If the experiments have different numbers of observations, the experiments with more observations have more influence on parameter estimation resulting in biased fits. Moreover, differences in the accuracy of measurements might be possible between experiments. Taking these issues into account, we decided to fit weighted individual likelihoods:

${nLL}_{i}=\sum_{l=1}^{L} {We}_{i,l}\cdot\sum_{j=1}^{n_{i,l}} \left( \ln\left( \sigma_{i,l,j} \right)+\frac{\ln\left( 2\cdot\pi\right)}{2}+\frac{\left( y_{D}^{i,l}\left( t_{i,l,j} \right)-y_{M}^{l}\left( {\vec{\psi}_{i}^{ind},\vec{\psi}^{pop},t}_{i,l,j} \right) \right)^{2}}{\sigma_{i,l,j}^{2}\left( \vec{\theta}_{i,l} \right)} \right)$ (S.13.11)

where We_i,l_ are the weights.

The terms for individual and biological data were weighted empirically in order to minimize the bias of the fitting. This happens for example when a certain data set contains only a small number of observations or if there are qualitative differences between the data sets. The weighted likelihood method is applied to the problem of estimating the parameter of an exponential distribution when a proportion of the observations are outliers [3]. It was proved to be equivalent to the minimum bias procedure [4]. The weights for our cases are specified later when we present the observation functions.

In order to combine individual data with other experimental results we assume that individual subjects virtually participate in the other experiments and contribute to corresponding averaged measurements and respective standard deviations. We combine individual negative log-likelihood functions (S.13.11) with those of virtual experiments using the same corresponding individual parameter set $\vec{\psi}_{i}^{ind}$:

$\begin{matrix} {nLL}_{i}^{ind}=\sum_{l=1}^{L^{ind}} {We}_{i,l}^{ind}\cdot\sum_{j=1}^{n_{i,l}^{ind}} \left( \ln\left( \sigma_{i,l,j}^{ind} \right)+\frac{\ln\left( 2\cdot\pi\right)}{2}+\frac{\left( y_{D,ind}^{i,l}\left( t_{i,l,j}^{ind} \right)-y_{M}^{l}\left( \vec{\psi}_{i}^{ind},\vec{\psi}^{pop},t_{i,l,j}^{ind} \right) \right)^{2}}{{\sigma_{i,l,j}^{\mathrm{ind}}\left( \vec{\theta}_{i,l} \right)}^{2}} \right) \\ {nLL}_{i}^{av}=\sum_{l=1}^{L} {We}_{i,l}^{av}\cdot\sum_{j=1}^{n_{i,l}^{av}} \left( \ln\left( \sigma_{i,l,j}^{av} \right)+\frac{\ln\left( 2\cdot\pi\right)}{2}+\frac{\left( y_{D,ind}^{i,l}\left( t_{i,l,j}^{av} \right)-y_{M}^{l}\left( \vec{\psi}_{i}^{ind},\vec{\psi}^{pop},t_{i,l,j}^{av} \right) \right)^{2}}{{\sigma_{i,l,j}^{\mathrm{av}}\left( \vec{\theta}_{i,l} \right)}^{2}} \right) \\ {{{nLL}_{i}=nLL}_{i}^{ind}+nLL}_{i}^{av}+{nLL}_{i}^{pri} \end{matrix}$ . (S.13.12)

Here, superscripts *ind* and *av* correspond to individual and averaged biological observations, respectively. ${nLL}_{i}^{pri}$ denotes an additional part of individual negative likelihood, which gives a penalty for the deviations of individual parameter values from given prior values (either resulting from other fitting steps or from population-based distributions, as explained below):

${nLL}_{i}^{pri}=\sum_{k=1}^{N_{par}^{ind}} \delta_{k}\cdot\left( \frac{\left( \psi_{i,k}^{ind}-\psi_{k}^{pri} \right)^{2}}{\omega_{pri,k}^{2}}+0.5\cdot\ln\left( 2\pi\right)+\ln\left( \omega_{pri,k} \right) \right)$ , (S.13.13)

where $\delta_{k}$ equals 1, if a prior is assumed for the *k*-th parameter and 0 otherwise. $N_{par}^{ind}$ is the number of fitted individual parameters:

$\psi_{k}^{ind}\sim N\left( {\psi_{k}^{pri},\omega}_{pri,k} \right)=N\left( {h_{k}\left( \varphi_{k}^{pri} \right)^{-1},\omega}_{pri,k} \right)$. (S.13.14)

The reason for adding prior information is that initial PLC and TPO values should be close to individual steady states:

$\begin{matrix} {PL}^{nor}={PL}_{0}\cdot r_{PL,0,nor}, r_{PL,0,nor}=e^{\psi_{r_{PL,0,nor}}}\begin{matrix} , & \psi_{r_{PL,0,nor}}^{ind}\sim N\left( 0,\omega_{pri,r_{PL,0,nor}} \right) \end{matrix} \\ C_{TPO}^{nor}=\frac{TPO\left( t_{0} \right)}{r_{TPO,nor,0}}, r_{TPO,nor,0}=e^{\psi_{r_{TPO,nor,0}}}\begin{matrix} , & \psi_{r_{TPO,nor,0}}^{ind}\sim N\left( 0,\omega_{pri,r_{TPO,nor,0}} \right) \end{matrix} \end{matrix}$ . (S.13.15)

where steady state and initial platelet counts are calculated by summing the respective sub-compartments:

$\begin{matrix} {PL}^{nor}=\sum_{i=1}^{n} C_{PLC,i}^{nor}\left( \vec{\psi} \right) \\ {PL}_{0}=\sum_{i=1}^{n} C_{{PLC}_{i}}\left( t_{0},\vec{\psi} \right) \end{matrix}$ .

It needs to be acknowledged that steady state values of cancer patients can be different from those of healthy subjects due to cytokine-producing tumors or general inflammation. These issues are not covered by the present model version.

According to publically available data of platelets count monitoring over 2-3 months for two patients [5], platelet counts are normally distributed around their average value with standard deviation of the logarithmized values $\omega_{r_{TPO,nor,0}}$close to 0.1. Thus, we fixed $\omega_{pri,r_{PL,0,nor}}$ to 0.1 for the Engel et al. study. It turned out that during fitting of NHL data, the resulting standard deviations $\omega_{r_{PL,0,nor}}$ of $\psi_{r_{PL,0,nor}}$ exceeded significantly the assumed prior values. We found empirically that setting $\omega_{pri,r_{TPO,nor,0}}$to 0.061 results in $\omega_{r_{TPO,nor,0}}$ to be nearly 0.1. No data are available regarding $\omega_{pri,r_{TPO,nor,0}}$, but we hypothesize that it is larger than $\omega_{pri,r_{PL,0,nor}}$ because of less regular behavior of TPO dynamics compared to that of PLC as observed in Engel et al. Specifically, we fixed $\omega_{pri,r_{TPO,nor,0}}$ to be equal to 0.5.

During parameter fitting of the NHL-B data, we used results of parameter distributions derived from individual fits of the much more detailed data set of Engel et al. Table 1 contains the prior values with respective $\hat{\omega}_{s}$ for all cases. All 22 population parameters fitted for Engel et al and the other experimental data were kept constant when fitting the NHL-B data. We took average values of individual parameters of Engel et al and their respective standard deviations as priors for fitting the NHL‑B patients. Two exceptions were made:

- No prior was assumed for *T_PLC_*, since all its individual estimates were well identifiable.
- The parameter *r_TPO,nor,0_* describing the ratio of steady state to initial TPO concentrations was set to 1 for the patients of NHL-B study. Indeed, *r_TPO,nor,0_* was strongly overfitted during initial attempts to estimate the parameters because no TPO data are available for the patients of the NHL-B study.

Table 1. Prior values and standard deviations for individual parameter estimates for Engel et al and NHL-B patients.

| Parameter | NHL study | | Engel et al study | | Description |
| --- | --- | --- | --- | --- | --- |
|  | $h_{k}\left( \psi_{k}^{pri} \right)$ | $\omega_{pri,k}$ | $h_{k}\left( \psi_{k}^{pri} \right)$ | $\omega_{pri,k}$ |  |
| $b_{S\_act}$ | 0.338 | 0.573 | - | - | Steepness of the regulation function of self-renewal probability p |
| $n_{CM}^{unreg}$ | 7.86 | 0.785 | - | - | Total number of cell divisions in the late TPO-unregulated CM sub-compartments |
| *r_PL,0,nor_* | 1 | 0.060 | 1 | 0.1 | Ratio of the initial PLC count to the steady state |
| *r_TPO,nor,0_* | - | - | 1 | 0.5 | Relation of the steady state TPO value to the initial value |
| *k_m,TPO_* | 0.215 | 0.755 | - | - | TPO saturation of specific elimination  (Michaelis-Menten constant) |
| $\hat{w}_{re}$ | 0.980 | 0.506 | - | - | Maximum TPO elimination rate by a single TPO receptor |
| $d_{{Osteo}_{loss}}$ | 1.25 | 0.674 | - | - | Elimination rate of dormant cells due to lack of osteoblast support |
| *pd_cyclo_* | 0.0130 | 0.292 | - | - | Toxicity of cyclophosphamide on S compartment |

## **The observation functions, weights and residual errors**

We provide *O*=14 different observation functions *g_i_* which correspond to various outcomes from different data sources:

- Platelet counts per blood liter after transfusion experiments . The observation function *g^1^* is the sum of the *n* labeled platelet age-compartments, defined by eq. (31,33):

$g^{1}\left( \vec{x}\left( t,\vec{\psi} \right) \right)=\sum_{i=1}^{n} C_{PLC,i}^{l}\left( t,\vec{\psi} \right)$.

- Platelet counts per blood liter from Harker et al data [6]. The observation function *g^2^* is the sum of the *n* circulating platelet age-compartments:

$g^{2}\left( \vec{x}\left( t,\vec{\psi} \right) \right)=\sum_{i=1}^{n} C_{PLC,i}\left( t,\vec{\psi} \right)$.

- Logarithmized platelet counts from Engel et al study and NHL-B data sets. Since it is clinically important to fit the nadir of platelets in each treatment cycle precisely, we assumed an exponential residual error, which is a constant residual error of log-transformed values. Similar to the previous case, total platelet counts are the sum of the *n* circulating platelet age-compartments:

$g^{3}\left( \vec{x}\left( t,\vec{\psi} \right) \right)=ln\left( \sum_{i=1}^{n} C_{PLC,i}\left( t,\vec{\psi} \right) \right)=ln\left( PL\left( t,\vec{\psi} \right) \right)$.

- Endogenous TPO concentrations from Engel et al data. The reason for this transformation was the fact that TPO was measured based on ELISA. This implies low reliability of low values:

$g^{4}\left( \vec{x}\left( t,\vec{\psi} \right) \right)=C_{TPO,nat}^{rel}\left( t,\vec{\psi} \right)$ .

- Concentrations of applied TPO from Harker et al [6]. The observation function g^5^ is the sum of natural TPO and pegylated TPO:

$g^{5}\left( \vec{x}\left( t,\vec{\psi} \right) \right)=C_{TPO,nat}^{rel}\left( t,\vec{\psi} \right)+C_{TPO,peg2}^{rel}\left( t,\vec{\psi} \right)$ .

- Megakaryocyte counts from Harker et al [6] is calculated as the sum of all sub-compartments of ploidies 2-128 . $g^{6}\left( \vec{x}\left( t,\vec{\psi} \right) \right)=\sum_{i=1}^{7} C_{MKC,act,P2^{i}}\left( t,\vec{\psi} \right)+\sum_{i=3}^{5} C_{MKC,dorm,P2^{i}}\left( t,\vec{\psi} \right)$.
- Percentages of megakaryocytes of ploidies 2-128 from Harker et al [6] :

$g^{l}\left( \vec{x}\left( t,\vec{\psi} \right) \right)=\left\{ \begin{matrix} \frac{C_{MKC,act,P2^{i}}\left( t,\vec{\psi} \right)+C_{MKC,dorm,P2^{i}}\left( t,\vec{\psi} \right)}{g^{6}\left( \vec{x}\left( t,\vec{\psi} \right) \right)}\begin{matrix} , & i=3, 4, 5 \end{matrix} \\ \frac{C_{MKC,act,P2^{i}}\left( t,\vec{\psi} \right)}{g^{6}\left( \vec{x}\left( t,\vec{\psi} \right) \right)}\begin{matrix} , & i=1, 2, 6, 7 \end{matrix} \end{matrix} \right.\begin{matrix} , & l=7,\cdots,13 \end{matrix}$.

- Relative osteoblast counts after chemotherapy according to Li et al [7]

$g^{14}\left( \vec{x}\left( t,\vec{\psi} \right) \right)=C_{OB}^{rel}$.

*Remark S.13.1.* For *g^3^_,_* (S.13.6) provides:

${PL}_{Data}\left( t_{i,j} \right)={PL}_{Model}\left( \vec{\psi}^{pop},\vec{\psi}_{i}^{ind}{,t}_{i,l,j} \right)\cdot e^{\varepsilon_{i,l,j}}\begin{matrix} , & i=1,\cdots,N\begin{matrix} , & l=3 \end{matrix}\begin{matrix} , & j=1,\cdots,n_{i,l} \end{matrix} \end{matrix}$. (S.13.16)

The corresponding standard deviations $\sigma_{i,l}\left( \vec{\theta}_{i,l} \right)$ can be approximated as

$\sigma_{i,l}\left( \vec{\theta}_{i,l} \right)\approx\sqrt{\frac{\sum_{j=1}^{n_{i,l}} \left( \ln\left( \frac{\mathrm{PL}_{D}\left( t_{i,j} \right)}{\mathrm{PL}_{M}\left( \vec{\psi}^{pop},\vec{\psi}_{i}^{ind}{,t}_{i,l,j} \right)} \right) \right)^{2}}{n_{i,l}}}$. (S.13.17)

Monitoring platelet counts of healthy patients without treatment [5] suggests an oscillatory response of thrombopoiesis to random noise. We derive a standard deviation from the logarithmized platelet time series of two patients resulting in similar values, namely 0.1 and 0.13, respectively. These values are rough estimates for the lower bound of *σ_i,3_*.

We used the standard deviations of measured averaged data from Harker et al [6], Hanson et al [8,9] and Li et al [7] at every time point as respective estimates of residual errors $\sigma_{i,l,j}^{bio}$:

$\sigma_{i,l,j}^{bio}\equiv s.d.\left( {data}_{i,l}^{biol}(t_{j}) \right)\begin{matrix} , & l=1,2,5,\cdots,13\begin{matrix} , & \begin{matrix} j=1,\ldots,n_{i,l}^{bio}, & j=1,\ldots,n_{i,l}^{bio}, & i=1,\ldots,L \end{matrix} \end{matrix} \end{matrix}$. (S.13.18)

Note that these equations hold for population-based parameters not for individual parameters.

We fitted $\sigma_{i,l}^{ind}$ for each individual and each observation point, assuming the same values for j=1,…, $n_{i,l}$.

$\sigma_{i,l,j}^{ind}\equiv\sigma_{i,l}^{ind}\begin{matrix} , & \begin{matrix} l=3, 4, & \begin{matrix} j=1,\ldots,n_{i,l}^{ind}, & i=1,\ldots,L^{ind} \end{matrix} \end{matrix} \end{matrix}$. (S.13.19)

Thus $\sigma_{i,l}^{ind}$ constitute $\vec{\theta}$ specified in (S.13.7-S.13.8).

Observations of different origin must be weighted to derive an overall fitness value. Most of these weights were set to 1 with the following exceptions:

1. Weights for individual NHL-B data (time series of platelet counts) ${We}_{i,3}^{ind,NHL}$ were set to 5. This was done because the NHL-B data have much sparser time series than for example those of Engel et al (i.e. less time points and lack of TPO measurements) implying a less relative importance of individual versus averaged data.
2. Labeled transfused platelets data were weighted by ${We}_{i,1}^{bio}$equal to 0.05, because otherwise, fits of other data were considerably worse.
3. Megakaryocyte counts in Harker et al. [6] were weighted by ${We}_{i,6}^{bio}$equal to 10 in order to improve the fits of these data.

## **Estimation of Fisher Information matrix - Avoidance of overfitting and estimation of precision of parameter estimates**

In order to avoid overfitting, standard errors and mutual correlations of the parameter estimates were calculated from sample-based linear approximations of the Fisher information matrix (FIM). FIM defines a multidimensional ellipsoid approximation of the negative log-likelihood in the neighborhood of a local minimum. Non-identifiability of parameters is obtained if there are singularities in the covariance matrix of parameters estimates. This is checked by the ratio of the largest eigenvalue to the smallest eigenvalue called the condition number CN [1].

A high condition number of the correlation matrix of estimates, which is a covariance matrix normalized by diagonal terms, indicates overfitting due to correlations between parameter estimates. These numbers as well as relative residual errors of fitting results serve as measures of overfitting in our study.

In order to estimate the Fisher Information Matrix (FIM) associated with a given log-likelihood we calculate its sample-based version, called the observed information (Fisher information), which is the negative of the second derivative of the log-likelihood function (Hessian matrix). We assume that residual errors of parameters θ are independent of population and individual parameters Ψ. Consequently, we calculate FIM for Ψ only.

We arrange the parameters as follows:

$\Psi=\left( \vec{\psi}^{pop},\vec{\psi}_{l,1}^{ind},\cdots\vec{\psi}_{l,N}^{ind} \right)$. (S.13.20)

For estimation, we applied a fast method described by Faller et [1] in a more general form. Briefly, it is based on linear approximations of the observation functions with respect to the parameter vector ψ in the neighborhood of the optimal value of parameters Ψ:

$\begin{matrix} FIM=\sum_{i=1}^{N} {FIM}_{i} \\ {{{FIM}_{i}=FIM}_{i}^{ind}+FIM}_{i}^{bio}+{FIM}_{i}^{pri} \end{matrix}$ . (S.13.21)

$\begin{matrix} {FIM}_{i}^{ind}\approx\sum_{l=1}^{L^{ind}} \frac{{We}_{i,l}^{ind}}{{\sigma_{i,l,j}^{ind}\left( \vec{\theta}_{i,l} \right)}^{2}}\cdot\sum_{j=1}^{n_{i,l}^{ind}} \left( \left[ \nabla_{\Psi}y_{M}^{l}\left( \vec{\psi}^{pop},\vec{\psi}_{i}^{ind},t_{i,l,j}^{ind} \right) \right]^{T}\left[ \nabla_{\Psi}y_{M}^{l}\left( \vec{\psi}^{pop},\vec{\psi}_{i}^{ind},t_{i,l,j}^{ind} \right) \right] \right) \\ \begin{matrix} {FIM}_{i}^{av}\approx\sum_{l=1}^{L^{av}} \frac{{We}_{i,l}^{bio}}{{\sigma_{i,l,j}^{bio}\left( \vec{\theta}_{i,l} \right)}^{2}}\cdot\sum_{j=1}^{n_{i,l}^{av}} \left( \left[ \nabla_{\Psi}y_{M}^{l}\left( \vec{\psi}^{pop},\vec{\psi}_{i}^{ind},t_{i,l,j}^{av} \right) \right]^{T}\left[ \nabla_{\Psi}y_{M}^{l}\left( \vec{\psi}^{pop},\vec{\psi}_{i}^{ind},t_{i,l,j}^{av} \right) \right] \right) \\ {FIM}_{i}^{pri}=\sum_{k=1}^{N_{par}^{ind}} \frac{\delta_{k}}{\omega_{pri,k}^{2}} \end{matrix} \end{matrix}$. (S.13.22)

Here the sum comprises all individual and averaged simulations for all observation functions including all data points and all patients.

According to (S.13.6, S.13.7), the gradients are given as follows:

$\begin{matrix} \begin{matrix} \nabla_{\Psi}y_{M}^{l}\left( \vec{\psi}^{pop},\vec{\psi}_{i}^{ind},t_{i,l,j}^{ind} \right)=\left( \frac{\partial y_{M}^{l}}{\partial\psi_{1}^{pop}},\cdots,\frac{\partial y_{M}^{l}}{\partial\psi_{1}^{pop}},\cdots,\delta_{i,s}\frac{\partial y_{M}^{l}}{\partial\psi_{s}^{ind}},\cdots\right) \\ \nabla_{\Psi}y_{M}^{l}\left( \vec{\psi}^{pop},\vec{\psi}_{i}^{ind},t_{i,l,j}^{av} \right)=\left( \frac{\partial y_{M}^{l}}{\partial\psi_{1}^{pop}},\cdots,\frac{\partial y_{M}^{l}}{\partial\psi_{1}^{pop}},\cdots,\delta_{i,s}\frac{\partial y_{M}^{l}}{\partial\psi_{s}^{ind}},\cdots\right) \end{matrix} \\ \delta_{i,s}=\left\{ \begin{matrix} 1\begin{matrix} , & i=s \end{matrix} \\ 0\begin{matrix} , & i\neq s \end{matrix} \end{matrix} \right. \end{matrix}$, (S.13.23)

which means that every individual simulation comprises both, averaged and individual data and depends on both, population and individual parameters.

We used the central finite differencing scheme to approximate respective gradients. Residual errors of transformed parameter estimates are the square root of diagonal elements of the inverse of FIM estimated in (S.13.23):

${\vec{s.e.}_{\Psi}\equiv\left( \vec{s.e.}_{\vec{\psi}^{pop}},\vec{s.e.}_{\vec{\psi}_{1}^{ind}},\ldots,\vec{s.e.}_{\vec{\psi}_{N}^{ind}} \right)=\mathrm{diag}\left( {FIM}^{-1} \right)}^{0.5}$. (S.13.24)

We estimate the standard errors of untransformed parameters *ϕ* from those of corresponding transformed parameters *ψ* by a stochastic simulation as follows: We sample 100,000 times from the normal distribution with mean value equal to $\psi$ and standard deviation *SE(ψ)* . The resulting empirical distribution is reversely transformed. The standard error of *ϕ* can now be estimated from the transformed sample. The relative standard error of a parameter $\varphi$ is defined as the fraction $\frac{{s.e.}_{\varphi}}{\varphi}$.

A covariance matrix of parameter estimates is then the inverse of FIM. One obtains the correlation matrix of parameter estimates by normalization of the covariance matrix to diagonal entries.

References

1. Faller D, Klingmuller U, Timmer J. Simulation Methods for Optimal Experimental Design in Systems Biology. SIMULATION. 2003; 79: 717–725. doi: 10.1177/0037549703040937.

2. Savage LJ. On Rereading R. A. Fisher. Ann. Statist. 1976; 4: 441–500. doi: 10.1214/aos/1176343456.

3. Ahmed ES, Volodin AI, Hussein AA. Robust Weighted Likelihood Estimation of Exponential Parameters. IEEE Trans. Rel. 2005; 54: 389–395. doi: 10.1109/TR.2005.853276.

4. Bailey R.A. Insurance rates with minimum bias. Proceedings of the casuality acturial society L. 1963; 4.

5. Schulthess GK von, Gessner U. Oscillating platelet counts in healthy individuals: experimental investigation and quantitative evaluation of thrombocytopoietic feedback control. Scand J Haematol. 1986; 36: 473–479.

6. Harker LA, Roskos LK, Marzec UM, Carter RA, Cherry JK, Sundell B, et al. Effects of megakaryocyte growth and development factor on platelet production, platelet life span, and platelet function in healthy human volunteers. Blood. 2000; 95: 2514–2522.

7. Li S, Zou D, Li C, Meng H, Sui W, Feng S, et al. Targeting stem cell niche can protect hematopoietic stem cells from chemotherapy and G-CSF treatment. Stem Cell Res Ther. 2015; 6: 175. doi: 10.1186/s13287-015-0164-4.

8. Hanson SR, Slichter SJ. Platelet kinetics in patients with bone marrow hypoplasia: evidence for a fixed platelet requirement. Blood. 1985; 66: 1105–1109.

9. Guerriero R, Testa U, Gabbianelli M, Mattia G, Montesoro E, Macioce G, et al. Unilineage megakaryocytic proliferation and differentiation of purified hematopoietic progenitors in serum-free liquid culture. Blood. 1995; 86: 3725–3736.
