# Supplementary material for: Modeling individual time courses of thrombopoiesis during multi-cyclic chemotherapy
Source: PLoS Comput Biol. 2019 Mar 6;15(3):e1006775. doi: 10.1371/journal.pcbi.1006775 (PMC6422316; doi:10.1371/journal.pcbi.1006775)
Supplement: S14 Appendix — (DOCX) [file pcbi.1006775.s014.docx]

# **S14 Appendix. Numerical approach for parameter estimation**

## **Algorithm used for parameter estimation**

Individual parameter estimates were obtained by likelihood methods. We used a variant of the Hooke-Jeeves method [1] for its maximization. This is a zero-order algorithm, which does not require calculation of derivatives of the fitness function to be optimized which is computationally expensive in our situation. In brief, the method relies on iterated updates of the actual fitness function values and its arguments by comparisons with fitness values in the neighborhood of the argument separately for all coordinates. Perturbation sizes at each dimension are adapted in dependence on the result of the previous step, i.e. a perturbation in the *s*-th dimension becomes larger if a better fitness value was found for this dimension in the previous step. Otherwise it is reduced in the next step, provided that it does not drop below a specified lower limit.

Algorithm stops if the last four steps did not provide a relative improvement of the fitness function of more than a specified tolerance parameter δ_tol_.

A list of individual and population parameters estimated for Engel et al data [2] is provided in Tables 1 and 2 from S15 Appendix. Parsimony assumptions are also shown in this table. Table 1 from S16 Appendix presents estimates of individual parameters fitted for patients from the NHL-B study [3–5].

References

1. Hooke R, Jeeves TA. `` Direct Search'' Solution of Numerical and Statistical Problems. J. ACM. 1961; 8: 212–229. doi: 10.1145/321062.321069.

2. Engel C, Loeffler M, Franke H, Schmitz S. Endogenous thrombopoietin serum levels during multicycle chemotherapy. Br J Haematol. 1999; 105: 832–838.

3. Wunderlich A, Kloess M, Reiser M, Rudolph C, Truemper L, Bittner S, et al. Practicability and acute haematological toxicity of 2- and 3-weekly CHOP and CHOEP chemotherapy for aggressive non-Hodgkin's lymphoma: results from the NHL-B trial of the German High-Grade Non-Hodgkin's Lymphoma Study Group (DSHNHL). Ann Oncol. 2003; 14: 881–893.

4. Pfreundschuh M, Trumper L, Kloess M, Schmits R, Feller AC, Rube C, et al. Two-weekly or 3-weekly CHOP chemotherapy with or without etoposide for the treatment of elderly patients with aggressive lymphomas: results of the NHL-B2 trial of the DSHNHL. Blood. 2004; 104: 634–641. doi: 10.1182/blood-2003-06-2095.

5. Pfreundschuh M, Trumper L, Kloess M, Schmits R, Feller AC, Rudolph C, et al. Two-weekly or 3-weekly CHOP chemotherapy with or without etoposide for the treatment of young patients with good-prognosis (normal LDH) aggressive lymphomas: results of the NHL-B1 trial of the DSHNHL. Blood. 2004; 104: 626–633. doi: 10.1182/blood-2003-06-2094.
