# Supplementary material for: Modeling individual time courses of thrombopoiesis during multi-cyclic chemotherapy
Source: PLoS Comput Biol. 2019 Mar 6;15(3):e1006775. doi: 10.1371/journal.pcbi.1006775 (PMC6422316; doi:10.1371/journal.pcbi.1006775)
Supplement: S15 Appendix — (DOCX) [file pcbi.1006775.s015.docx]

# **S15 Appendix. Parameters estimates obtained for Engel et al data**

Table 1. (Population parameters estimated on the basis of Engel et al. study): We present population parameter estimates obtained by fitting the data of Engel et al [1] together with averaged biological data [2–4]. Fitted values, relative standard errors and ω-values (see S14 Appendix) are provided. Grey background highlights relative standard errors larger than 1, i.e. poor parameter identifiability.

| Parameter | Estimate | Relative standard error | Description |
| --- | --- | --- | --- |
| ${kr}_{dorm,act}$ | 182 | 0.307 | $\frac{k_{dorm,nor}}{k_{act,nor}}$, ratio of normal activation rate of dormant stem cells to normal transition to active stem cells to dormant state |
| *k_dorm,nor_* | 0.342 | 0.676 | Activation rate of dormant stem cells |
| $b_{A_{CM}}$ | 0.618 | 0.147 | Steepness of the regulation function of total amplification of CM (*A_CM_*) |
| $p_{1}^{nor}$ | 0.102 | 0.687 | Probability of transition from MKC sub-compartment ploidies 8, 16 and 32 to the proplatelet compartment under normal (steady-state) TPO stimulation |
| $b_{{MKC}_{p,1}}$ | 0.317 | 0.259 | Steepness of the regulation function of the transition from MKC sub-compartments of ploidies 8, 16, and 32 to the proplatelet compartment |
| *T_TPO_* | 23.1 | 0.382 | Transition time of unspecific TPO elimination |
| $p_{2}$ | 0.695 | 0.109 | Probability of transition from MKC sub-compartment of ploidy 2^k^ to the next ploidy compartment |
| $T_{rev\_dorm,8}^{nor}$ | 75.0 | 0.398 | Transition times of inactive (dormant) MKC sub-compartment of ploidy 2^k^ (k=3, 4, 5) to the active sub-compartment of same ploidy under normal TPO stimulation |
| $T_{rev\_dorm,16}^{nor}$ | 477 | 0.326 |  |
| $T_{rev\_dorm,32}^{nor}$ | 328 | 0.453 |  |
| $b_{rev\_dorm,16}$ | 1.23 | 0.130 | Steepness of regulation function of transitions from inactive (dormant) MKC sub-compartment of ploidy 2^k^ (k = 4,5) to the active sub-compartment of same ploidy under normal TPO stimulation |
| $b_{rev\_dorm,32}$ | 2.34 | 0.210 |  |
| $T_{PP}$ | 3.70 | 0.547 | Transit time of proplatelet compartment |
| *npt_pcu_* | 504 | 0.275 | Produced number of platelets per ploidy of MKC |
| *k_s_* | 0.183 | 0.440 | Maximum value of Michaelis- Menten kinetics of platelet uptake by vessels |
| *q_TPO_* | 0.0121 | 0.116 | Flux rate of pegylated TPO between injection and delay compartment in case of subcutaneous injection |
| $T_{del}$ | 37.1 | 0.534 | Transition time of delayed TPO effect |
| $T_{Del,2^{5}}$ | 107 | 0.715 | Additional transition time of delayed TPO effect on activation of dormant MKC of ploidy 2^5^ |
| $b_{{MKC}_{p,64,1}}$ | 0.350 | 1.02 | Steepness of the regulation function of the transition from MKC sub-compartment of ploidy 64 to the proplatelets compartment |
| *Fr_TPO,dir_* | 0.347 | 0.405 | Fraction of TPO entering the central compartment directly due to force of injection |
| $c_{PD,Osteo}$ | 3.50E-04 | 2.29 | Ratio of chemotherapy toxicity on CM precursors and osteoblasts / osteoclasts |
| *D_ψ_* | 0.0197 | 1.62 | Delay parameter of chemotherapy effect on osteoblasts and osteoclasts |

Table 2. (Individual parameters estimated on the basis of Engel et al. study): We present individual parameter estimates obtained by fitting the data of Engel et al data [1] together with averaged biological data [2–4]. Fitted values, relative standard errors and ω-values (see S13 Appendix) are provided for each patient.

| parameters | Description | Estimates | | | | | |
| --- | --- | --- | --- | --- | --- | --- | --- |
|  |  | Pat 1 | | Pat 2 | | Pat 3 | |
|  |  | Estimate | Relative standard error | Estimate | Relative standard error | Estimate | Relative standard error |
| $d_{{Osteo}_{loss}}$ | Elimination rate of dormant cells due to lack of osteoblast support | 0.574 | 0.685 | 1.98 | 0.225 | 1.70 | 0.397 |
| $b_{S\_act}$ | Steepness of the regulation function of self-renewal probability p | 0.640 | 0.420 | 0.286 | 0.314 | 0.211 | 0.974 |
| $n_{CM}^{unreg}$ | Total number of cell divisions in the late TPO-unregulated CM sub-compartments | 7.76 | 0.0988 | 10.5 | 0.0762 | 5.32 | 0.149 |
| *T_PL_* | Transit time of platelets | 276 | 0.0443 | 260 | 0.0487 | 247 | 0.070 |
| *r_PL,0,nor_* | Ratio of the initial PLC count to the steady state | 1.03 | 0.0484 | 1.07 | 0.0739 | 1.03 | 0.072 |
| *r_TPO,nor,0_* | Relation of the steady state TPO value to the initial TPO value | 0.956 | 0.0877 | 0.167 | 0.125 | 1.86 | 0.088 |
| *k_m,TPO_* | saturation of specific TPO elimination  (Michaelis-Menten constant) | 0.512 | 0.165 | 0.151 | 0.476 | 0.129 | 0.462 |
| $\hat{w}_{re}$ | Normalized maximum TPO elimination rate by a single TPO receptor | 1.75 | 0.261 | 0.703 | 0.271 | 0.764 | 0.264 |
| *pd_cyclo_* | Toxicity of cyclophosphamide on S compartment | 0.0148 | 0.946 | 9.34E-03 | 0.947 | 0.0161 | 0.959 |

Table 3. Large positive and negative mutual correlations (absolute value exceeding 0.8) of population and individual parameters estimated by fitting the data of Engel et al data [1] together with averaged biological data [2–4]. Strong correlations of parameter estimates imply weaker parameters identifyability.

| Parameter 1 | Parameter 2 | Type of parameter 1 | Type of parameter 2 | Correlation of estimates |
| --- | --- | --- | --- | --- |
| $T_{PP}$ | $p_{1}^{nor}$ | population | population | 0.981 |
| $T_{rev\_dorm,8}^{nor}$ | $p_{2}^{nor}$ | population | population | 0.846 |
| $T_{rev\_dorm,8}^{nor}$ | $T_{rev\_dorm,16}^{nor}$ | population | population | 0.823 |
| $T_{rev\_dorm,16}^{nor}$ | $T_{rev\_dorm,32}^{nor}$ | population | population | 0.898 |
| $\hat{w}_{re}$ | *Fr_TPO,dir_* | individual, patient 1 | population | 0.827 |
| $\hat{w}_{re}$ | *T_TPO_* | individual, patient 2 | population | -0.810 |
| *pd_cyclo_* | *pd_cyclo_* | individual, patient 1 | individual, patient 2 | 0.965 |
| *pd_cyclo_* | *pd_cyclo_* | individual, patient 1 | individual, patient 3 | 0.950 |
| *pd_cyclo_* | *pd_cyclo_* | individual, patient 2 | individual, patient 3 | 0.985 |

Table 4. Distribution of MKC (%) of different ploidies at steady state as observed in Harker et al. [4] and compared with model simulation.

| Ploidy, activity | Harker et al data | Total MKC  (simulated) | Active / quiescent MKC  (simulated) |
| --- | --- | --- | --- |
| 2 | 5.83 | 7.04 | 7.04 |
| 4 | 6.61 | 7.04 | 7.04 |
| 8 | 18.94 | 17.83 | 6.35 / 11.5 |
| 16 | 47.5 | 47.5 | 3.21 / 44.3 |
| 32 | 20.5 | 20.3 | 1.63 / 18.7 |
| 64 | 0.634 | 0.276 | 0.276 |
| 128 | 0 | 0 | 0 |

References

1. Engel C, Loeffler M, Franke H, Schmitz S. Endogenous thrombopoietin serum levels during multicycle chemotherapy. Br J Haematol. 1999; 105: 832–838.

2. Li S, Zou D, Li C, Meng H, Sui W, Feng S, et al. Targeting stem cell niche can protect hematopoietic stem cells from chemotherapy and G-CSF treatment. Stem Cell Res Ther. 2015; 6: 175. doi: 10.1186/s13287-015-0164-4.

3. Hanson SR, Slichter SJ. Platelet kinetics in patients with bone marrow hypoplasia: evidence for a fixed platelet requirement. Blood. 1985; 66: 1105–1109.

4. Harker LA, Roskos LK, Marzec UM, Carter RA, Cherry JK, Sundell B, et al. Effects of megakaryocyte growth and development factor on platelet production, platelet life span, and platelet function in healthy human volunteers. Blood. 2000; 95: 2514–2522.
