# Supplementary material for: Modeling individual time courses of thrombopoiesis during multi-cyclic chemotherapy
Source: PLoS Comput Biol. 2019 Mar 6;15(3):e1006775. doi: 10.1371/journal.pcbi.1006775 (PMC6422316; doi:10.1371/journal.pcbi.1006775)
Supplement: S16 Appendix — (DOCX) [file pcbi.1006775.s016.docx]

# **S16 Appendix. Parameter estimates obtained by fitting the data of the NHL-B study [1–3]**

Table 1. Individual parameter estimates obtained by fitting the NHL-B study data together with averaged biological data [4–6]. Parameter estimation is performed using the fitting results of Engel et al. data as prior information. Ten parameters were assumed as heterogeneous between patients. Data of 135 patients were fitted. Description of these parameters can be found in Table 2 of S15 Appendix.

| ID | $d_{{Osteo}_{loss}}$ | $b_{S\_act}$ | $n_{CM}^{unreg}$ | *T_PL_* | *r_PL,0,nor_* | *k_m,TPO_* | $\hat{w}_{re}$ | *pd_cyclo_* |
| --- | --- | --- | --- | --- | --- | --- | --- | --- |
| 2 | 0.321 | 1.08 | 12.2 | 329 | 1.42 | 0.358 | 1.12 | 3.99E-03 |
| 4 | 1.44 | 0.570 | 6.73 | 247 | 0.929 | 0.112 | 0.662 | 0.0164 |
| 14 | 2.09 | 1.04 | 6.99 | 239 | 0.842 | 0.0822 | 0.592 | 0.0101 |
| 15 | 0.888 | 3.71 | 14.4 | 222 | 1.05 | 0.316 | 1.09 | 0.0197 |
| 20 | 1.32 | 0.168 | 11.9 | 279 | 1.10 | 0.279 | 0.991 | 0.0297 |
| 22 | 0.104 | 1.03 | 12.0 | 268 | 1.76 | 0.580 | 1.90 | 0.0154 |
| 37 | 1.58 | 0.407 | 3.24 | 236 | 0.912 | 0.126 | 0.768 | 0.0193 |
| 52 | 0.289 | 0.443 | 6.68 | 291 | 0.776 | 0.274 | 1.07 | 6.66E-03 |
| 69 | 2.46 | 0.582 | 5.35 | 253 | 0.810 | 0.285 | 1.10 | 4.04E-03 |
| 96 | 1.35 | 0.764 | 7.96 | 288 | 0.998 | 0.261 | 0.940 | 0.0152 |
| 114 | 2.98 | 0.294 | 12.1 | 212 | 1.05 | 0.202 | 0.895 | 9.56E-03 |
| 117 | 1.15 | 0.299 | 5.54 | 204 | 0.969 | 0.151 | 0.857 | 0.0136 |
| 145 | 0.259 | 0.744 | 5.98 | 282 | 1.04 | 0.304 | 1.10 | 3.17E-03 |
| 171 | 1.31 | 0.149 | 7.37 | 222 | 1.10 | 0.0929 | 0.669 | 7.66E-03 |
| 182 | 0.293 | 0.439 | 11.3 | 286 | 1.28 | 0.312 | 1.06 | 4.31E-03 |
| 191 | 1.39 | 1.30 | 12.1 | 193 | 1.15 | 0.246 | 1.01 | 0.0128 |
| 232 | 0.342 | 0.439 | 4.64 | 322 | 1.07 | 0.289 | 1.11 | 0.0110 |
| 247 | 3.60 | 1.12 | 14.3 | 208 | 1.03 | 0.380 | 1.45 | 8.86E-03 |
| 251 | 1.48 | 0.859 | 8.86 | 250 | 0.877 | 0.270 | 1.01 | 0.0125 |
| 277 | 0.259 | 0.695 | 10.9 | 299 | 1.17 | 0.272 | 0.946 | 5.30E-03 |
| 282 | 1.14 | 0.410 | 4.46 | 310 | 1.15 | 0.395 | 1.42 | 8.04E-03 |
| 290 | 0.202 | 0.499 | 9.41 | 253 | 0.824 | 0.252 | 0.956 | 0.0117 |
| 297 | 0.400 | 1.32 | 11.1 | 279 | 1.12 | 0.368 | 1.16 | 0.0126 |
| 301 | 0.402 | 0.540 | 9.72 | 327 | 1.06 | 0.400 | 1.29 | 5.05E-03 |
| 302 | 2.34 | 0.265 | 3.48 | 363 | 0.721 | 0.597 | 2.22 | 7.71E-03 |
| 304 | 1.87 | 0.223 | 10.6 | 265 | 0.998 | 0.0778 | 0.599 | 0.0166 |
| 313 | 0.168 | 1.03 | 9.06 | 362 | 1.07 | 0.359 | 1.15 | 0.0157 |
| 329 | 0.429 | 0.679 | 10.7 | 287 | 0.870 | 0.260 | 0.907 | 5.35E-03 |
| 341 | 0.814 | 0.263 | 9.49 | 210 | 0.799 | 0.117 | 0.695 | 9.74E-03 |
| 387 | 0.794 | 0.318 | 6.71 | 271 | 0.748 | 0.162 | 0.786 | 7.22E-03 |
| 390 | 0.243 | 0.212 | 7.69 | 245 | 1.18 | 0.174 | 0.819 | 3.70E-03 |
| 398 | 1.69 | 0.308 | 7.71 | 230 | 0.932 | 0.0811 | 0.624 | 9.25E-03 |
| 424 | 2.20 | 0.845 | 5.04 | 238 | 0.935 | 0.389 | 1.44 | 0.0177 |
| 428 | 0.743 | 0.787 | 10.6 | 306 | 0.931 | 0.299 | 0.986 | 0.0101 |
| 449 | 0.295 | 0.314 | 11.7 | 227 | 1.21 | 0.356 | 1.25 | 0.0193 |
| 465 | 0.725 | 0.430 | 8.40 | 342 | 1.33 | 0.629 | 2.11 | 8.00E-03 |
| 470 | 1.83 | 0.305 | 4.36 | 293 | 1.02 | 0.0914 | 0.666 | 0.0146 |
| 489 | 1.05 | 0.280 | 7.54 | 332 | 0.903 | 0.427 | 1.47 | 9.94E-03 |
| 517 | 0.103 | 0.993 | 8.94 | 270 | 0.926 | 0.305 | 1.12 | 9.25E-03 |
| 518 | 0.460 | 0.733 | 8.77 | 283 | 1.00 | 0.260 | 0.947 | 8.62E-03 |
| 531 | 2.93 | 0.548 | 6.76 | 193 | 0.977 | 0.0969 | 0.712 | 0.0213 |
| 550 | 0.235 | 0.800 | 2.88 | 368 | 1.09 | 0.437 | 1.46 | 4.78E-03 |
| 586 | 3.74 | 0.469 | 3.79 | 279 | 1.04 | 0.0875 | 0.660 | 0.0413 |
| 604 | 1.64 | 1.25 | 11.3 | 254 | 0.785 | 0.502 | 1.62 | 6.27E-03 |
| 608 | 1.48 | 0.558 | 10.2 | 283 | 0.859 | 0.275 | 0.965 | 8.90E-03 |
| 613 | 0.872 | 0.673 | 12.0 | 285 | 1.04 | 0.554 | 1.74 | 7.28E-03 |
| 625 | 0.681 | 0.495 | 10.0 | 331 | 1.27 | 0.417 | 1.35 | 0.0138 |
| 643 | 0.240 | 1.50 | 10.3 | 267 | 1.05 | 0.293 | 0.991 | 6.57E-03 |
| 656 | 0.366 | 0.598 | 9.72 | 280 | 1.06 | 0.444 | 1.44 | 4.96E-03 |
| 660 | 0.974 | 0.670 | 14.2 | 302 | 0.981 | 0.820 | 2.70 | 5.26E-03 |
| 670 | 1.15 | 1.21 | 11.8 | 221 | 0.898 | 0.366 | 1.35 | 7.15E-03 |
| 676 | 1.15 | 1.27 | 6.75 | 269 | 0.901 | 0.364 | 1.23 | 7.30E-03 |
| 677 | 1.06 | 0.577 | 10.1 | 209 | 1.08 | 0.378 | 1.40 | 9.62E-03 |
| 688 | 2.08 | 1.28 | 9.66 | 259 | 0.869 | 0.440 | 1.46 | 7.63E-03 |
| 699 | 0.163 | 1.15 | 10.0 | 490 | 1.12 | 0.320 | 1.13 | 0.0248 |
| 706 | 3.51 | 0.871 | 5.29 | 267 | 0.802 | 0.414 | 1.44 | 8.79E-03 |
| 707 | 0.223 | 0.496 | 10.5 | 240 | 0.780 | 0.317 | 1.11 | 9.27E-03 |
| 712 | 1.36 | 0.399 | 3.30 | 292 | 0.983 | 0.235 | 0.986 | 0.0146 |
| 729 | 0.0978 | 0.950 | 5.23 | 319 | 1.09 | 0.0942 | 0.596 | 0.0192 |
| 742 | 5.66 | 1.88 | 9.66 | 224 | 0.950 | 0.126 | 0.676 | 0.0113 |
| 760 | 1.55 | 0.457 | 8.86 | 265 | 1.21 | 0.610 | 2.14 | 4.85E-03 |
| 763 | 0.409 | 1.03 | 9.90 | 317 | 1.06 | 0.283 | 0.941 | 0.0127 |
| 765 | 1.16 | 0.492 | 5.76 | 248 | 0.797 | 0.345 | 1.29 | 0.0121 |
| 774 | 0.289 | 0.346 | 8.61 | 349 | 0.903 | 0.333 | 1.15 | 6.12E-03 |
| 783 | 0.349 | 0.338 | 8.01 | 263 | 0.787 | 0.130 | 0.704 | 0.0159 |
| 799 | 0.612 | 0.617 | 10.5 | 237 | 0.653 | 0.181 | 0.799 | 6.03E-03 |
| 801 | 0.0998 | 1.29 | 10.0 | 317 | 1.58 | 0.298 | 0.985 | 0.0174 |
| 804 | 0.168 | 0.356 | 11.4 | 279 | 0.915 | 0.418 | 1.39 | 3.71E-03 |
| 806 | 1.37 | 0.536 | 5.81 | 273 | 0.991 | 0.719 | 2.49 | 4.05E-03 |
| 814 | 1.79 | 0.890 | 7.64 | 205 | 0.727 | 0.0253 | 0.490 | 0.0122 |
| 833 | 2.16 | 1.29 | 13.5 | 237 | 1.11 | 0.138 | 0.648 | 0.0162 |
| 836 | 0.227 | 0.684 | 10.3 | 341 | 0.889 | 0.349 | 1.13 | 0.0120 |
| 843 | 0.228 | 1.01 | 9.18 | 407 | 1.03 | 0.349 | 1.15 | 0.0159 |
| 869 | 1.11 | 0.416 | 9.25 | 321 | 0.959 | 0.418 | 1.38 | 3.47E-03 |
| 881 | 0.142 | 1.30 | 9.10 | 348 | 1.02 | 0.340 | 1.09 | 4.04E-03 |
| 891 | 0.362 | 0.847 | 5.62 | 311 | 1.38 | 0.263 | 0.965 | 0.0173 |
| 903 | 1.80 | 0.476 | 4.71 | 345 | 0.792 | 0.382 | 1.34 | 5.46E-03 |
| 912 | 0.594 | 0.422 | 6.59 | 342 | 1.04 | 0.377 | 1.30 | 5.90E-03 |
| 929 | 0.580 | 0.501 | 9.51 | 363 | 0.811 | 0.447 | 1.44 | 0.0167 |
| 935 | 0.919 | 0.545 | 10.8 | 247 | 1.09 | 0.374 | 1.30 | 0.0223 |
| 939 | 0.212 | 0.278 | 8.17 | 349 | 1.02 | 0.470 | 1.57 | 2.61E-03 |
| 944 | 3.08 | 0.107 | 9.73 | 191 | 0.806 | 0.0564 | 0.602 | 9.74E-03 |
| 955 | 0.535 | 0.0767 | 6.94 | 184 | 1.19 | 0.0764 | 0.687 | 0.0215 |
| 970 | 2.30 | 0.149 | 8.48 | 231 | 0.991 | 0.119 | 0.691 | 0.0147 |
| 983 | 0.179 | 0.819 | 7.73 | 397 | 0.948 | 0.472 | 1.53 | 7.88E-03 |
| 997 | 1.90 | 0.359 | 9.87 | 234 | 0.936 | 0.128 | 0.679 | 0.0116 |
| 998 | 0.118 | 0.659 | 9.52 | 258 | 1.28 | 0.202 | 0.819 | 0.0114 |
| 999 | 2.29 | 0.586 | 5.61 | 198 | 0.841 | 0.138 | 0.820 | 0.0111 |
| 1014 | 4.51 | 0.522 | 12.6 | 191 | 1.07 | 0.234 | 1.00 | 0.0140 |
| 1017 | 2.10 | 0.585 | 4.90 | 243 | 0.867 | 0.178 | 0.854 | 0.0132 |
| 1023 | 0.615 | 1.55 | 9.14 | 273 | 0.841 | 0.205 | 0.789 | 8.81E-03 |
| 1029 | 0.0955 | 1.90 | 12.2 | 276 | 1.64 | 0.278 | 0.921 | 7.49E-03 |
| 1031 | 0.265 | 1.60 | 13.8 | 274 | 0.859 | 0.504 | 1.57 | 6.35E-03 |
| 1032 | 0.418 | 0.684 | 9.43 | 342 | 0.966 | 0.397 | 1.27 | 9.25E-03 |
| 1052 | 0.284 | 0.352 | 11.3 | 263 | 0.963 | 0.837 | 2.89 | 7.72E-03 |
| 1119 | 2.45 | 0.600 | 8.69 | 256 | 0.968 | 0.142 | 0.709 | 0.0198 |
| 1149 | 0.753 | 1.42 | 12.4 | 233 | 0.784 | 0.531 | 1.76 | 8.19E-03 |
| 1199 | 1.30 | 0.599 | 6.12 | 295 | 1.05 | 0.203 | 0.856 | 5.46E-03 |
| 1224 | 1.08 | 0.583 | 12.1 | 255 | 0.899 | 0.452 | 1.49 | 8.66E-03 |
| 1228 | 0.209 | 1.10 | 10.3 | 266 | 0.963 | 0.492 | 1.57 | 6.34E-03 |
| 1240 | 0.424 | 0.863 | 9.97 | 235 | 0.881 | 0.264 | 0.996 | 0.0116 |
| 1253 | 0.304 | 0.691 | 11.4 | 349 | 1.09 | 0.442 | 1.37 | 0.0101 |
| 1261 | 1.59 | 0.648 | 5.13 | 199 | 0.797 | 0.146 | 0.823 | 0.0134 |
| 1289 | 0.347 | 0.287 | 8.26 | 308 | 0.902 | 0.371 | 1.29 | 7.65E-03 |
| 1303 | 1.20 | 0.867 | 10.7 | 198 | 0.855 | 0.269 | 1.07 | 0.0228 |
| 1323 | 1.73 | 0.690 | 7.14 | 289 | 0.928 | 0.145 | 0.697 | 9.38E-03 |
| 1330 | 1.05 | 0.853 | 9.84 | 257 | 0.908 | 0.165 | 0.708 | 0.0177 |
| 1349 | 0.282 | 0.443 | 9.83 | 305 | 0.659 | 0.522 | 1.62 | 6.16E-03 |
| 1358 | 0.170 | 0.921 | 12.1 | 285 | 1.17 | 0.328 | 1.07 | 9.25E-03 |
| 1367 | 0.179 | 0.959 | 9.61 | 312 | 1.26 | 0.353 | 1.14 | 0.0109 |
| 1375 | 0.435 | 0.501 | 6.12 | 305 | 0.855 | 0.253 | 0.988 | 6.63E-03 |
| 1378 | 0.223 | 1.34 | 12.2 | 293 | 1.53 | 0.429 | 1.31 | 5.74E-03 |
| 1390 | 1.99 | 0.967 | 8.61 | 246 | 0.959 | 0.221 | 0.870 | 0.0112 |
| 1399 | 2.45 | 0.317 | 6.14 | 229 | 1.04 | 0.177 | 0.879 | 0.0135 |
| 1421 | 0.979 | 1.39 | 12.9 | 216 | 0.944 | 0.124 | 0.666 | 9.11E-03 |
| 1428 | 0.334 | 0.673 | 9.76 | 347 | 0.828 | 0.414 | 1.33 | 5.70E-03 |
| 1435 | 1.46 | 1.91 | 12.7 | 246 | 0.810 | 0.485 | 1.59 | 8.00E-03 |
| 1440 | 2.20 | 1.74 | 13.3 | 214 | 0.709 | 0.212 | 0.879 | 9.93E-03 |
| 1463 | 0.668 | 0.334 | 6.32 | 197 | 1.42 | 0.195 | 0.960 | 0.0588 |
| 1471 | 0.543 | 0.465 | 6.68 | 318 | 0.992 | 0.182 | 0.825 | 0.0106 |
| 1485 | 2.44 | 0.723 | 11.0 | 237 | 1.03 | 0.124 | 0.663 | 0.0227 |
| 1488 | 3.64 | 0.599 | 8.41 | 320 | 0.928 | 0.840 | 3.18 | 0.0135 |
| 1527 | 4.10 | 0.303 | 6.56 | 225 | 0.766 | 0.0620 | 0.611 | 9.86E-03 |
| 1534 | 0.595 | 0.443 | 6.00 | 202 | 0.902 | 0.101 | 0.708 | 0.0146 |
| 1548 | 1.40 | 0.269 | 9.18 | 247 | 0.941 | 0.155 | 0.748 | 0.0166 |
| 1555 | 2.54 | 0.662 | 5.53 | 308 | 0.960 | 0.420 | 1.61 | 0.0343 |
| 1600 | 2.08 | 0.583 | 6.02 | 255 | 0.784 | 0.158 | 0.771 | 5.80E-03 |
| 1641 | 3.37 | 0.967 | 5.28 | 242 | 0.975 | 0.204 | 0.861 | 0.0152 |
| 1647 | 0.235 | 0.610 | 7.02 | 288 | 1.37 | 0.278 | 1.01 | 4.52E-03 |
| 1655 | 2.21 | 0.917 | 6.31 | 316 | 1.07 | 0.806 | 2.81 | 6.28E-03 |
| 1660 | 1.17 | 0.828 | 9.81 | 237 | 1.01 | 0.194 | 0.804 | 8.09E-03 |
| 1678 | 1.67 | 0.151 | 9.30 | 199 | 0.865 | 0.106 | 0.712 | 0.0102 |
| 1690 | 1.68 | 0.0891 | 9.58 | 254 | 1.12 | 0.255 | 0.991 | 5.95E-03 |
| 1693 | 0.983 | 0.411 | 8.35 | 359 | 0.874 | 0.430 | 1.45 | 0.0125 |
| 1696 | 0.220 | 1.06 | 10.0 | 330 | 1.10 | 0.262 | 0.901 | 0.0126 |

Table 2. Relative residual standard errors of individual parameter estimates presented in the Table 1 of this appendix as well as estimation of residual errors σ of fitted logarithmized platelet counts for each subject (last column), see also S13 Appendix. Description of these parameters can be found in Table 2 in S15 Appendix.

| ID | $d_{{Osteo}_{loss}}$ | $b_{S\_act}$ | $n_{CM}^{unreg}$ | *T_PL_* | *r_PL,0,nor_* | *k_m,TPO_* | $\hat{w}_{re}$ | *pd_cyclo_* | σ |
| --- | --- | --- | --- | --- | --- | --- | --- | --- | --- |
| 2 | 1.05 | 0.268 | 0.0696 | 0.0441 | 0.394 | 0.0371 | 0.227 | 0.194 | 0.204 |
| 4 | 0.187 | 0.195 | 0.0421 | 0.0261 | 1.46 | 0.0571 | 0.154 | 0.201 | 0.356 |
| 14 | 0.191 | 0.151 | 0.0810 | 0.0288 | 2.01 | 0.0547 | 0.147 | 0.208 | 0.261 |
| 15 | 0.308 | 0.124 | 0.0305 | 0.0277 | 0.440 | 0.0484 | 0.214 | 0.190 | 0.374 |
| 20 | 0.170 | 0.407 | 0.0822 | 0.0341 | 0.175 | 0.0573 | 0.103 | 0.145 | 0.413 |
| 22 | 1.03 | 0.249 | 0.0509 | 0.0380 | 0.307 | 0.0370 | 0.223 | 0.222 | 0.277 |
| 37 | 0.239 | 0.312 | 0.129 | 0.0389 | 1.30 | 0.0621 | 0.182 | 0.220 | 0.476 |
| 52 | 0.893 | 0.356 | 0.129 | 0.0453 | 0.457 | 0.0521 | 0.232 | 0.208 | 0.314 |
| 69 | 0.293 | 0.217 | 0.0791 | 0.0450 | 0.471 | 0.0198 | 0.206 | 0.211 | 0.0877 |
| 96 | 0.316 | 0.229 | 0.0609 | 0.0419 | 0.545 | 0.0458 | 0.223 | 0.237 | 0.283 |
| 114 | 0.266 | 0.287 | 0.0613 | 0.0291 | 0.418 | 0.0563 | 0.140 | 0.169 | 0.568 |
| 117 | 0.285 | 0.388 | 0.0967 | 0.0343 | 0.969 | 0.0547 | 0.179 | 0.188 | 0.395 |
| 145 | 0.974 | 0.310 | 0.116 | 0.0517 | 0.566 | 0.0175 | 0.236 | 0.209 | 0.116 |
| 171 | 0.302 | 0.431 | 0.0859 | 0.0459 | 1.31 | 0.0322 | 0.180 | 0.196 | 0.199 |
| 182 | 0.820 | 0.275 | 0.0514 | 0.0342 | 0.476 | 0.0312 | 0.219 | 0.192 | 0.147 |
| 191 | 0.320 | 0.186 | 0.0710 | 0.0389 | 0.415 | 0.0597 | 0.179 | 0.224 | 0.756 |
| 232 | 0.608 | 0.424 | 0.202 | 0.0516 | 0.607 | 0.0393 | 0.233 | 0.242 | 0.220 |
| 247 | 0.236 | 0.118 | 0.0301 | 0.0222 | 0.221 | 0.0506 | 0.128 | 0.213 | 0.327 |
| 251 | 0.366 | 0.270 | 0.188 | 0.0464 | 0.495 | 0.0616 | 0.224 | 0.249 | 0.573 |
| 277 | 0.972 | 0.302 | 0.0569 | 0.0347 | 0.516 | 0.0371 | 0.220 | 0.222 | 0.149 |
| 282 | 0.446 | 0.473 | 0.205 | 0.0497 | 0.458 | 0.0361 | 0.245 | 0.240 | 0.190 |
| 290 | 0.717 | 0.283 | 0.0413 | 0.0274 | 0.531 | 0.0364 | 0.219 | 0.186 | 0.101 |
| 297 | 0.446 | 0.179 | 0.0647 | 0.0292 | 0.407 | 0.0453 | 0.232 | 0.200 | 0.234 |
| 301 | 0.374 | 0.158 | 0.0434 | 0.0298 | 0.386 | 0.0275 | 0.210 | 0.191 | 0.148 |
| 302 | 0.227 | 0.344 | 0.176 | 0.0285 | 0.0816 | 0.0427 | 0.0938 | 0.236 | 0.193 |
| 304 | 0.297 | 0.364 | 0.0659 | 0.0375 | 1.97 | 0.0388 | 0.158 | 0.225 | 0.229 |
| 313 | 1.00 | 0.330 | 0.0909 | 0.0561 | 0.430 | 0.0436 | 0.234 | 0.247 | 0.333 |
| 329 | 0.405 | 0.278 | 0.0564 | 0.0298 | 0.486 | 0.0434 | 0.220 | 0.217 | 0.149 |
| 341 | 0.409 | 0.399 | 0.0774 | 0.0345 | 0.948 | 0.0537 | 0.201 | 0.221 | 0.395 |
| 387 | 0.425 | 0.413 | 0.0928 | 0.0439 | 0.939 | 0.0329 | 0.202 | 0.228 | 0.189 |
| 390 | 0.895 | 0.455 | 0.0860 | 0.0448 | 0.913 | 0.0263 | 0.201 | 0.170 | 0.157 |
| 398 | 0.260 | 0.318 | 0.0715 | 0.0330 | 2.53 | 0.0498 | 0.135 | 0.210 | 0.361 |
| 424 | 0.202 | 0.162 | 0.0994 | 0.0264 | 0.234 | 0.0565 | 0.148 | 0.236 | 0.262 |
| 428 | 0.467 | 0.337 | 0.0792 | 0.0428 | 0.468 | 0.0561 | 0.225 | 0.238 | 0.435 |
| 449 | 0.471 | 0.385 | 0.0589 | 0.0283 | 0.409 | 0.0487 | 0.210 | 0.176 | 0.264 |
| 465 | 0.549 | 0.355 | 0.0702 | 0.0343 | 0.323 | 0.0349 | 0.242 | 0.231 | 0.154 |
| 470 | 0.269 | 0.364 | 0.106 | 0.0408 | 2.06 | 0.0361 | 0.154 | 0.228 | 0.191 |
| 489 | 0.377 | 0.561 | 0.102 | 0.0407 | 0.409 | 0.0391 | 0.237 | 0.243 | 0.246 |
| 517 | 0.608 | 0.158 | 0.0417 | 0.0274 | 0.354 | 0.0322 | 0.210 | 0.163 | 0.135 |
| 518 | 0.553 | 0.346 | 0.0593 | 0.0379 | 0.528 | 0.0413 | 0.224 | 0.228 | 0.183 |
| 531 | 0.226 | 0.155 | 0.0954 | 0.0331 | 1.55 | 0.0629 | 0.136 | 0.227 | 0.635 |
| 550 | 0.946 | 0.257 | 0.259 | 0.0470 | 0.414 | 0.0174 | 0.247 | 0.234 | 0.163 |
| 586 | 0.0770 | 0.147 | 0.135 | 0.0220 | 1.49 | 0.0641 | 0.136 | 0.184 | 0.401 |
| 604 | 0.237 | 0.150 | 0.0493 | 0.0258 | 0.145 | 0.0451 | 0.134 | 0.220 | 0.182 |
| 608 | 0.347 | 0.365 | 0.0958 | 0.0553 | 0.540 | 0.0636 | 0.228 | 0.239 | 0.653 |
| 613 | 0.382 | 0.253 | 0.0608 | 0.0277 | 0.222 | 0.0461 | 0.193 | 0.202 | 0.202 |
| 625 | 0.410 | 0.295 | 0.0547 | 0.0256 | 0.361 | 0.0368 | 0.220 | 0.218 | 0.153 |
| 643 | 0.761 | 0.136 | 0.0462 | 0.0304 | 0.498 | 0.0334 | 0.230 | 0.199 | 0.257 |
| 656 | 0.596 | 0.263 | 0.0558 | 0.0296 | 0.370 | 0.0364 | 0.231 | 0.201 | 0.175 |
| 660 | 0.240 | 0.147 | 0.0550 | 0.0181 | 0.102 | 0.0450 | 0.116 | 0.223 | 0.213 |
| 670 | 0.272 | 0.153 | 0.0764 | 0.0367 | 0.230 | 0.0491 | 0.173 | 0.198 | 0.254 |
| 676 | 0.239 | 0.168 | 0.149 | 0.0314 | 0.419 | 0.0446 | 0.231 | 0.200 | 0.230 |
| 677 | 0.308 | 0.257 | 0.0441 | 0.0262 | 0.312 | 0.0466 | 0.166 | 0.173 | 0.277 |
| 688 | 0.237 | 0.196 | 0.0739 | 0.0301 | 0.207 | 0.0501 | 0.150 | 0.233 | 0.272 |
| 699 | 0.798 | 0.333 | 0.135 | 0.0660 | 0.545 | 0.0469 | 0.228 | 0.243 | 0.104 |
| 706 | 0.203 | 0.162 | 0.157 | 0.0332 | 0.239 | 0.0592 | 0.152 | 0.247 | 0.362 |
| 707 | 0.454 | 0.301 | 0.101 | 0.0366 | 0.431 | 0.0500 | 0.220 | 0.199 | 0.275 |
| 712 | 0.249 | 0.248 | 0.128 | 0.0374 | 0.584 | 0.0284 | 0.198 | 0.211 | 0.103 |
| 729 | 0.853 | 0.190 | 0.102 | 0.0322 | 1.91 | 0.0314 | 0.166 | 0.162 | 0.165 |
| 742 | 0.187 | 0.0727 | 0.0334 | 0.0299 | 0.470 | 0.0475 | 0.127 | 0.180 | 0.383 |
| 760 | 0.264 | 0.172 | 0.0419 | 0.0177 | 0.0771 | 0.0226 | 0.0890 | 0.174 | 0.113 |
| 763 | 0.553 | 0.295 | 0.126 | 0.0456 | 0.484 | 0.0507 | 0.225 | 0.228 | 0.208 |
| 765 | 0.345 | 0.280 | 0.0704 | 0.0430 | 0.394 | 0.0327 | 0.208 | 0.212 | 0.206 |
| 774 | 0.527 | 0.404 | 0.0921 | 0.0290 | 0.424 | 0.0446 | 0.223 | 0.215 | 0.230 |
| 783 | 0.512 | 0.378 | 0.0954 | 0.0347 | 0.911 | 0.0430 | 0.198 | 0.241 | 0.211 |
| 799 | 0.415 | 0.298 | 0.0627 | 0.0371 | 0.714 | 0.0444 | 0.214 | 0.215 | 0.223 |
| 801 | 1.05 | 0.257 | 0.0649 | 0.0406 | 0.477 | 0.0386 | 0.223 | 0.234 | 0.293 |
| 804 | 0.955 | 0.298 | 0.0771 | 0.0326 | 0.397 | 0.0364 | 0.230 | 0.203 | 0.230 |
| 806 | 0.415 | 0.303 | 0.0884 | 0.0559 | 0.322 | 0.0269 | 0.263 | 0.216 | 0.135 |
| 814 | 0.229 | 0.124 | 0.121 | 0.0248 | 3.10 | 0.0475 | 0.106 | 0.192 | 0.233 |
| 833 | 0.194 | 0.119 | 0.0280 | 0.0186 | 0.631 | 0.0506 | 0.132 | 0.206 | 0.336 |
| 836 | 0.973 | 0.380 | 0.127 | 0.0483 | 0.434 | 0.0486 | 0.235 | 0.211 | 0.186 |
| 843 | 0.785 | 0.312 | 0.106 | 0.0532 | 0.474 | 0.0447 | 0.233 | 0.189 | 0.219 |
| 869 | 0.632 | 0.437 | 0.154 | 0.0590 | 0.460 | 0.0458 | 0.235 | 0.218 | 0.210 |
| 881 | 1.05 | 0.295 | 0.142 | 0.0549 | 0.459 | 0.0517 | 0.231 | 0.218 | 0.227 |
| 891 | 0.713 | 0.255 | 0.127 | 0.0446 | 0.602 | 0.0355 | 0.228 | 0.187 | 0.302 |
| 903 | 0.335 | 0.278 | 0.153 | 0.0526 | 0.401 | 0.0416 | 0.229 | 0.217 | 0.232 |
| 912 | 0.544 | 0.453 | 0.120 | 0.0456 | 0.478 | 0.0333 | 0.233 | 0.226 | 0.156 |
| 929 | 0.480 | 0.358 | 0.130 | 0.0458 | 0.380 | 0.0430 | 0.239 | 0.229 | 0.163 |
| 935 | 0.193 | 0.236 | 0.0438 | 0.0251 | 0.176 | 0.0512 | 0.109 | 0.200 | 0.365 |
| 939 | 1.03 | 0.481 | 0.0885 | 0.0408 | 0.368 | 0.0276 | 0.240 | 0.203 | 0.116 |
| 944 | 0.214 | 0.511 | 0.0581 | 0.0416 | 2.87 | 0.0622 | 0.110 | 0.179 | 0.733 |
| 955 | 0.282 | 0.589 | 0.0618 | 0.0175 | 2.19 | 0.0293 | 0.151 | 0.119 | 0.139 |
| 970 | 0.291 | 0.534 | 0.182 | 0.0607 | 2.22 | 0.0653 | 0.157 | 0.242 | 1.162 |
| 983 | 0.954 | 0.331 | 0.163 | 0.0602 | 0.402 | 0.0557 | 0.250 | 0.213 | 0.289 |
| 997 | 0.289 | 0.265 | 0.107 | 0.0412 | 1.49 | 0.0522 | 0.152 | 0.194 | 0.322 |
| 998 | 0.997 | 0.251 | 0.0523 | 0.0331 | 0.651 | 0.0352 | 0.214 | 0.201 | 0.178 |
| 999 | 0.224 | 0.200 | 0.207 | 0.0327 | 0.698 | 0.0570 | 0.161 | 0.232 | 0.338 |
| 1014 | 0.261 | 0.137 | 0.0829 | 0.0445 | 0.284 | 0.0627 | 0.136 | 0.242 | 0.875 |
| 1017 | 0.295 | 0.330 | 0.115 | 0.0454 | 0.779 | 0.0625 | 0.219 | 0.248 | 0.471 |
| 1023 | 0.495 | 0.166 | 0.0557 | 0.0360 | 0.614 | 0.0467 | 0.221 | 0.219 | 0.319 |
| 1029 | 0.999 | 0.142 | 0.0454 | 0.0288 | 0.466 | 0.0323 | 0.211 | 0.187 | 0.259 |
| 1031 | 0.527 | 0.243 | 0.0257 | 0.0287 | 0.299 | 0.0397 | 0.234 | 0.191 | 0.231 |
| 1032 | 0.576 | 0.360 | 0.102 | 0.0475 | 0.426 | 0.0384 | 0.229 | 0.231 | 0.165 |
| 1052 | 0.531 | 0.222 | 0.0608 | 0.0305 | 0.271 | 0.0383 | 0.228 | 0.205 | 0.196 |
| 1119 | 0.268 | 0.235 | 0.0716 | 0.0414 | 0.887 | 0.0498 | 0.183 | 0.237 | 0.243 |
| 1149 | 0.346 | 0.164 | 0.0531 | 0.0305 | 0.305 | 0.0447 | 0.224 | 0.203 | 0.257 |
| 1199 | 0.296 | 0.258 | 0.0673 | 0.0457 | 0.703 | 0.0223 | 0.213 | 0.207 | 0.111 |
| 1224 | 0.308 | 0.202 | 0.0687 | 0.0275 | 0.249 | 0.0440 | 0.172 | 0.206 | 0.192 |
| 1228 | 0.639 | 0.213 | 0.0499 | 0.0304 | 0.362 | 0.0407 | 0.237 | 0.201 | 0.159 |
| 1240 | 0.897 | 0.265 | 0.0749 | 0.0517 | 0.601 | 0.0426 | 0.232 | 0.236 | 0.340 |
| 1253 | 0.834 | 0.307 | 0.0813 | 0.0376 | 0.387 | 0.0410 | 0.230 | 0.246 | 0.244 |
| 1261 | 0.154 | 0.151 | 0.0416 | 0.0172 | 0.611 | 0.0421 | 0.146 | 0.153 | 0.154 |
| 1289 | 0.472 | 0.439 | 0.0750 | 0.0371 | 0.413 | 0.0428 | 0.225 | 0.216 | 0.207 |
| 1303 | 0.199 | 0.172 | 0.0946 | 0.0283 | 0.279 | 0.0549 | 0.137 | 0.205 | 0.283 |
| 1323 | 0.337 | 0.296 | 0.128 | 0.0538 | 1.00 | 0.0595 | 0.203 | 0.222 | 0.295 |
| 1330 | 0.280 | 0.222 | 0.0743 | 0.0407 | 0.777 | 0.0570 | 0.209 | 0.226 | 0.282 |
| 1349 | 0.454 | 0.320 | 0.0698 | 0.0393 | 0.306 | 0.0403 | 0.246 | 0.228 | 0.241 |
| 1358 | 0.843 | 0.226 | 0.0560 | 0.0307 | 0.450 | 0.0417 | 0.224 | 0.214 | 0.252 |
| 1367 | 0.790 | 0.223 | 0.0654 | 0.0326 | 0.393 | 0.0402 | 0.210 | 0.192 | 0.269 |
| 1375 | 0.604 | 0.389 | 0.118 | 0.0480 | 0.615 | 0.0511 | 0.226 | 0.235 | 0.342 |
| 1378 | 0.792 | 0.149 | 0.0389 | 0.0218 | 0.314 | 0.0367 | 0.227 | 0.181 | 0.144 |
| 1390 | 0.221 | 0.215 | 0.162 | 0.0337 | 0.430 | 0.0603 | 0.157 | 0.236 | 0.376 |
| 1399 | 0.283 | 0.483 | 0.100 | 0.0474 | 0.824 | 0.0396 | 0.187 | 0.225 | 0.211 |
| 1421 | 0.341 | 0.169 | 0.0690 | 0.0363 | 1.96 | 0.0534 | 0.178 | 0.216 | 0.369 |
| 1428 | 0.949 | 0.359 | 0.104 | 0.0506 | 0.425 | 0.0458 | 0.234 | 0.234 | 0.274 |
| 1435 | 0.261 | 0.134 | 0.0694 | 0.0314 | 0.238 | 0.0469 | 0.170 | 0.213 | 0.274 |
| 1440 | 0.212 | 0.0935 | 0.0583 | 0.0227 | 0.293 | 0.0489 | 0.135 | 0.196 | 0.333 |
| 1463 | 0.298 | 0.385 | 0.0703 | 0.0275 | 0.870 | 0.0536 | 0.201 | 0.167 | 0.298 |
| 1471 | 0.430 | 0.233 | 0.0795 | 0.0416 | 0.682 | 0.0318 | 0.216 | 0.219 | 0.164 |
| 1485 | 0.213 | 0.224 | 0.0859 | 0.0293 | 0.982 | 0.0614 | 0.162 | 0.237 | 0.467 |
| 1488 | 0.152 | 0.111 | 0.0780 | 0.0158 | 0.128 | 0.0571 | 0.132 | 0.215 | 0.142 |
| 1527 | 0.252 | 0.228 | 0.101 | 0.0500 | 3.40 | 0.0627 | 0.112 | 0.197 | 0.625 |
| 1534 | 0.383 | 0.357 | 0.100 | 0.0361 | 1.91 | 0.0460 | 0.170 | 0.213 | 0.187 |
| 1548 | 0.221 | 0.286 | 0.0844 | 0.0518 | 0.749 | 0.0603 | 0.160 | 0.169 | 0.380 |
| 1555 | 0.161 | 0.135 | 0.0510 | 0.0232 | 0.133 | 0.0572 | 0.101 | 0.188 | 0.336 |
| 1600 | 0.190 | 0.194 | 0.0591 | 0.0285 | 0.892 | 0.0322 | 0.170 | 0.182 | 0.164 |
| 1641 | 0.150 | 0.110 | 0.156 | 0.0264 | 0.489 | 0.0611 | 0.139 | 0.241 | 0.325 |
| 1647 | 0.633 | 0.271 | 0.0595 | 0.0288 | 0.508 | 0.0321 | 0.218 | 0.176 | 0.123 |
| 1655 | 0.140 | 0.122 | 0.0454 | 0.0130 | 0.0777 | 0.0345 | 0.0884 | 0.165 | 0.081 |
| 1660 | 0.285 | 0.234 | 0.0544 | 0.0418 | 0.669 | 0.0419 | 0.226 | 0.208 | 0.193 |
| 1678 | 0.307 | 0.394 | 0.132 | 0.0360 | 1.69 | 0.0555 | 0.151 | 0.197 | 0.463 |
| 1690 | 0.237 | 0.603 | 0.0797 | 0.0437 | 0.402 | 0.0525 | 0.157 | 0.172 | 0.497 |
| 1693 | 0.510 | 0.472 | 0.161 | 0.0646 | 0.449 | 0.0561 | 0.249 | 0.254 | 0.406 |
| 1696 | 0.859 | 0.321 | 0.108 | 0.0522 | 0.513 | 0.0496 | 0.223 | 0.232 | 0.197 |

**Fig 1-15.** Fits of platelet dynamics of the 135 patients from NHL-B study [3] receiving either CHO(E)P-14 or CHO(E)P-21 treatment. Strong inter-individual and inter-cycle variability of response is observed. Most of the time series are well fitted by the model using individual therapy schedules and the individual parameter estimates presented in the Table 1 of this appendix.

**Fig 1.**

**Fig 2.**

**Fig 3.**

**Fig 4.**

**Fig 5.**

**Fig 6.**

**Fig 7.**

**Fig 8.**

**Fig 9.**

**Fig 10.**

**Fig 11.**

**Fig 12.**

**Fig 13.**

**Fig 14.**

**Fig 15.**

References

1. Pfreundschuh M, Trumper L, Kloess M, Schmits R, Feller AC, Rudolph C, et al. Two-weekly or 3-weekly CHOP chemotherapy with or without etoposide for the treatment of young patients with good-prognosis (normal LDH) aggressive lymphomas: results of the NHL-B1 trial of the DSHNHL. Blood. 2004; 104: 626–633. doi: 10.1182/blood-2003-06-2094.

2. Pfreundschuh M, Trumper L, Kloess M, Schmits R, Feller AC, Rube C, et al. Two-weekly or 3-weekly CHOP chemotherapy with or without etoposide for the treatment of elderly patients with aggressive lymphomas: results of the NHL-B2 trial of the DSHNHL. Blood. 2004; 104: 634–641. doi: 10.1182/blood-2003-06-2095.

3. Wunderlich A, Kloess M, Reiser M, Rudolph C, Truemper L, Bittner S, et al. Practicability and acute haematological toxicity of 2- and 3-weekly CHOP and CHOEP chemotherapy for aggressive non-Hodgkin's lymphoma: results from the NHL-B trial of the German High-Grade Non-Hodgkin's Lymphoma Study Group (DSHNHL). Ann Oncol. 2003; 14: 881–893.

4. Li S, Zou D, Li C, Meng H, Sui W, Feng S, et al. Targeting stem cell niche can protect hematopoietic stem cells from chemotherapy and G-CSF treatment. Stem Cell Res Ther. 2015; 6: 175. doi: 10.1186/s13287-015-0164-4.

5. Hanson SR, Slichter SJ. Platelet kinetics in patients with bone marrow hypoplasia: evidence for a fixed platelet requirement. Blood. 1985; 66: 1105–1109.

6. Harker LA, Roskos LK, Marzec UM, Carter RA, Cherry JK, Sundell B, et al. Effects of megakaryocyte growth and development factor on platelet production, platelet life span, and platelet function in healthy human volunteers. Blood. 2000; 95: 2514–2522.
