# Supplementary material for: Modeling individual time courses of thrombopoiesis during multi-cyclic chemotherapy
Source: PLoS Comput Biol. 2019 Mar 6;15(3):e1006775. doi: 10.1371/journal.pcbi.1006775 (PMC6422316; doi:10.1371/journal.pcbi.1006775)
Supplement: S17 Appendix — (DOCX) [file pcbi.1006775.s017.docx]

# **S17 Appendix. Remarks regarding badly identifiable parameters during the fitting of Engel et al data and averaged biological data**

We here discuss the parameters showing low identifiability during the initial parameter fitting in more detail. The sensitivity parameter $b_{{MKC}_{p,64,1}}$ of the transition from this MKC sub-compartment to the pro-platelet compartment, as well as PD parameters *D_ψ_* and $c_{PD,Osteo}$ were slightly overfitted with respective relative errors equal to 1.02, 1.62 and 2.29 respectively.

Parameter *pd_procar_* was poorly identifiable with estimates close to zero during our first fitting attempts. Consequently, we fixed it to 0.

The half-maximal value of platelet uptake *h_s_* by blood vessels showed a large relative standard error too. After detailed likelihood profiling, we found that values less than 2x10^9^ cells per liter per day result in good fits. Thus, the sigmoid function is close to a step function. Since typical platelet levels even in case of severe thrombopoenia are still an order of magnitude larger than this value, the uptake of platelets by blood levels can be assumed constant for the majority of practical applications.
