# Supplementary material for: Modeling individual time courses of thrombopoiesis during multi-cyclic chemotherapy
Source: PLoS Comput Biol. 2019 Mar 6;15(3):e1006775. doi: 10.1371/journal.pcbi.1006775 (PMC6422316; doi:10.1371/journal.pcbi.1006775)
Supplement: S18 Appendix — (DOCX) [file pcbi.1006775.s018.docx]

# **S18 Appendix. Comparison of individual parameter estimates for the NHL-B study with those derived from Engel et al. data**

Since most of the individualized parameters of patients from NHL-B study were constrained by priors derived from the respective parameter fits of Engel et al study (see Table 1 in S13 Appendix), we conducted a sensitivity analysis as follows: we transformed all individual parameter estimates ϕ from both studies to normally distributed quantities ψ with standard deviation ω (see formulas S.13.1, S.13.2). Results were compared using Welch’s t-test (unequal sample size, unequal variances). The resulting p-values, average ϕ, average ψ and ω are presented in the Table 1 of this appendix. Of note, no significant differences were detected between NHL-B and Engel et al.

Table 1. Average ϕ’-s (original parameters), ψ (transformed values) and ω (standard deviation of the transformed values) of the individual parameters are compared between Engel et al. and NHL-B study. P-values correspond to the comparison of ψ values.

| Parameter | Description | Engel et al study | | | NHL-B study | | | p-value |
| --- | --- | --- | --- | --- | --- | --- | --- | --- |
|  |  | Av. ϕ | Av. Ψ | ω | Av. ϕ | Av. ψ | ω |  |
| $d_{{Osteo}_{loss}}$ | Elimination rate of dormant cells due to lack of osteoblast support | 1.42 | 0.220 | 0.674 | 0.760 | -0.274 | 1.02 | 0.331 |
| $b_{S\_act}$ | Steepness of the regulation function of self-renewal probability p | 0.379 | -1.08 | 0.573 | 0.601 | -0.509 | 0.648 | 0.222 |
| $n_{CM}^{unreg}$ | Total number of cell divisions in the late TPO-unregulated CM sub-compartments | 7.87 | -0.0391 | 0.785 | 9.00 | 0.287 | 0.915 | 0.548 |
| *T_PL_* | Transit time of platelets | 261 | 5.56 | 0.0568 | 270 | 5.60 | 0.187 | 0.416 |
| *r_PL,0,nor_* | Ratio of the initial PLC count to the steady state | 1.04 | 0.0403 | 0.0608 | 0.980 | -0.0199 | 0.178 | 0.150 |
| *k_m,TPO_* | saturation of specific TPO elimination  (Michaelis-Menten constant) | 0.264 | -1.54 | 0.755 | 0.258 | -1.36 | 0.623 | 0.721 |
| $\hat{w}_{re}$ | Normalized maximum TPO elimination rate by a single TPO receptor | 1.07 | -0.0203 | 0.506 | 1.06 | 0.0609 | 0.367 | 0.808 |
| *pd_cyclo_* | Toxicity of cyclophosphamide on S compartment | 0.0134 | -4.34 | 0.292 | 9.87E-03 | -4.62 | 0.541 | 0.236 |
